# The guinea pig serves as an alternative model to study human preimplantation development

**DOI:** 10.1038/s41556-025-01642-9

**Published:** 2025-04-04

**Authors:** Jesica Romina Canizo, Cheng Zhao, Sophie Petropoulos

**Affiliations:** 1 Centre de Recherche du Centre Hospitalier, Université de Montréal, Montréal, Canada; 2 Département de Médecine, Molecular Biology Programme, Université de Montréal, Montréal, Canada; 3 Department of Clinical Science, Intervention and Technology, Division of Obstetrics and Gynecology, Karolinska Institutet, Stockholm, Sweden; 4 Department of Gynecology and Reproductive Medicine, Karolinska University Hospital, Stockholm, Sweden

**Keywords:** Embryology, Cell lineage

## Abstract

Preimplantation development is an important window of human embryogenesis. However, ethical constraints and the limitations involved in studying human embryos often necessitate the use of alternative model systems. Here we identify the guinea pig as a promising small animal model to study human preimplantation development. Using single-cell RNA-sequencing, we generated an atlas of guinea pig preimplantation development, revealing its close resemblance to early human embryogenesis in terms of the timing of compaction, early-, mid- and late-blastocyst formation, and implantation, and the spatio-temporal expression of key lineage markers. We also show conserved roles of Hippo, MEK-ERK and JAK-STAT signalling. Furthermore, multi-species analysis highlights the spatio-temporal expression of conserved and divergent genes during preimplantation development and pluripotency. The guinea pig serves as a valuable animal model for advancing preimplantation development and stem cell research, and can be leveraged to better understand the longer-term impact of early exposures on offspring outcomes.

## Abstract (web summary)

Petropoulos and colleagues perform single-cell transcriptomics, spatial, functional and comparative analyses, and show that the guinea pig presents high similarity with early human embryogenesis.

## Main

During human preimplantation development, the initial three lineages, consisting of the trophectoderm (TE), the primitive endoderm (PE) and the epiblast (EPI), are established^1^. This window of development is not only important for establishment of these primary lineages—it also provides the basis for subsequent embryonic development through the continued division and differentiation of cells during gastrulation and organogenesis. Perturbations to the molecular events and regulatory mechanisms (for example, with ‘insults’ or xenobiotics exposure) during this period might influence the developmental trajectories of various tissues and organs, shaping phenotypic outcomes and the long-term health of the individual. Understanding the fundamental aspects of preimplantation development has implications for assisted reproductive technologies and infertility treatments, providing valuable insights into optimizing the conditions that support healthy embryonic and fetal development. Thus, studying this window is imperative for unravelling the intricate processes that lay the foundation for human life, encompassing both the immediate embryonic stages and their far-reaching consequences on overall health and well-being.

Historically, understanding human embryology has relied heavily on mammalian model organisms^2^. Mouse studies have played a pivotal role in unveiling the fundamental principles of early development, but recent advances in low-input methods for investigating genetic and epigenetic mechanisms and efficient techniques for assessing gene function have led to the study of mammalian embryos across various species^3^. This is vital for deciphering the molecular mechanisms involved in pluripotency and the diverse developmental strategies, from an evolutionary perspective^4^. Such knowledge is essential for developing chemically defined culture media to preserve the various pluripotent states in embryo-derived stem-cell lines and to better understand the fundamental principles of early lineage development.

The guinea pig (*Cavia porcellus*) has long been an established model for reproductive studies, and it shares remarkable similarities to humans in terms of general physiology^5^. In the context of reproduction and development, the guinea pig is the only laboratory rodent with a full oestrus cycle that encompasses both follicular and luteal phases, similar to humans, cows, sheep and pigs^6,7^. In early development, the guinea pig preimplantation period is at ~6–7 days, representing one of the only animal models that parallels the duration of human preimplantation^7–10^. Similar to humans, and in contrast to other animal models such as mice, rats, rabbits, pigs, sheep and cows, the guinea pig undergoes interstitial implantation and cavitation^11^ and, following implantation, both the guinea pig and human EPI undergo cavitation and form a bilaminar disc^12^. Furthermore, the guinea pig placenta closely mirrors that of humans, featuring haemomonochorial placentation and proliferating trophoblast cells of similar subtypes to those of humans^13,14^, making the guinea pig an excellent model for understanding trophoblast differentiation and amniogenesis in humans. Finally, their gestation period of 68–72 days can be categorized into trimesters, mirroring the stages of fetal development in humans^15^, and guinea pigs give birth to neuro-anatomically mature offspring^16^. The guinea pig thus provides an animal model that closely recapitulates early human development^7^.

Despite these notable similarities, the guinea pig preimplantation embryo has not been characterized. The ability to leverage the guinea pig as an in vivo model to study the fundamental mechanism(s) underlying early embryogenesis, as well as how early perturbations or exposures to drugs, xenobiotics or exogenous compounds impact these processes and the longer-term health outcomes of the offspring, opens new avenues to enhance our understanding of these inaccessible events in humans. Also, although stem cell-based human embryo models provide an unprecedented opportunity to study early human embryogenesis, it is currently unethical to reimplant these in vivo. This therefore negates our ability to study longer-term health consequences for embryonic health. Recognizing the observed parallels in early development and general physiology between guinea pigs and humans, we aimed to characterize the guinea pig preimplantation embryo with the hope of identifying both conserved and diverging aspects, ultimately introducing an additional small animal model that could be used to better understand preimplantation development and pluripotency.

To address this gap, we conducted a comprehensive characterization of preimplantation development in the guinea pig. We determined the morphokinetics and characterized the temporal development of lineage formation using immunofluorescence in parallel with single-cell RNA-sequencing, identifying key genes and transcription factors that govern blastocyst formation. We also assessed the role of atypical protein kinase C (aPKC) and the evolutionarily conserved Hippo signalling pathway on blastocyst formation, along with MEK/ERK and JAK-STAT signalling on PE formation. We also identified the role of JAK-STAT in mural guinea pig TE formation, and explored the functional role of retinoic acid during the apposition stage of implantation in guinea pig and human embryos. Furthermore, using H3K27me3 loci as a proxy for X chromosome activity, we found a predominant pattern of two foci of H3K27me3 in the guinea pig and human, suggesting incomplete X chromosome inactivation. Finally, we used a comparative biology approach to highlight the similarities and differences in early development among the guinea pig, mouse and human, with a focus on lineage development and naïve pluripotency, highlighting the importance of cross-species studies. Overall, our study identifies the guinea pig as a promising small animal model that can be leveraged to better understand the molecular underpinnings that govern human preimplantation development and, by extension, gastrulation.

## Results

### Characterization of guinea pig preimplantation development

Very little is known about the guinea pig preimplantation embryo. We characterized and staged the embryo based on morphokinetics to determine the timing of key events and establish the spatio-temporal expression of key lineage markers for the trophectoderm (TE), inner cell mass (ICM), EPI and PE. Embryos were collected continuously at 3–6-h intervals following the presence of a positive sperm smear starting at 82–84 h post-fertilization, or embryonic day (E) 3.5 corresponding to the eight-cell stage, until we were unable to flush in vivo embryos (approximately E6). The inability to flush embryos past E6 corroborates that implantation occurs between E6 and E7 in guinea pigs^17^, similar to the timing observed in humans.

**Fig. 1:**
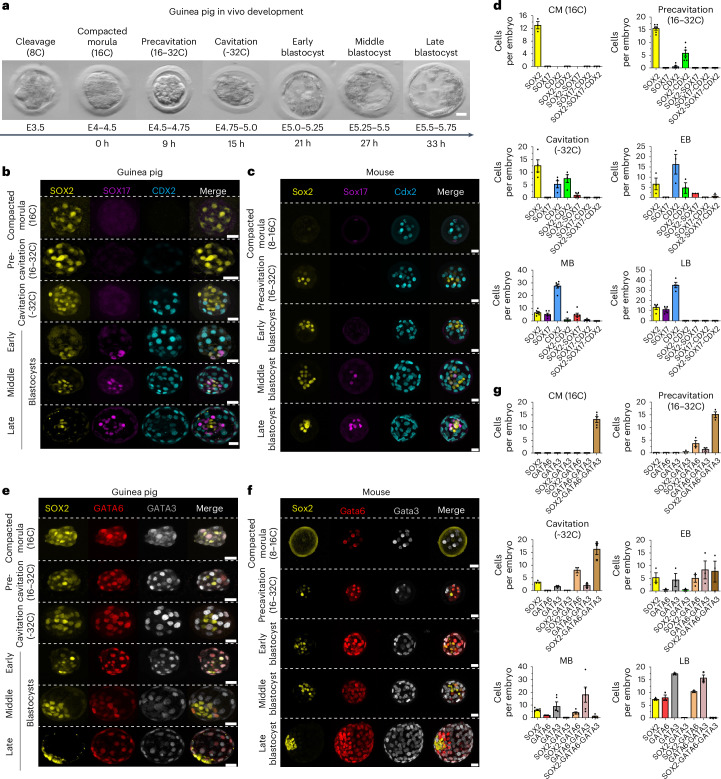
Time-course characterization of in vivo guinea pig preimplantation development. **a**, Bright-field images of embryos captured in vivo at different time points, highlighting the link between developmental stages, morphological phases, embryonic days (E) and the average number of cells observed. **b**,**c**, Time-course immunofluorescence analysis of EPI- (SOX2), PE- (SOX17) and TE- (CDX2) associated markers in guinea pig (**b**) and mouse (**c**) embryos. **d**, Bar graphs quantifying the number of cells per guinea pig embryo of SOX2-, SOX17- and CDX2-only positive cells, as well as their co-expression at different developmental stages: compacted morula (CM, *n* = 3), precavitation (*n* = 6) and cavitation (*n* = 4), and early (EB, *n* = 3), mid (MB, *n* = 6) and late (LB, *n* = 4) blastocysts. **e**,**f**, Immunofluorescence analysis of EPI- (SOX2), PE- (GATA6) and TE- (GATA3) associated markers in guinea pig (**e**) and mouse (**f**) embryos. **g**, Bar graphs quantifying the number of cells per guinea pig embryo of SOX2-, GATA6- and GATA3-only positive cells, as well as their co-expression at different developmental stages: compacted morula (*n* = 5), precavitation (*n* = 3) and cavitation (*n* = 3), and early (*n* = 3), mid (*n* = 5) and late blastocysts (*n* = 3). Data are presented as mean ± s.e.m. Scale bars, 20 µm. Source data

We first staged embryos using bright-field images with a combined approach based on embryo morphology, cell number and embryonic day (Fig. 1a). Compaction was identified by visual inspection of cell boundaries, occurring during the 8–16-cell stage, consistent with previous reports in humans^18^. Using human embryo time-lapse data acquired by Meistermann and colleagues^19^, we aligned compaction (E4–E4.5, 16-cell stage (C)) with *T* = 0 h. Notably, the morphokinetics observed in the guinea pig embryos exhibited temporal progression akin to that seen in humans. The transition from compacted morula to precavitation occurred at *T* = 9 h, followed by cavitation, early blastocyst (EB) occurring at *T* = 21 h, mid blastocyst (MB) at *T* = 27 h, and late blastocyst (LB) at *T* = 33 h post-compaction (*T* = 31 h in human)^19^ (Fig. 1a).

**Extended Data Fig. 1:**
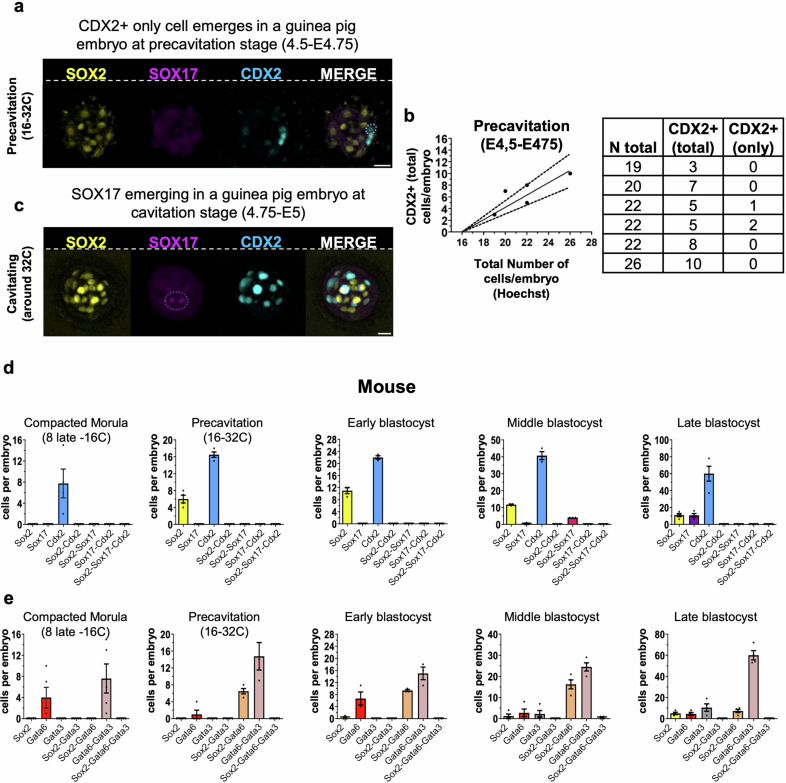
Preimplantation development features of guinea pig and mouse embryos. **a**, Representative immunofluorescence images of EPI (SOX2), PE (SOX17), and TE (CDX2) associated markers during precavitation. The dotted cyan circle indicates an emerging cell with CDX2+ expression only, observed in only 2 of 6 embryos presented at this stage. **b**, Scatter plot depicting the total number of CDX2+ cells per embryo relative to the total number of cells per embryo (Hoechst) (n = 6 embryos collected at E4.5-4.75), along with linear regression illustrating the variability in CDX2 expression at this stage. **c**, Representative immunofluorescence image of EPI (SOX2), PE (SOX17), and TE (CDX2) associated markers during cavitation (E4.75-5). The magenta dotted circle indicates two emerging cells with SOX17+ expression, observed in only 2 of 4 embryos. **d** and **e**, Quantification of immunofluorescence images displayed in Fig. 1c and f. Bar graphs of the number of cells per mouse embryo for (d) Sox2, Sox17 and Cdx2 only positive cells as well as their co-expression at different developmental stages (compacted morula (n = 4), precavitation (n = 4), early (n = 3) mid (n = 3) and late blastocysts (n = 4)) and (e) Sox2, Gata6 and Gata3 only positive cells as well as their co-expression at different developmental stages (compacted morula (n = 5), precavitation (n = 4), early (n = 3) mid (n = 4) and late blastocysts (n = 4). Scale bars: 20 µm. Source data

We next determined the spatio-temporal expression of the key molecular markers for the lineages. Using a comparative biology approach, we performed parallel experiments in mouse embryos and leveraged previously published data from human embryos^20,21^. At the compacted morula stage (E4–E4.5, 16-cell), the guinea pig embryo ubiquitously expressed SRY-Box transcription factor 2 (SOX2) and lacked SRY-Box transcription factor 17 (SOX17) and caudal type homeobox 2 (CDX2) (Fig. 1b), similar to the human embryo at this stage^20^. In contrast, in the mouse, Sox2 expression was restricted to the inner cell population, with Cdx2 exclusively localized to outer cells (Fig. 1c and Extended Data Fig. 1c), consistent with previous reports^22,23^. Moreover, at this stage, all the cells in guinea pig embryos co-expressed SOX2/GATA6/GATA3 (GATA binding protein 6/3; Fig. 1e), whereas human embryos ubiquitously expressed *SOX2*/SOX2 and *GATA6*, with heterogeneous *GATA3*/GATA3 expression^1,3,24,25^. In contrast, in the mouse, distinct inner and outer cells are marked with Sox2 or Sox2/Gata6 and Gata3, respectively (Fig. 1f and Extended Data Fig. 1d). Collectively, the expression patterns of these markers indicate that lineage specification does not commence in the compacted guinea pig embryo, aligning with observations in human, rat and bovine^3^.

CDX2 is a downstream effector of the Hippo signalling pathway, and in the mouse it is expressed starting at the late eight-cell embryo^26–28^ (Fig. 1c and Extended Data Fig. 1b). In contrast, in human, bovine and rat embryos, the emergence of CDX2 expression coincides with cavitation^3,21,29^. Recognizing the dynamic nature of embryo development, we again considered not only developmental time/embryonic day, but also cell number and the presence and size of a blastocoel compartment for staging, consequently categorizing embryos within the 16–32-cell stage as precavitation or cavitation. In the guinea pig, progressive embryo maturation is observed, with distinct expression of CDX2+ cells emerging in the outer cells during the 16–32-cell stage, which corresponds to E4.5–E4.75 (Fig. 1b,d and Extended Data Fig. 1a,b). During precavitation, we observed a dynamic range of CDX2+ expression (3–10 cells) in outer cells, with the majority of the embryos co-expressing SOX2+/CDX2+, suggesting that the TE program is poised during this time, but not initiated (Fig. 1b,d and Extended Data Fig. 1a). In two embryos collected during this stage, distinct CDX2+ cells were observed in only one or two of the outer cells (Extended Data Fig. 1a,b), followed by a drastic increase in the exclusive expression of CDX2+ and a simultaneous decrease of SOX2+ in the outer cells, suggesting that initiation of the ICM–TE occurs during cavitation. The timing of this initial lineage specification is reinforced by the emergence of cells only expressing GATA3+ during cavitation E4.75–E5 in guinea pig embryos (Fig. 1d). In humans, CDX2 is upregulated in E5 blastocysts and is initially coincident with OCT4, indicating a lag in CDX2 expression in the TE lineage^20^, relative to the mouse, similar to what we observe in the guinea pig. Perhaps investigation of human embryos at smaller time intervals (for example, 2–3 h) during E5 would demonstrate a similar transition to what we observed in the guinea pig, with a poised expression of CDX2/SOX2 in outer cells, followed by initiation of CDX2 expression in a subportion of cells and then a rapid spreading of CDX2+ only in the outer cells. Moreover, during cavitation, we captured two embryos (*n* = 2/4) expressing two cells of SOX2+/SOX17+, indicating that the PE program is poised soon after the emergence of ICM–TE (Extended Data Fig. 1c). In guinea pigs, the transition from precavitation to cavitation is rapid (~6 h), underscoring the importance of sequential sampling during this developmental phase to capture dynamic events. This rapid transition is similar to the leading model of lineage specification in humans, where, around E5, embryos shift from an absence of defined lineage to an intermediate ICM–TE and a subsequent emergence of EPI/PE, all occurring during the same developmental day.

During the early blastocyst (EB) stage (E5–E5.25, 28 to 36 cells with the presence of an early blastocoel), the number of cells co-expressing SOX2 and CDX2 is reduced, and we observe the continued ramping up of CDX2+ outer cells, representing a more defined TE lineage. We also begin to see an increase in the expression of SOX17 and a subpopulation of cells solely expressing GATA6 (Fig. 1b,d,e,g). These findings suggest the emergence of the second lineage segregation, consisting of the transition from ICM to EPI/PE. By mid blastocyst (E5.25–E5.5, 40 to 50 cells with a more enlarged blastocoel), the specification of the three lineages is more pronounced, as indicated by the increased number of cells solely expressing one of the well-established molecular marks for their corresponding lineage and the decreased number of cells co-expressing these markers (Fig. 1b,d,f,g). In the mouse (Fig. 1c and Extended Data Fig. 1d), Sox17 is turned on and co-expressed with Sox2 in a salt-and-pepper pattern in the ICM, in contrast to the guinea pig and human, where SOX17 expression is initiated earlier^20^. By the late blastocyst (LB, E5.5–E5.75, 55 to 66 cells with an expanded blastocoel), lineage specification is complete in guinea pigs, and there is no longer the presence of cells co-expressing lineage markers. This aligns with previous reports in humans that demonstrate well-defined lineage segregation before implantation^19,20,30^.

**Extended Data Fig. 2:**
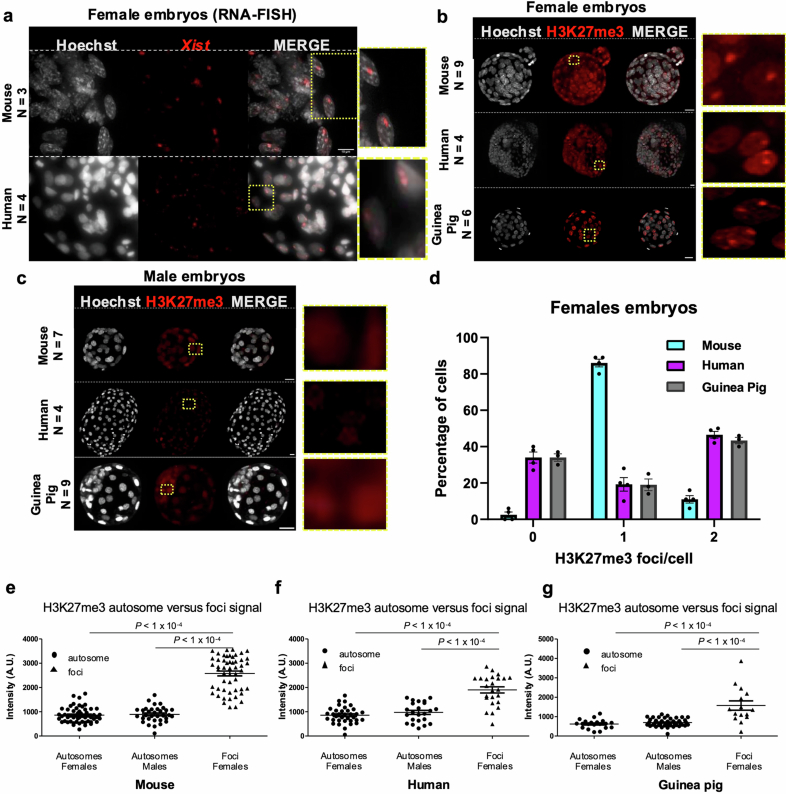
X-Chromosome Status in Mouse, Human, and Guinea Pig Female Blastocysts. **a**, RNA FISH detection of *Xist* (red) and Hoechst (gray) in female embryos of mouse (n = 3) and human (n = 4). **b**, Representative immunofluorescence image of H3K27me3 (red) and Hoechst (gray) in female embryos from mouse (n = 9), human (n = 4) and guinea pig (n = 6). **c**, Representative immunofluorescence image of H3K27me3 (red) and Hoechst (gray) in male embryos from mouse (n = 7), human (n = 4) and guinea pig (n = 9). Dotted yellow square corresponds to the embryo region depicted at a higher magnification for better visualization of H3K27me3 marks.**d**, Bar graph representing the quantification of the number of H3K27me3 foci per cell in mouse (n = 4), human (n = 4) and guinea pig (n = 3) embryos. **e-g**, Scatter plots representing the immunofluorescence intensity of foci vs autosomes in females and males mouse (**e**), human (**f**) and guinea pig (**g**) embryos. Scatter plots are presented with mean ± s.e.m. P values stated in each figure (Kruskal-Wallis test). Scale bar: 20 µm. Source data

Finally, we and others have reported differences between rodents and primates in X-chromosome inactivation (XCI), so we examined it in female guinea pig blastocysts. We first replicated our previous results in human embryos^30^, and observed two X inactive specific transcript (*XIST*) clouds marking the two X chromosomes (Extended Data Fig. 2a). In contrast, in the mouse we observed one cloud of *XIST* in female embryos, marking the inactivated X chromosome, as expected (Extended Data Fig. 2a)^31^. We were unable to detect *XIST* in the guinea pig, probably due to the inability to design probes as the genome is predominantly represented as scaffolds and there are gaps in the assembly. We thus performed immunostaining, using H3K27me3 as a proxy for XCI in mouse, human and guinea pig embryos (Extended Data Fig. 2b,c). In human and guinea pig female blastocysts, a mix of zero, one and two H3K27me3 foci were observed, with no marks observed in male embryos (Extended Data Fig. 2b,d and f,g). In mouse female embryos, however, the majority of cells displayed one H3K27me3, as expected (Extended Data Fig. 2b,d,e). Given the resemblance of H3K27me3 foci in the guinea pig and human, we speculate that the guinea pig may serve as an in vivo model for better understanding the X-chromosome dosage compensation observed in humans and shedding light on the mechanism(s) involved.

### Transcriptome dynamics of preimplantation guinea pig embryos

**Extended Data Fig. 3:**
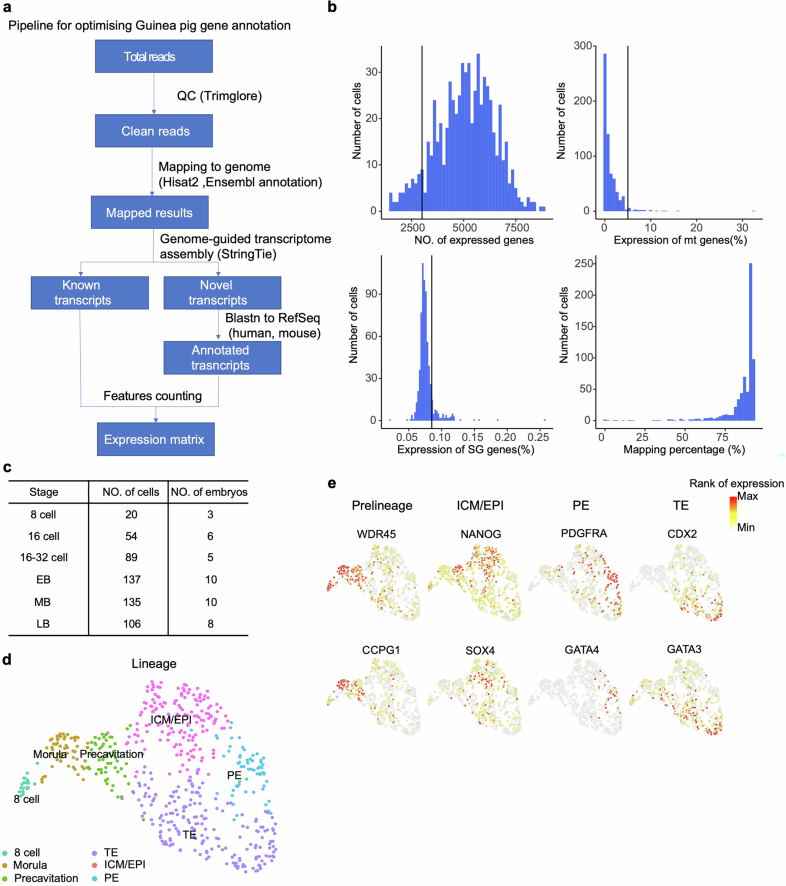
Analysis pipeline, quality control and cell number statistics used for the guinea pig scRNA-seq dataset. **a**, Schematic diagram of optimized guinea pig gene annotation. **b**, Histograms display the number of expressed genes, the proportion of mitochondrial (mt) gene expression, the proportion of stress granule (SG) gene expression, and the total mapping percentage. Quality control cutoffs are indicated by black lines. **c**, Number of cells belonging to each lineage included in the analysis. **d**, Two-dimensional UMAP representations of 541 single-cell (n = 42 embryos) transcriptomes from guinea pig preimplantation embryos, with colours indicating the detailed lineages. **e**, UMAP plot of cells showing selected marker gene expression. Prelineage genes include *WDR45* and *CCPG1*, ICM/EPI genes include *NANOG* and *SOX4*, PE genes include *PDGFRA* and *GATA4* and TE genes include *CDX2* and *GATA3*. Source data

A limitation of immunofluorescence is the number of lineage markers that can be assessed simultaneously. To obtain comprehensive spatio-temporal gene expression and lineage dynamics, we utilized single-cell RNA-sequencing (scRNA-seq) on individual blastomeres isolated from E3.5–E6 guinea pig embryos. Following optimized transcriptome mapping and quality control (Methods, Supplementary Fig. 1 and Extended Data Fig. 3a), we retained 541 high-quality single-cell transcriptomes from 42 embryos (Extended Data Fig. 3a–c and Supplementary Table 1).

**Fig. 2:**
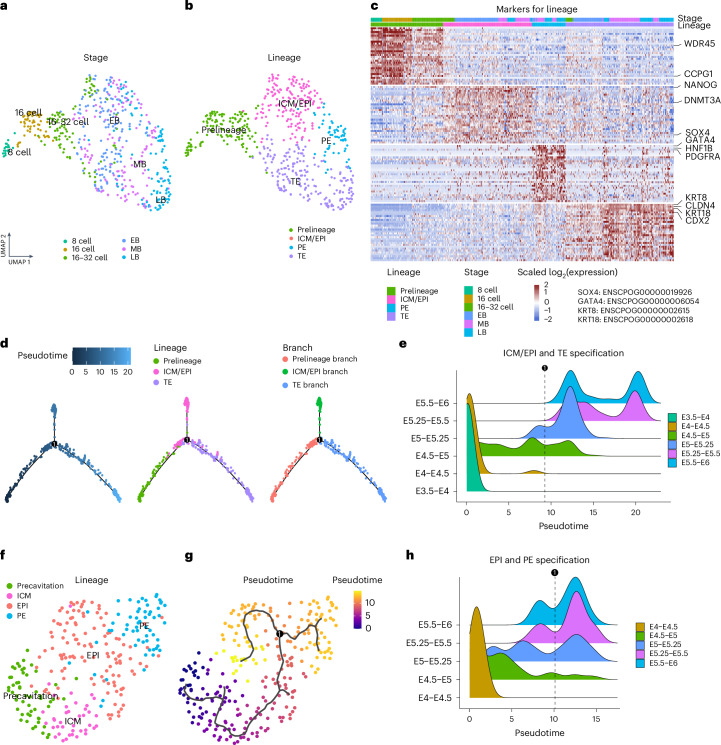
Characterization of preimplantation development in the guinea pig with scRNA-seq. **a**,**b**, Two-dimensional UMAP representation of 541 single-cell transcriptomes from guinea pig preimplantation embryos. Developmental stages (**a**) and lineages (**b**) are indicated by colours, respectively. **c**, Heatmap showing the top 30 marker genes for prelineage, ICM/EPI, PE and TE cells (from 42 embryos). Selected well-known or human-conserved lineage genes are labelled on the right. **d**, Two-dimensional diffusion map showing the developmental trajectory of prelineage, ICM/EPI and TE cells (*n* = 481 cells). Pseudotime, lineages and branches are indicated by colours, respectively. **e**, Ridge plot showing the distribution of single cells of **d** along the pseudotime axis, stratified by embryonic days. The dashed line on the ridge plot represents the bifurcation into TE and ICMs. **f**,**g**, Two-dimensional UMAP representations of cells from precavitation, ICM, EPI and PE cells (from 34 embryos). Embryo lineage (**f**) and pseudotime (**g**) are indicated by colours, respectively. **h**, Ridge plot showing the distribution of single cells of **g** along the pseudotime axis, stratified by embryonic days. The dashed line on the ridge plot represents the bifurcation into EPI and PE. Source data

To better characterize the preimplantation development and assign cell identity based on gene expression, we performed dimensionality reduction using uniform manifold approximation and projection (UMAP), providing a clear visualization. Developmental time accounts for the primary factor driving expression variation and includes 8-cell, 16-cell and 16–32-cell (which encompasses both precavitation and cavitating embryos) stages, EB, MB and LB (Fig. 2a,b and Supplementary Fig. 2a,b). Next, we identified lineage populations by leveraging well-known markers from human and mouse studies for each lineage (TE, ICM, EPI and PE)^19,30,32–34^, including Nanog Homeobox (*NANOG*) for ICM/EPI, platelet-derived growth factor receptor alpha (*PDGFRA*) and GATA binding protein 4 (*GATA4*) for PE, and *CDX2* and *GATA3* for TE (Fig. 2c and Extended Data Fig. 3d,e). Additionally, human-conserved markers such as WD repeat-containing protein 45 (*WDR45*) and cell cycle progression protein 1 (*CCPG1*) for prelineage, and Sry-box transcription factor 4 (*SOX4*) for ICM/EPI were identified among the top 30 lineage markers in guinea pig (Fig. 2c, Extended Data Fig. 3e and Supplementary Table 2). Finally, cell identities were assigned as prelineage (8 cell, 16 cell and precavitation), TE, ICM/EPI and PE (Fig. 2b,c and Extended Data Fig. 3d,e). Consistent with human studies^19,30,32^, we were unable to distinguish a unique cluster of cells corresponding to the ICM in this analysis (from resolution 0.4 to 1.4 using the ‘FindCluster’ function; Supplementary Fig. 2c) without further integration, and as such have labelled the lineage as ‘ICM/EPI’, which we resolve in the next section.

### Lineage specification and ICM resolution in the guinea pig

**Extended Data Fig. 4:**
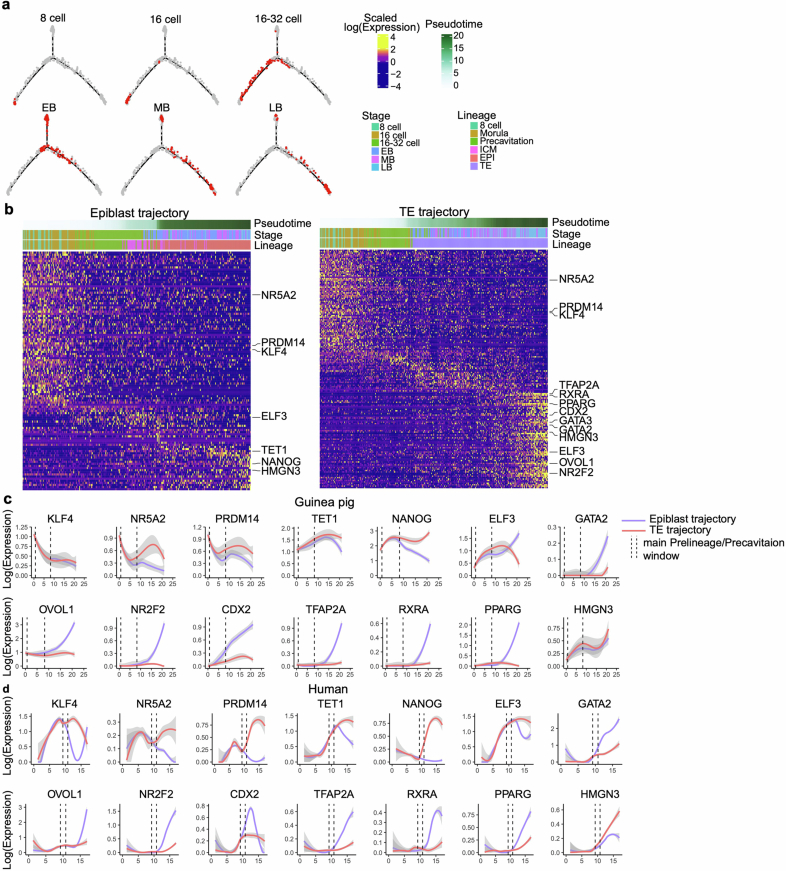
Pseudotime inference for the ICM–TE developmental trajectory and transcription factor gene expression along the EPI and TE trajectories. **a**, Cells from different developmental stages highlighted along the trajectory. **b**, Heatmap showing the expression of transcription factor genes significantly associated with pseudotime trajectories. **c**, and **d**, Expression dynamics (pseudotime) of selected transcription factor genes along EPI and TE trajectories in guinea pigs (**c**) and humans (**d**). The confidence interval (error bands, 95%) is represented by bandwidth. The center measure and confidence intervals were calculated using the ‘loess’ function with default parameters in R software. Lineage trajectories are indicated by purple (EPI) and pink (TE). Source data

To confirm the timing of lineage specification based on the spatio-temporal expression of key molecular markers, we leveraged the scRNA-seq data and performed pseudotime inference. To validate the timing of the first lineage specification (ICM–TE), we determined the developmental trajectory of prelineage cells (8 cell, 16 cell and precavitation 16–32 cell), ICM/EPI cells (as previously mentioned) and TE cells by applying a two-dimensional diffusion map to the top 450 differentially expressed genes (DEGs) among them (Fig. 2d and Extended Data Fig. 4a). Next, indicated by the pseudotime bifurcation point of the ICM and TE branch, we examined the distribution of cells stratified by embryo stage and observed that the ICM–TE segregation occurs during E4.5–5 (Fig. 2d,e), corresponding to the transition from precavitation to cavitation, and in alignment with our immunostaining (Fig. 1).

The construction of developmental trajectories provided an opportunity to explore key transcription factor dynamics involved in the development of the EPI and TE lineages in guinea pig. In the EPI and TE trajectories, 113 and 168 transcription factors, respectively, showed dynamic expression along the inferred pseudotime (Extended Data Fig. 4b and Supplementary Table 3). Transcription factors such as PR/SET domain 14 (*PRDM14*) and KLF franscription factor 4 (*KLF4*) exhibit high expression during the morula stages, with a progressive decrease in expression as the EPI and TE lineages develop in the guinea pig (Extended Data Fig. 4b,c). In contrast, in human, *KLF4* emerges during precavitation and remains elevated in only the EPI (Extended Data Fig. 4d). In both species, there is a conserved increase in the expression of *NANOG* and Tet methylcytosine dioxygenase 1 (*TET1*), as well as well-known TE-driving transcription factors such as GATA binding protein 2 (*GATA2*), *CDX2*, nuclear receptor subfamily 2 group F member 2 (*NR2F2*) and transcription factor AP-2 alpha (*TFAP2A*), within the EPI and TE trajectories, respectively, suggesting that these transcription factors may also play important roles in guinea pigs (Extended Data Fig. 4b–d). Notably, high mobility group nucleosomal binding domain 3 (*HMGN3*) exhibits increased expression in both the EPI and TE trajectories, consistent with the pattern observed in human and non-human primates^25,30,34–36^. Together, this analysis provides insight into key transcription factors that may drive differentiation during early guinea pig development.

**Extended Data Fig. 5:**
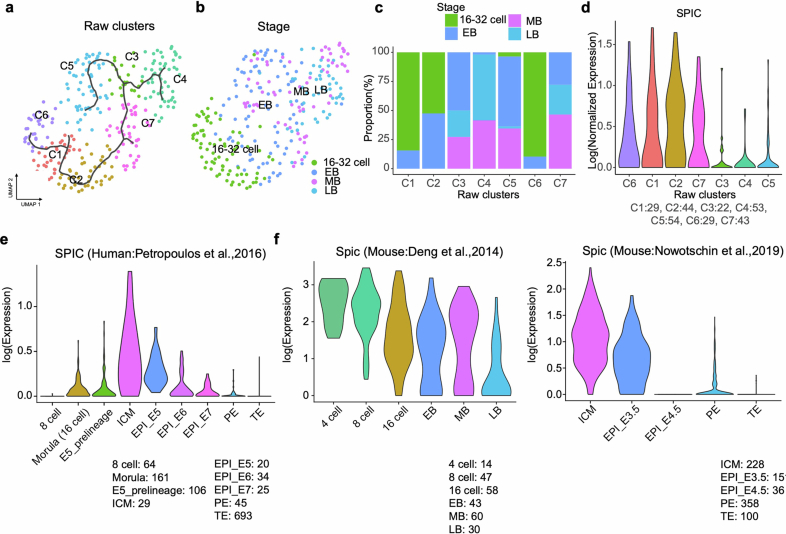
Resolving ICM cells in the guinea pig preimplantation embryo. **a** and **b**, Two-dimensional UMAP representation of 274 cells (n = 34 embryos) from precavitation, ICM, EPI, and PE. Raw clusters (**a**) and stages (**b**) are indicated by different colours. **c**, Bar plot showing the proportion of stages in each cluster from (**a**). **d**, Violin plots illustrating *SPIC* expression in each cluster from (**a**)**. e** and **f**, Violin plot showing *SPIC* expression in the human from reanalysis of Petropoulos et al., 2016 dataset (**e**), and in the mouse datasets from Deng et al., 2014 and Nowotschin et al., 2019. **f**, The cell numbers used to generate the violin plots are indicated for each stage and species. Source data

As mentioned above, we were unable to identify a distinct cluster of cells corresponding to a bona fide ICM when considering all cells. We next interrogated the ICM/EPI population by subsetting the cells belonging to the precavitation, ICM/EPI and PE populations, and performed clustering and pseudotime inference using the top 1,500 variable genes among them (Fig. 2f,g and Extended Data Fig. 5a,b). Among the seven identified clusters (Extended Data Fig. 5a), cluster 2 was confirmed as the ‘ICM’ based on its stage distribution and inferred pseudotime (Methods and Extended Data Fig. 5a–c). This identification was further supported by the high expression of *SPIC*, a marker of ground-state pluripotency and conserved transcription factor in the ICM^37,38^. We also found *SPIC* to be highly expressed in the prelineage and ICM, with a dramatic decline in the EPI, a pattern that was conserved in guinea pig, mouse and human (Extended Data Fig. 5d–f)^30,39,40^. Finally, examining the distribution of cells inferred by pseudotime, stratified by embryo stage, we found that EPI and PE specification occurred during E5–E5.25, which corresponds to the EB (Fig. 2h). These analyses corroborated our immunostaining findings, confirming that a progressive segregation of the ICM into EPI and PE occurs during the EB.

Next, to chart pluripotency in the guinea pig, we analysed the gene expression of well-known markers related to human naïve, primed and core pluripotency in ICM, EPI, PE and TE. We observed a signature of naïve and core pluripotency in guinea pig embryos that was similar but not identical to what is seen in human embryos (Supplementary Fig. 3). Throughout blastocyst development, genes related to primed pluripotency were very lowly expressed or absent in both species. Furthermore, similar developmental expression patterns for DNA methyltransferase 3 beta (*DNMT3B*) and *PRDM14* were observed in both species, implying a conserved role in the embryo. The overlapping pluripotency signatures between guinea pig and human embryos underscore potentially conserved mechanisms related to the establishment and progression of the different states of pluripotency. Further exploration in this direction could provide valuable insights towards a broader understanding of pluripotency across diverse species.

### Signalling pathways involved in lineage specification

**Fig. 3:**
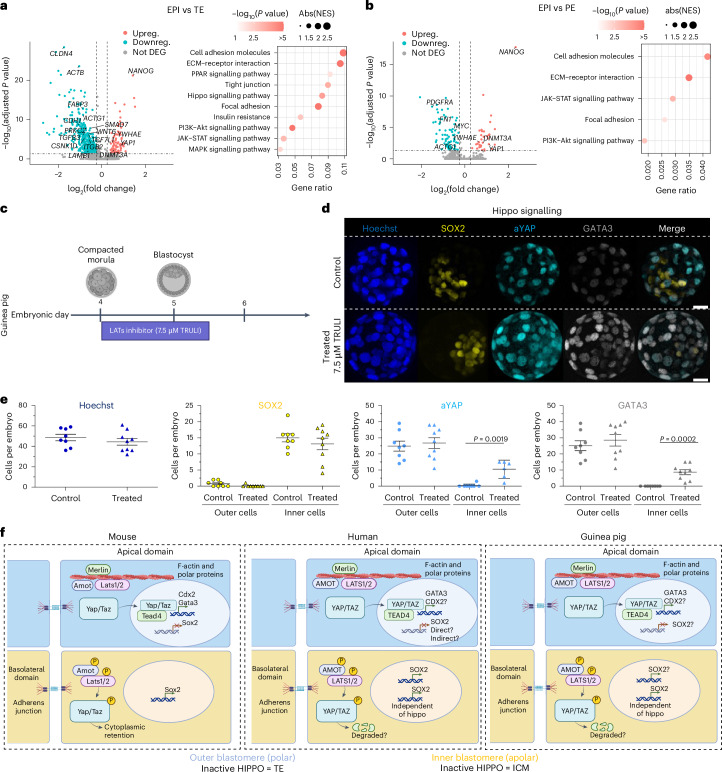
Signalling pathways related to guinea pig preimplantation embryo development. **a**,**b**, Volcano plot showing the fold change in expression and Bonferroni-adjusted *P* values (two-sided) of DEGs calculated by Wilcoxon test when comparing EPI and TE (**a**) and EPI and PE (**b**). Dot plots of KEGG terms with results from GSEA are also shown, with *P* values (one-sided, permutation test) less than 0.05 indicated. Dot size indicates the absolute values of normalized enrichment score (NES), colour represents the *P* value, and the *x* axis shows the ratio of DEGs corresponding to each KEGG term. **c**, Schematic of the TRULI treatment protocol. **d**, Representative immunofluorescence image of SOX2 (yellow), aYAP (cyan), GATA3 (grey) and Hoechst (blue) in embryos treated with control (DMSO) and LATS inhibitor (7.5 µM TRULI). **e**, Scatter plots showing the total numbers of cells per embryo (Hoechst) and the numbers of cells per embryo for the indicated markers in control (*n* = 8) and TRULI-treated embryos (*n* = 9), *P* values are stated in each figure (two-tailed Mann–Whitney test). Scale bars, 20 µm. **f**, Schematics of the evolutionarily conserved function of the Hippo signalling pathway in TE formation of mouse human and guinea pig embryos. Notably, in contrast to the mouse, in the human and guinea pig embryo, inhibition of LATS1/2 does not abolish SOX2 expression in inner cells. Schematic created with BioRender.com. Panel **f** adapted from ref. ^3^ under a Creative Commons license CC BY 4.0. Source data

We next determined signalling pathways involved in lineage segregation and/or maintenance, focusing on comparisons between EPI and TE, and EPI and PE in the guinea pig embryo (Fig. 3a,b and Supplementary Table 4), which we then compared with human and mouse using a similar approach (Supplementary Fig. 4 and Supplementary Table 4)^30,40^. Based on gene set enrichment analysis (GSEA), we found the Hippo signalling pathway or specific components of this pathway implicated in EPI versus TE in the guinea pig, human and mouse (Fig. 3a and Supplementary Fig. 4b,c), supporting the evolutionarily conserved role of Hippo signalling in TE formation^3^. Additionally, the JAK-STAT signalling pathway and specific components of this pathway were implicated in EPI versus PE formation in the guinea pig (Fig. 3b and Supplementary Fig. 4c), suggesting that the JAK-STAT pathway is involved in PE specification, similar to what is known in the mouse^41^. In the human embryo, the JAK-STAT pathway was not significant, and its role on PE specification remains to be determined. In addition, the PI3K-Akt signalling pathway was identified in guinea pigs, mice and humans when comparing EPI and PE. However, the majority of the individual components contributing to the importance of this pathway are known to be involved in other signalling pathways such as *Myc* and *GSK3b*, or shared components of FGF/MEK-ERK signalling^41^. Although consistent with previous work in the human embryo,_,_ we identified two signalling components specific to PI3K-Akt signalling, *INSR* and *IGF1*, which were upregulated and downregulated, respectively, in the EPI^42,43^. Whether the PI3K-Akt alone or crosstalk with other signalling pathways (including FGF/MEK-ERK signalling) is involved in PE specification needs further functional evidence.

**Extended Data Fig. 6:**
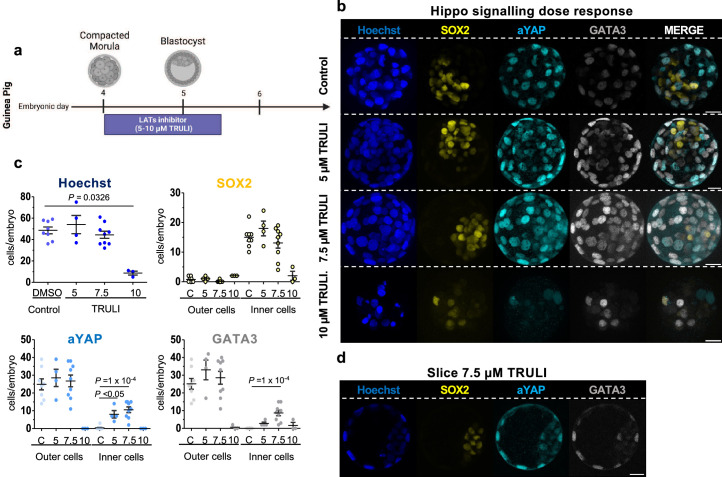
Dose-response of Hippo signalling pathway in guinea pig embryos. **a**, Schematic protocol used with guinea pig embryos. **b**, Representative immunofluorescence image of SOX2 (yellow), aYAP (cyan), GATA3 (gray) and Hoechst nuclear staining (blue) in control (DMSO) and LATS inhibitor (TRULI) treated embryos from compacted to morula to mid-blastocysts. **c**, Scatter plot showing the total number of cells per embryo (Hoechst stained) and the number of cells per embryo for the indicated markers in control (n = 8) and TRULI at 5 µM (n = 4), 7.5 µM (n = 9) and 10 µM (n = 3); P values stated in each figure (Kruskal-Wallis test with Dunn’s post-test). **d**, Representative slice showing aYAP (cyan) and GATA3 (gray) expression in the inner cells. Schematic created in BioRender.com. Scatter plots with mean ± s.e.m. Scale bars: 20 µm. Source data

GSEA analysis is based solely on transcriptome data, so we investigated the functional involvement of selected pathways in lineage formation using small molecules. We explored the functional role of Hippo signalling kinases (large tumour suppressor kinase 1/2, LATS1/2) in the guinea pig compared to mouse embryos using the specific LATS inhibitor, TRULI^3,44^. Using a dose–response experiment (Extended Data Fig. 6 and Supplementary Fig. 5), we determined that 7.5 µM and 5 µM TRULI were the optimal doses for the guinea pig and mouse, respectively, reproducing previous work in the mouse^3^. Both the compacted guinea pig (16 cell) and mouse (8 cell) embryos were cultured with either TRULI or dimethyl sulfoxide (DMSO) as control until the blastocyst stage (Fig. 3c and Supplementary Fig. 6a). In mouse embryos, consistent with previous reports^21,28,45^, Lats1/2 inhibition led to a complete ablation of Sox2 expression and ectopic expression of Gata3 and active Yap (aYap) in the inner cells (Supplementary Fig. 6b,c). In contrast, in the guinea pig we observed a trend towards downregulation of SOX2, accompanied by ectopic expression of GATA3 and aYAP (Fig. 3d,e, *P* < 0.001 and Extended Data Fig. 6d), mirroring reports in human embryos^3^. Additionally, in guinea pig blastocysts, the number of outer cells expressing aYAP and GATA3 remained unaffected (Fig. 3e), similar to what is reported in humans and mice^3^ (Supplementary Fig. 6c). Upstream of Hippo signalling, atypical protein kinase C (aPKC) connects outer-cell polarity with TE specification^21^. We determined that inhibition (using Gö6983 or CRT0066854) in guinea pig and mouse embryos led to developmental arrest at the cavitation stage, preventing blastocyst formation (Supplementary Fig. 7; details are provided in the Methods). Although this effect was consistent across both species, the response differed, as guinea pig embryos retained expression of key outer-cell markers, unlike previous findings in mouse and human embryos treated with another PKC inhibitor, CRT0276121^21^. Nonetheless, our data support that the molecular cascade that initiates the TE program in human, cow, rat and mouse embryos^21^ is also evolutionarily conserved in the guinea pig.

**Fig. 4:**
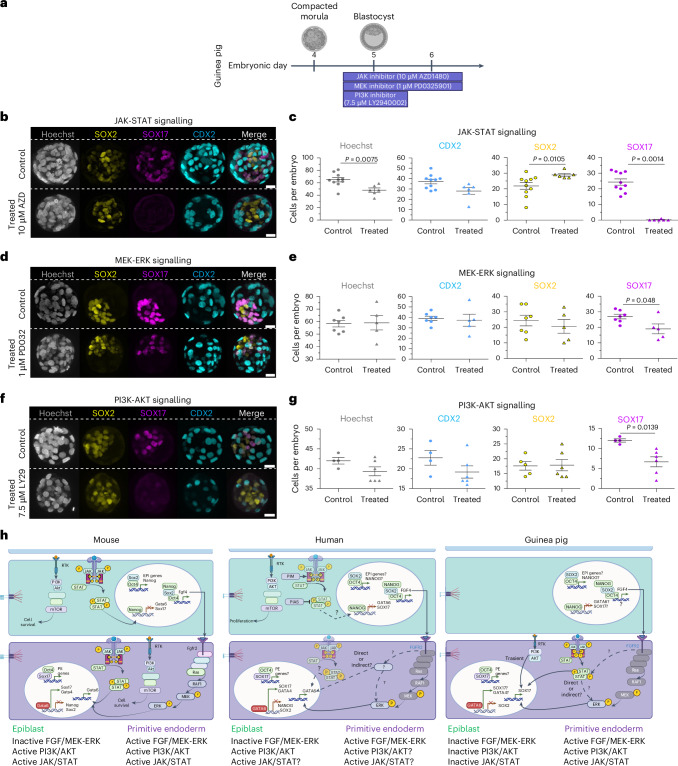
JAK-STAT, MEK-ERK and PI3K signalling pathways in guinea pig embryos. **a**, Schematic of treatment protocol for individual small molecules. **b**, Representative immunofluorescence images of SOX2 (yellow), SOX17 (magenta), CDX2 (cyan) and Hoechst (blue) in control- (DMSO) and JAK inhibitor- (10 µM AZD1480) treated embryos. **c**, Scatter plots showing the quantification of the total numbers of cells per embryo (Hoechst) and the numbers of cells for the indicated markers in control- (*n* = 10) and AZD1480- (*n* = 6) treated embryos. *P* values are stated in the figure (two-tailed Mann–Whitney test). **d**, Representative immunofluorescence images of SOX2 (yellow), SOX17 (magenta), CDX2 (cyan) and Hoechst (blue) in control- (DMSO) and MEK inhibitor- (1 µM PD0325901) treated embryos. **e**, Scatter plots showing the total numbers of cells per embryo (Hoechst) and the numbers of cells for the indicated markers in control- (*n* = 7) and PD0325901- (*n* = 5) treated embryos. *P* values are stated in the figure (two-tailed Mann–Whitney test). **f**, Representative immunofluorescence images of SOX2 (yellow), SOX17 (magenta), CDX2 (cyan) and Hoechst nuclear staining (grey) in control- (DMSO) and 7.5 µM LY294002-treated embryos at 24 h. **g**, Scatter plots showing the total numbers of cells per embryo (Hoechst) and the numbers of cells per embryo for the indicated lineage markers for 24 h for control (*n* = 4) and PI3K-inhibited (*n* = 6) embryos. *P* values are stated in the figure (two-tailed Mann–Whitney test). Scatter plots present the mean ± s.e.m. Scale bars, 20 µm. **h**, Schematics of selected signalling pathways in mouse, human and guinea pig EPI and PE specification, showing a cross-species comparison of FGF/MEK-ERK, PI3K-AKT and JAK/STAT in EPI and PE formation. For the human and guinea pig embryos, signalling components of the MEK-ERK pathway are in grey as this pathway contributes to PE expansion, but a lineage switch with EPI is not observed. Schematic created with BioRender.com. Panel **h** adapted with permission from ref. ^67^, Elsevier. Source data

**Extended Data Fig. 7:**
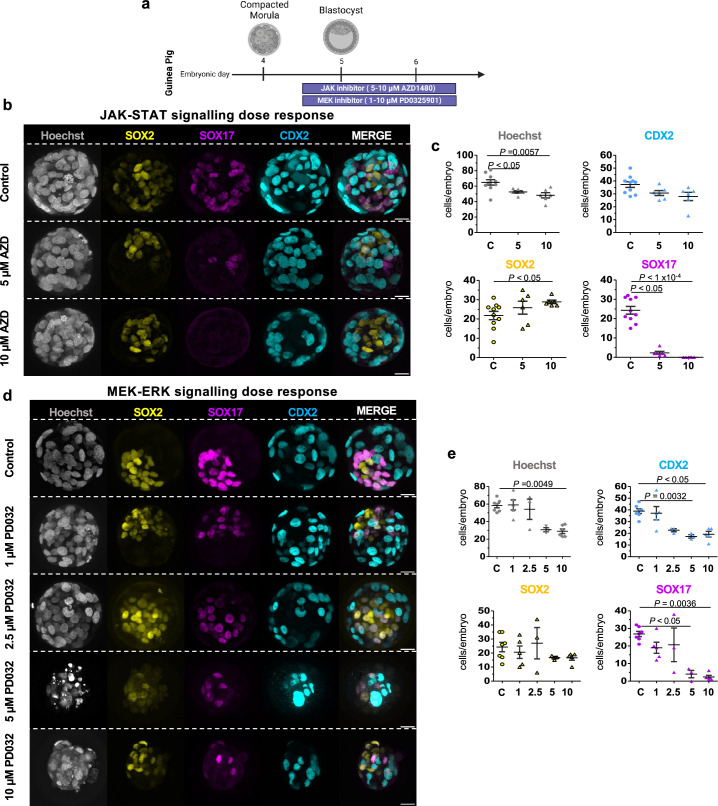
Dose-response of JAK-STAT and MEK-ERK signalling pathways in guinea pig embryos. **a**, Schematic protocol for individual small molecules used with guinea pig embryos. **b**, Representative immunofluorescence image of SOX2 (yellow), SOX17 (magenta), CDX2 (cyan) and Hoechst nuclear staining (gray) in control (DMSO) and JAK-STAT inhibitor (AZD1480) treated embryos. **c**, Scatter plot showing the total number of cells per embryo (Hoechst stained) and the number of cells per embryo for the indicated markers in control (n = 10) and AZD1480 at 5 µM (n = 6) and 10 µM (n = 6); P values stated in each figure (Kruskal-Wallis test with Dunn’s post-test). **d**, Representative immunofluorescence image of SOX2 (yellow), SOX17 (magenta), CDX2 (cyan) and Hoechst nuclear staining (gray) in control (DMSO) and MEK-ERK inhibitor (PD0325901) treated embryos. **e**, Scatter plot showing the total number of cells per embryo (Hoechst stained) and the number of cells per embryo for the indicated markers in control (n = 7) and PD0325901 at 1 µM (n = 5), 2.5 µM (n = 3), 5 µM (n = 3) and 10 µM (n = 5); P values stated in each figure (Kruskal-Wallis test with Dunn’s post-test). Schematic created in BioRender.com. Scatter plots with mean ± s.e.m. Scale bars: 20 µm. Source data

Next, we investigated the role of JAK-STAT signalling in PE formation using an optimal dose of 10 μM for JAK inhibitor AZD1480, as determined by dose–response experiments (Extended Data Fig. 7a–c). Corroborating our GSEA analysis (Fig. 3b), SOX17+ cells were entirely absent in AZD1480-treated embryos, whereas SOX2+ cell numbers increased proportionally (Fig. 4b,c), in line with observations in the bovine embryo^46^ and partially with that in the mouse, where inhibition of JAK-STAT signalling reduces both NANOG+ EPI and GATA6+ PE^41^. Furthermore, in the guinea pig, the number of CDX2+ cells tended to decrease (*P* = 0.0573), indicating an impact on the TE, which we explore further in the next section. Together, these results support the involvement of JAK-STAT signalling in guinea pig lineage formation.

In line with what is known in the mouse, our DEG analysis implicated the mitogen-activated protein kinase (MAPK) signalling pathway between the EPI and PE in the guinea pig (Fig. 3b and Supplementary Fig. 4c). In mouse and rat preimplantation embryos, inhibition of the Fgf/MEK-ERK pathway redirects all cells of the ICM to the EPI fate, bypassing the PE^45,47–49^. However, MEK inhibitor PD0325901 (PD032) has different effects in other mammals. For instance, in rabbit^50^ and bovine^51^ embryos, PD032 completely abolishes the expression of the PE marker SOX17, suggesting an impairment of PE formation without the occurrence of lineage switching. In human and porcine embryos, inhibiting MAPK signalling with a low dose of PD032 does not abolish PE cells, but instead reduces their number^48,52^. We first confirmed the known effect of PD032 in mouse embryos (Supplementary Fig. 6f,g). In the guinea pig, following a dose–response investigation (Extended Data Fig. 7a,d,e), we observed that 1 µM did not affect blastocyst formation or the total number of cells compared to control, but did significantly reduce the number of SOX17+ cells, although the number of SOX2+ or CDX2+ cells remained unaffected (Fig. 4d,e). This suggests that the MAPK signalling pathway in guinea pigs is involved in PE expansion, as observed in human^48^, rabbit^50^ and bovine^51^ embryos, but in contrast to mice (Supplementary Fig. 6f,g) and rats^45,47–49^.

Finally, we investigated the role of PI3K-Akt signalling, highlighted by GSEA as relevant to EPI versus PE formation in guinea pig, human and mouse embryos (Fig. 3b). In mouse embryos, PI3K-Akt stabilizes Gata6 protein levels to support PE specification alongside FGF/ERK signalling^53^. Using LY294002 (LY29) in guinea pigs (5, 7.5 and 10 µM) and mice (5 and 10 µM) for 48 h, we found no significant impact on total cell numbers, although 10 µM LY29 tended to reduce Sox17+ cells in mice (Supplementary Fig. 8). A 24-h treatment with 7.5 µM LY29 in the guinea pig embryo led to a significant decrease in SOX17+ cells without affecting the number of SOX2+ cells (Fig. 4f–h), indicating PI3K-Akt’s role in PE expansion rather than lineage specification. This differs from its function in porcine^52^ and human^42^ embryos and blastoids^54^, where PI3K-Akt primarily regulates ICM proliferation via NANOG+ cells without affecting the number of SOX17+ cells. These species–specific differences underscore the complexity of PI3K-Akt signalling, supporting the need for further studies to unravel its conserved and divergent roles in blastocyst development. Overall, similar signalling pathways are involved in EPI/PE formation in guinea pig, human and mouse embryos, but with nuanced differences (Fig. 4h).

### Characterizing mural and polar TE sublineages

**Fig. 5:**
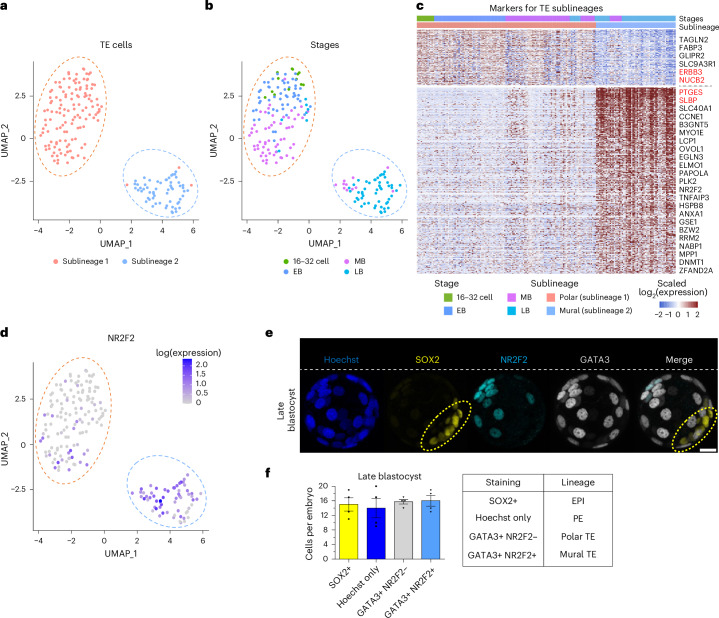
Profiling the mural and polar TE sublineages in the guinea pig embryo. **a**,**b**, Two-dimensional UMAP of guinea pig TE cells (from 29 embryos) stratified by sublineage (**a**) and developmental stage (**b**), with colours corresponding to sublineages and the developmental stages, respectively. **c**, Heatmap representation displaying the DEGs between TE sublineages. Genes that were also significantly differentially expressed between human mural and polar TE cells are labelled on the right. Red and black colours indicate the same and opposite expression patterns, respectively. **d**, Feature plot displaying the expression of *NR2F2* in guinea pig TE cells (from 29 embryos). **e**, Immunofluorescence image of a representative in vivo guinea pig embryo at the late blastocyst stage labelled with Hoechst (nucleus), SOX2+ (EPI), NR2F2+ GATA3+ (mural TE) and NR2F2− GATA3+ (polar TE). The dotted oval outlines the EPI cells within the embryo. **f**, Quantification of cells per embryo expressing SOX2+ (EPI), NR2F2+ GATA3+ (mural TE) and NR2F2− GATA3+ (polar TE) in guinea pig in vivo late blastocysts. Cells with only Hoechst staining represent the PE. Bar graphs present the mean ± s.e.m. of *n* = 4 replicates. Scale bar, 20 µm. Source data

In the human embryo, the polar TE initiates attachment and implantation into the uterine wall, whereas in the mouse and guinea pig, it is the mural TE^19,55^. Despite the presence of mural–polar TE sublineages in guinea pig embryos, their molecular signatures remain unexplored. As the TE plays a unique role in mammalian development by forming the embryonic portion of the placenta^27^, we investigated the presence of mural–polar sublineages and their corresponding gene signatures. First, we selected all TE cells from cavitation to late blastocyst and performed UMAP dimensional reduction, identifying two distinct clusters (Fig. 5a,b). Differential expression analysis between the two clusters (TE sublineage 1 and TE sublineage 2) identified 526 DEGs including the well-known human polar marker NR2F2 (Fig. 5c,d and Supplementary Table 5)^19^. To validate the localization of each TE sublineage in the guinea pig, we checked the expression of NR2F2 in the guinea pig and found it to be localized to the mural TE sublineage (TE sublineage 2, Fig. 5e,f).

**Fig. 6:**
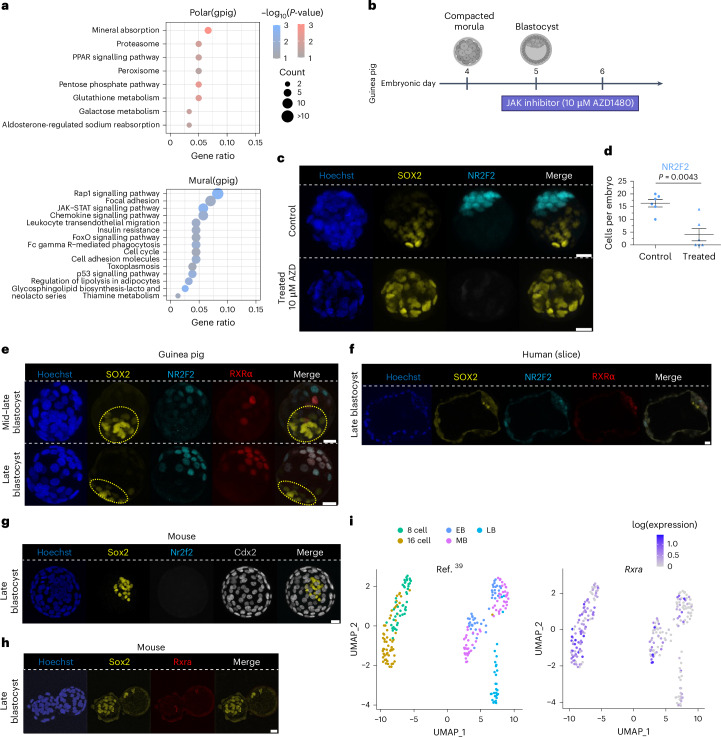
Implantation pole analysis of guinea pig, human and mouse embryos. **a**, Dot plot illustrating the enriched KEGG pathways for DEGs between mural and polar TE cells in guinea pig (*P < 0.05, one-sided, calculated by permutation test). Colour and size indicate the significance and number of DEGs in each pathway. **b**, Schematic of the JAK-STAT inhibition treatment protocol. **c**, Representative immunofluorescence images of SOX2 (EPI, yellow), NR2F2 (mural TE, cyan) and Hoechst nuclear staining (blue) in control- (DMSO) and JAK-STAT inhibitor- (10 µM AZD1480) treated guinea pig embryos. **d**, Scatter plot showing the total number of cells per embryo (Hoechst stained) and the number of cells per embryo for the respective lineage markers analysed in control- (*n* = 6) and AZD1480- (*n* = 6) treated embryos. The *P* value is stated in the figure (two-tailed Mann–Whitney test). **e**, Representative immunofluorescence images of SOX2 (yellow), NR2F2 (cyan), RXRα (red) and Hoechst nuclear staining (blue) in guinea pig embryos at mid and late blastocyst (*n* = 4). **f**, *Z*-stack slice from a representative immunofluorescence image of SOX2 (yellow), NR2F2 (cyan), RXRα (red) and Hoechst nuclear staining (blue) in human embryos at late blastocyst (*n* = 4). **g**, Immunofluorescence representation of a late mouse blastocyst analysing Sox2 (yellow), Nr2f2 (cyan) and Cdx2 (grey); the total cells are visualized by Hoechst (blue) (*n* = 5). **h**, Immunofluorescence representation of a mouse late blastocyst (90-cell embryo, E4.5) analysing Sox2 (yellow) and Rxrα (red), with the total number of cells visualized by Hoechst (blue) (*n* = 5). **i**, UMAP analyses showing the developmental progression of mouse cells from the eight-cell stage to late blastocyst (left) and *Rxrα* expression (right) during preimplantation development. Data from ref. ^39^. Scale bars, 20 µm. Source data

Next, we conducted KEGG pathway enrichment analysis on the significant DEGs between mural and polar cells and observed a significant upregulation of the peroxisome proliferator-activated receptor (PPAR) signalling pathway and metabolism-related pathways, including glutathione and galactose metabolism in polar cells (Fig. 6a). Additionally, significant increases in the JAK-STAT pathway, chemokine signalling pathways and cell adhesion molecules were noted in mural cells, the last two aligning with a role in implantation (Fig. 6a and Supplementary Table 5). As JAK-STAT signalling was also implicated in our GSEA analysis for TE specification (Fig. 3a), we next examined its impact in the TE sublineages. We found that despite a maintenance in the total number of TE cells, the number of NR2F2+ cells was significantly reduced by JAK inhibition (10 µM AZD1480 from E4.5 to E6.5; Fig. 6b–d), suggesting that JAK-STAT plays a role in TE maturation before implantation.

Considering that the opposite lineages initiate attachment in humans and guinea pigs, we conducted a similar DEG and KEGG pathway enrichment analysis in human embryos (Supplementary Fig. 9a). A comparison between human and guinea pig revealed that 25 of 29 overlapping DEGs showed opposite expression in mural and polar TE between the two species (Fig. 5c). A broader analysis found that 360 of the 615 identified mural–polar DEGs (either in human or guinea pig) displayed opposite expression patterns between the two species (Supplementary Fig. 9b). Functional enrichment analysis for those genes underscored significant conservation related to fatty acid metabolism, insulin resistance, focal adhesion and glutathione metabolism, which may be linked to embryo positioning (Supplementary Fig. 9c). We also compared the DEGs identified from mouse mural and polar TE cells to human and guinea pig, but only identified minimal overlap among the TE sublineages (Supplementary Table 5)^55^.

### Role NR2F2 and retinoic acid in embryo apposition

**Extended Data Fig. 8:**
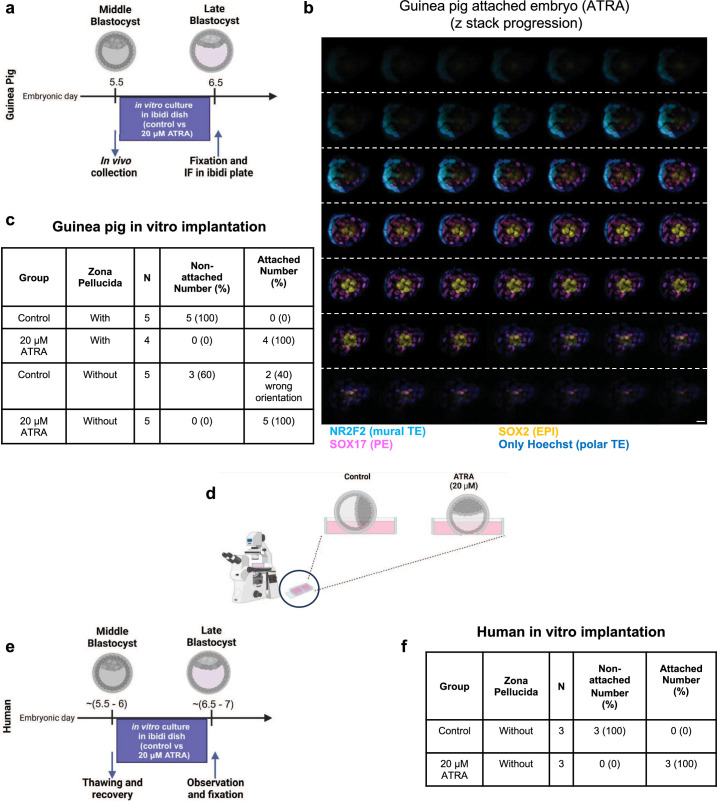
Impact of retinoic acid on embryo apposition. **a**, Schematic of ATRA treatment protocol in guinea pigs embryos. **b**, Representative immunofluorescence image showing a z-stack progression of an attached guinea pig embryo cultured on an ibidi plate with ATRA (20 µM) displaying SOX2 (yellow), SOX17 (magenta), NR2F2 (cyan) and Hoechst nuclear staining (blue) from bottom to top. **c**, Summary table of in vitro attachment success rates with guinea pig embryos. **d**, Schematic representation of in vitro attachment of guinea pig embryos. **e**, Schematic of ATRA treatment protocol in human embryos. **f**, Summary table of in vitro attachment success rates with human embryos. Schematic created in BioRender.com. Scale bars: 20 µm. Source data

During implantation, the mammalian endometrium produces retinoids, which are critical for initiating signalling pathways and key transcription factors that regulate embryonic development, such as those involved in TE and cytotrophoblast (CTB) differentiation^56^. Furthermore, the transcription factor TFAP2A (also known as activator protein 2α or AP-2α) regulates villous CTB differentiation, and its expression is induced by retinoic acid. NR2F2 is also known to promote CTB differentiation by activating TFAP2A, a process further potentiated by RARα and RXRα^56^. As such, we speculated that the conserved localization of NR2F2 at the implantation pole of the guinea pig and human embryo may serve as a sensor for apposition or implantation. Importantly, in the human preimplantation embryo, *RARα*, *β* and *γ* are minimally expressed, whereas *RXRα* and *β* are enriched in the TE^25,30^. We first analysed the protein expression and co-localization of RXRα and NR2F2 in guinea pig embryos and extended the analysis to human (Fig. 6e,f) and mouse embryos (Fig. 6g,h). We observed a progressive increase in the co-localization of NR2F2 and RXRα from 60% to 100% during the mid to late blastocyst transition in guinea pig embryos (Fig. 6e); however, in human embryos, despite the enriched expression of NR2F2 and RXRα in the polar TE (implantation pole), RXRα is still lowly expressed in the mural TE (Fig. 6f). In the mouse, neither Nr2f2 nor Rxrα are expressed from early blastocyst to late blastocyst (Fig. 6g,h), although the *Rxrα* transcript is found in earlier stages and is progressively downregulated towards the blastocyst stage (E4.5) (Fig. 6i). Our in vitro implantation assay with guinea pig and human embryos suggests that retinoic acid promotes proper embryo orientation and apposition, and that this may be mediated by NR2F2 and/or possibly via retinoic-acid receptors (Extended Data Fig. 8). The precise mechanism(s) underlying NR2F2, and/or retinoic acid receptors or hormone nuclear receptors, in driving apposition requires further mechanistic studies.

### Cross-species comparison

**Fig. 7:**
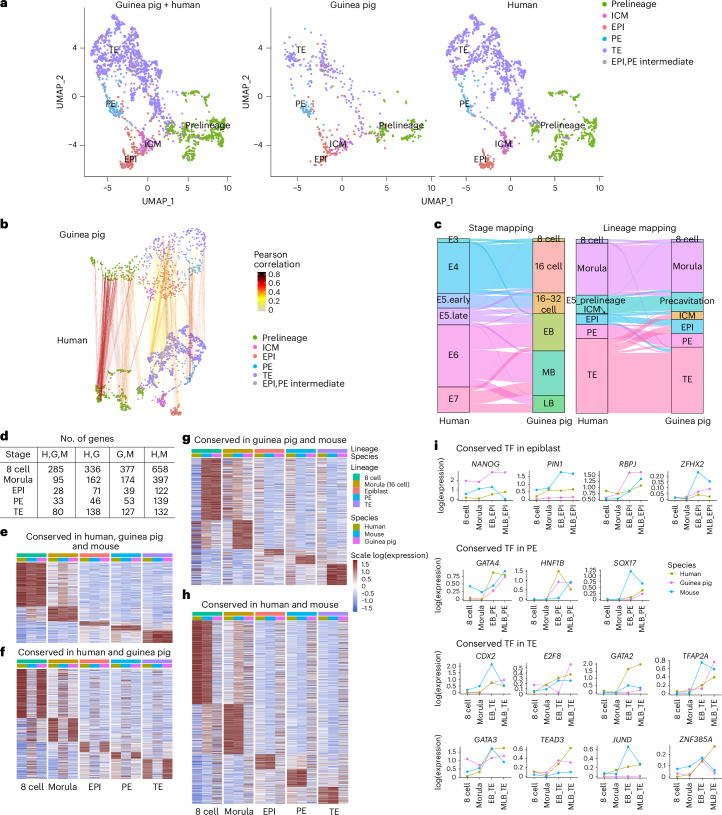
Cross-species analysis. **a**, Integration of preimplantation scRNA data from 1,381 human and 541 guinea pig cells (derived from 98 human and 42 guinea pig embryos, respectively). **b**, Subspace projection illustrating the linkage between MNN pairs in human and guinea pig. Line colours indicate Pearson correlation coefficients. **c**, Alluvial plot comparing guinea pig developmental stages and lineages with human embryonic days and lineages among MNN pairs. **d**, Number of genes categorized in different groups. **e**–**h**, Heatmaps representing scaled pseudo-bulk expression of conserved genes within a lineage among species: human, guinea pig and mouse (**e**); human and guinea pig but not mouse (**f**); guinea pig and mouse but not human (**g**); human and mouse but not guinea pig (**h**). **i**, Line plot displaying the pseudo-bulk expression of selected transcription factors stratified by stages and lineages in human, guinea pig and mouse. Different species are represented by different colours. Source data

**Extended Data Fig. 9:**
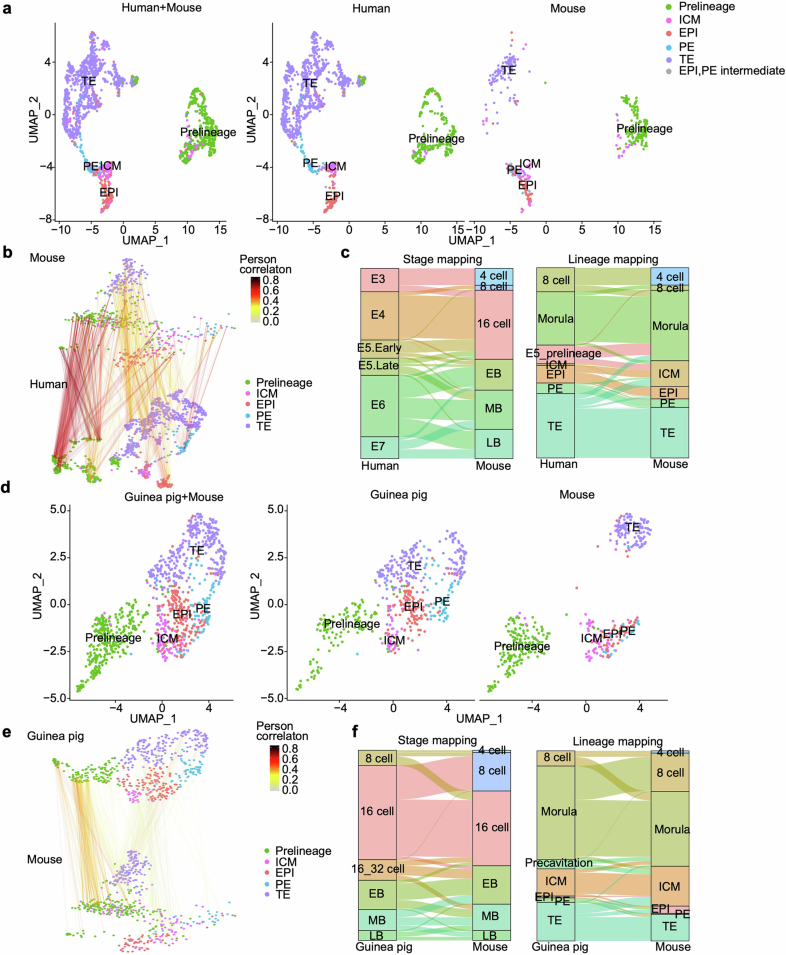
Cross-species integration with mouse datasets. **a**, Integration of preimplantation single-cell RNA data from 1381 human and mouse 423 cells (98 human and 74 mouse embryos, respectively). **b**, Subspace projection illustrating the linkage between mutual nearest neighbour pairs in human and mouse. Line colours indicate Pearson correlation coefficients. **c**, Alluvial plot comparing human developmental stages and lineages with mouse embryonic days and lineages among mutual nearest neighbour pairs. **d**, Integration of preimplantation single-cell RNA data from 541 guinea pig and 423 mouse cells (42 guinea pig and 74 mouse embryos, respectively). **e**, Subspace projection illustrating the linkage between mutual nearest neighbour pairs in guinea pig and mouse. Line colours indicate Pearson correlation coefficients. **f**, Alluvial plot comparing guinea pig developmental stages and lineages with mouse embryonic days and lineages among mutual nearest neighbour pairs. Source data

To compare between guinea pig and human embryos, we used canonical correlation analysis (CCA) anchors to identify conserved cell types^57^. By integrating the guinea pig transcriptome with public human embryo scRNA-seq datasets^30,58^, we visualized their alignment in UMAP space (Fig. 7a). Prelineage, ICM, EPI, PE and TE cells aligned consistently across species (Fig. 7a). A mutual nearest neighbour (MNN) search confirmed developmental stage equivalence between human and guinea pig preimplantation embryos (Fig. 7b,c), consistent with the immunostaining results (Fig. 7b,c). We also included mouse preimplantation embryo datasets^33,39^ for pairwise comparisons (Extended Data Fig. 9) among human, mouse and guinea pig. Human and mouse lineages showed generally good alignment (Extended Data Fig. 9a–c), with some biological differences being highlighted in terms of developmental time, such as the human E3 corresponding to the mouse ‘4 cell’ stage, and human E4 and early E5 corresponding to the mouse ‘16 cell’ stage. In contrast, although guinea pig and mouse lineages were generally aligned, the correlation was lower compared to human–mouse or human–guinea pig (Extended Data Fig. 9d–f), with stage misalignments in the prelineage (Extended Data Fig. 9f).

Next, we systematically evaluated the similarities and differences among the gene expression profiles in mouse, human and guinea pig preimplantation embryos (Fig. 7d–i). To enrich our cross-species analysis, an additional preimplantation mouse dataset with a clear gene expression signature and lineage information was included^40^. We identified conserved genes for each lineage across species (Supplementary Table 6). Overall, 521 genes were conserved among the mouse, human and guinea pig lineages (Fig. 7d,e). Among them, transcription factors were further interrogated based on both lineage and developmental time (Fig. 7i). Cross-species analysis showed a conserved peak expression in the guinea pig and human, but not in mouse from mid to late blastocyst of *NANOG*/*Nanog*, *SOX17*/*Sox17* and *TFAP2A*/*Tfap2a*. Furthermore, *CDX2*/*Cdx2* was expressed in the mouse but absent in the guinea pig and human at the 16-cell stage. These discrepancies underscore the need to consider both lineage and developmental time when examining regulatory factors in preimplantation development (Fig. 7i). We next wanted to examine genes that are conserved in only two of the three species in different combinations. In total, 753 genes were conserved between the human and guinea pig but with discrepancy (either absent or expressed in a different lineage) in the mouse (Fig. 7f and Supplementary Table 6). In total, 770 were noted to be only conserved in the mouse and guinea pig, including, *ELF5* and *EOMES*, previously identified as TE genes exclusively enriched in mouse TE and absent in human TE^30,59^. Finally, a total of 1,448 genes were only conserved between the human and mouse (Fig. 7h).

Taking a comparative biology approach to obtain a more comprehensive multi-species comparison, we checked the expression of selected well-known lineage markers for the human, marmoset, cynomolgus, mouse and guinea pig (Supplementary Fig. 10). Generally, primates, non-human primates and rodents demonstrate a conserved gene expression, but with some differences in the developmental dynamics of their respective expression (Supplementary Fig. 10). It is important to note that the absence of a gene in the guinea pig may be attributed to incomplete genome assembly rather than biological absence. Consequently, there is a possibility that we might be underestimating the transcriptional similarities between guinea pigs and other species.

## Discussion

**Extended Data Fig. 10:**
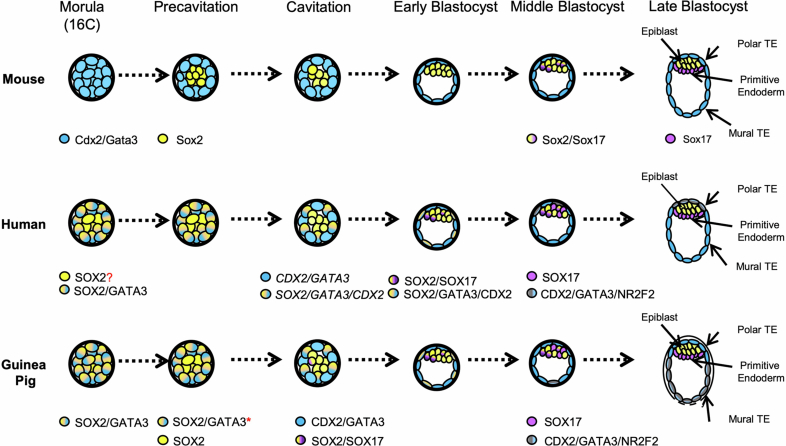
Schematic representation of fate specification in the mouse, human and guinea pig preimplantation embryo. Cells are coloured according to the expression of key transcription factors examined in this study that represent the three main lineages: Sox2/SOX2 (ICM/EPI), Cdx2/CDX2 and Gata3/GATA3 (TE), and Sox17/SOX17 (PE), Nr2f2/NR2F2 (implantation pole of TE). For mouse and guinea pig, marker expression is based on our immunofluorescence analysis. For the human, marker expression is reconstructed based on different sources (cited within the text) and this study. For human ‘cavitation’ and ‘precavitation’, marker expression is based on scRNA-seq data since immunofluorescence images are not available. *Note: Here we see the expression of CDX2 beginning to emerge in a portion of cells, albeit at very low levels and it remains co-expressed with SOX2, suggesting a poising prior to bona fide CDX2 expression. Whether this also occurs in the human embryo remains to be determined. Source data

In this study, we delve into the intricacies of guinea pig blastocyst development, aiming to unveil key molecular features governing early lineage segregation, pluripotency and X inactivation. By comparing our findings with established data from human and mouse embryos, we determined both conserved and divergent features in the early stages of mammalian development (Figs. 3f and 4h and Extended Data Fig. 10). We combined a comprehensive profiling of single-cell transcriptome and protein expression throughout preimplantation as well as functional analysis of signalling pathways, offering insights into the regulatory events shaping the specification of embryonic and extraembryonic lineages in guinea pig embryos and contributing to a broader understanding of the molecular landscape in early mammalian embryogenesis. We have summarized some key features of preimplantation development in the guinea pig, human and mouse, and include additional cross-species comparisons with rabbit and rat in Supplementary Table 7.

We have determined that aPKC and the evolutionarily conserved Hippo signalling are also involved in blastocyst formation and the establishment of the guinea pig TE, respectively. Similar to what has previously been reported in human, mouse, rat and bovine, we also observe that not all the downstream factors are conserved in their expression dynamics. The role of LATS1/2 in preventing ectopic expression of TE in the ICM of the guinea pig embryo appears similar to that observed in the human, where a decrease but not complete abolition of SOX2 in the ICM occurred^3^. These downstream differences may be linked to the conserved timing of lineage specification observed between guinea pigs and humans, which differs from mice, or perhaps a difference in SOX2 regulation and/or turnover. Furthermore, the timing of TE commitment occurs earlier in the mouse than in humans^33,60^. When lineage commitment occurs in the guinea pig remains to be determined; however, given the striking similarities, we speculate that it will occur post-implantation, as observed in humans^60^.

Moreover, the MEK-ERK signalling pathway appears to have a similar role in guinea pig as in human PE formation. In mouse^49^, bovine^51^ and rabbit^50^, inhibition of MEK completely abolishes SOX17 expression at low dose, whereas in guinea pig, human^48^, marmoset^34^ and pig^52^ embryos, this dose has a relatively modest impact. Notably, substantial reduction in SOX17 expression requires elevated concentrations of MEK inhibition in guinea pig and porcine embryos, which leads to unwanted toxicity effects^52^. The discrepancy in the effect of the MEK inhibitor among different species and the lack of a clear expansion in EPI cells following MEK inhibition in human, bovine, rabbit and guinea pig suggests a lack of a compensatory interplay between cells of the ICM (EPI/PE) as observed in the mouse, or that alternative pathways contribute to PE formation in these species. Two recent studies using human embryos report the interconversion of PE to EPI cells following inhibition of FGFR (PD173074) or ERK (ulixertinib), suggesting that FGF governs lineage segregation in humans, as in mice^61,62^. As the MEK inhibitor alone in human embryos does not achieve this interplay of lineages, we wonder whether the phenotype observed is a result of FGF signalling interacting with additional pathways to canonical MEK-ERK. ERK1/2 is known to crosstalk with different pathways, including JAK-STAT and PI3K-Akt^42,63^, which may explain why MEK inhibition alone cannot induce the switch between these lineages in human and guinea pig embryos.

In guinea pig embryos, implantation is orchestrated by the mural TE^64^, in contrast to humans, where implantation is initiated by the polar TE, adjacent to the EPI. Successful implantation is a major barrier to establishing a viable pregnancy. We have now identified a gene signature for polar and mural TE in the guinea pig, and established a role for JAK-STAT signalling in TE maturation. Furthermore, a cross-species comparison with the human revealed similar gene signatures for the implantation pole. In the guinea pig, in contrast to the human, it is the mural cells that proliferate and aid the embryo in undergoing interstitial implantation. As such, it is not surprising that genes involved predominantly in apposition/implantation would be expressed in the mural TE of the guinea pig, as opposed to the polar as observed in the human. Of particular interest, we noted high expression of NR2F2 and RXRa marking the implantation poles of the human and guinea pig, but not the mouse. These nuclear receptors, and perhaps the additional conserved genes identified in the implantation poles, may be particularly important for apposition, implantation and TE differentiation.

As with any modelling system, it is critical to acknowledge both the similarities and disparities inherent in what is being modelled. Throughout this manuscript we have underscored numerous advantages of employing the guinea pig as a model for investigating early human embryogenesis and the lasting health impacts on offspring due to exposures or ‘insults’. We now address some important limitations of this modelling system. First, the current guinea pig genome assembly, established 15 years ago, is outdated and accessible only at the scaffold level, resulting in substantial genomic gaps. Moreover, both Ensembl and RefSeq encounter issues such as inaccurate transcription boundaries, missing isoforms and unannotated genes. We have taken precautions to ensure accurate gene transcription regions via genome-guided transcriptome assembly, but the incomplete genome assembly, coupled with insufficient sequence depth and coverage biases in scRNA-seq data, may still present challenges. Similar gene annotation challenges are present with other species, including the rabbit genome, which was last updated in 2009 (rabbit genome OryCun2.0)^65^. Undoubtedly, there is a need for the establishment of a more comprehensive guinea pig genome assembly reference. This advancement will facilitate comprehensive multi-omic studies and cross-species analyses, providing a deeper understanding of the evolutionarily conserved and divergent mechanisms underlying embryogenesis. In addition, current suboptimal superovulation protocols and small litter sizes (typically two or three embryos) pose challenges. The limited embryo yield constrains the number of manipulations that can be conducted simultaneously, thereby reducing throughput and increasing experimental costs. Furthermore, culturing conditions for the very early stages (zygote to compaction) remain to be optimized, thus limiting in vitro manipulation experiments during this window. Of note, the culturing conditions utilized in our study were post-compaction and resulted in embryos that developed in accordance with those obtained in vivo. However, this does not negate the possibility that minute differences may occur in the molecular circuitries with different culturing conditions. Our laboratory is actively working to optimize these aspects, aiming to enhance the flexibility and utility of the guinea pig model for studies on early embryogenesis. We have also developed an online ShinyApp for exploring guinea pig gene expression (https://petropoulos-lanner-labs.clintec.ki.se/shinys/app/ShinyGpigPreImpEM)^66^, which we believe will serve as a valuable resource for the scientific community.

Overall, our study demonstrates conserved features between guinea pig and human preimplantation development. Furthermore, the guinea pig provides an in vivo system that is not under the same ethical and legal constraints as those for human embryos or stem cell-based human embryo models, which allows for genetic modification as well as the assessment of longer-term phenotype(s) and offspring outcomes. Finally, retrieval of early post-implantation embryos is feasible and, given the striking similarities with implantation, amniogenesis and placentation in the human, the guinea pig can also serve as an excellent model to enhance our understanding of post-implantation development. We now propose that the guinea pig presents a robust small-animal model that can be utilized to enhance our understanding of both comparative biology and human embryogenesis.

## Methods

### Ethical statement

All procedures involving animals were approved by the Comité Institutionnel de Protection des Animaux (CIPA; IP19022SPci). Human embryos were obtained from the Clinique OVO with ethical approval from the regional ethics board at the Centre de Recherche du Centre Hospitalier de l’Université de Montréal (CRCHUM) and Université de Montréal (CERSES-20-107-R and 20.126) for the research purpose to understand fundamental aspects of human preimplantation development. Use of the human embryos in this study was in compliance with guidelines from the Ministère de la Santé et des Services Sociaux, the International Stem Cell Society of Research (ISSCR), the Canadian Institutes of Health Research Stem Cell Oversight Committee (SCOC) and regional ethics boards. Informed written consent was obtained from patient(s) who donated supernumerary embryos following in vitro fertilization (IVF) treatment. Donors were aware of the research purpose and embryos were received de-identified. No financial compensation was offered for donations. Embryos were not created for research purposes nor were they genetically manipulated. Embryos were thawed following vitrification and cultured until E7, at which point they were fixed for imaging. As such, they were well within the 14-day limit.

### In vivo collection of guinea pig embryos

Hartley guinea pigs (Charles River Labs) were housed in the Animal Care Facility at the CRCHUM, maintained at 21 °C with 30–70% humidity under a 12-h light cycle, and fed with Teklad Global Guinea Pig Diet 2040 pellets, water and hay ad libitum. For each stage, embryos were retrieved from at least three separate female guinea pigs (~500 g, 2–3 months of age) between days 3.5 and 5.75 after natural mating. We have previously described the detailed procedure of euthanasia, dissection and flushing^68^. Briefly, embryos were flushed from the oviduct (E3.5) or uterine horns (E4–E5.75) using 5–10 ml of warm M2 medium (MR-015-D, Sigma Aldrich) supplemented with 1% penicillin–streptomycin (15130122, Life Technologies), washed and briefly kept in M2 drops covered with Ovoil (10029, Vitrolife) in an incubator at 5% O_2_ 5% CO_2_ at 37.5 °C, just before being fixed in paraformaldehyde (PFA) 4% for 15 min or single-cell picking. E6 embryos were collected at E5.75 and incubated in similar conditions for 6 h to reach the desirable embryonic day, as implantation occurs around E6.

### In vitro culture of guinea pig embryos for functional analysis

Optimal culturing conditions for guinea pig preimplantation embryos from the zygote to precompaction stage remain to be determined. We experimented with media previously reported to be used with the guinea pig^10^, in addition to what is commonly used in different species, including M2 and KSOM (mouse)^69^, RDH (rabbit)^50^, mR1ECM (rat)^3^, GTL^43^ and Global medium^3^ (human) and N2B27 (mouse^70^, human^60^, bovine^51^, ovine^71^, porcine^52^ and blastoids^72^). We found that embryos do not progress in vitro in any of the media tested, before compaction (1C to non-compacted 16C) (Supplementary Table 8). We were able to successfully culture embryos from the compacted 16C stage until late/expanded blastocyst with only N2B27. N2B27 supports the development of the blastocyst and establishment of the EPI/PE/TE lineages marked by the expression of SOX2+, SOX17+ and CDX2+/GATA3+, respectively. We thus utilized N2B27 culture medium and preimplantation guinea pig embryos were cultured in vitro, as previously described^68^, to perform the functional analysis studies. Briefly, culture dishes were prepared by placing 50-μl drops of N2B27 culture medium on 35-mm plastic dishes, subsequently covered with embryo-tested light mineral oil (Ovoil or Embryomax) and equilibrated at 38.5 °C and 5% CO_2_ for a minimum of 30 min before embryo culture. Embryos were then collected in vivo by flushing with M2 manipulation medium from E4 to E4.5 and then passed through multiple wash drops of N2B27 before culturing them together in one drop of N2B27 medium with DMSO or inhibitors (further details are provided in the 'Functional analysis with small molecules' section)^71^.

### Human and mouse embryo culture

E5 vitrified human embryos were thawed using a vitrification thaw kit (Irvine Scientific; 90137-SO) according to the manufacturers’ recommendations and cultured in droplets of pre-conditioned GTL medium (Vitrolife; 10145) in 60-mm IVF dishes (Corning C353802 from Thermo Scientific; 150260). Human cultures were covered with mineral oil (Ovoil, Vitrolife; 10029).

Mouse embryos at each stage were retrieved from at least three separate female C57BL/6 mice (Charles River, 6–8 weeks of age) following superovulation and mating as previously described^69^, and cultured in droplets of pre-conditioned KSOM (Sigma Aldrich, MR-121-D) covered with mineral oil (Embryomax, Millipore; ES-005-C) in 35-mm IVF dishes (Nunc, Thermo Scientific; 150255).

Preimplantation embryos were incubated at 37 °C under hypoxic (5% CO_2_ and 5% O_2_) or normoxic (5% CO_2_ and 21% O_2_) conditions for human and mouse embryos, respectively.

### Functional analysis with small molecules

All guinea pig embryos utilized for the functional experiments were flushed around E4–4.5 (post-compaction) and cultured in N2B27 medium together with the respective small molecule, as indicated below. The exception was inhibition of PI3K-Akt signalling as N2B27 has insulin in its composition. We thus formulated a base medium consisting of 1:1 DMEM/F12 and KSOM and added components according to Supplementary Table 9. Guinea pig compacted morulas developed to blastocysts at a similar rate as when cultured with N2B27.

To study Hippo signalling, the LATS inhibitor (TRULI, Enamine; Z730688380)^3,44^ was initially dissolved in DMSO to create a stock concentration of 100 mM. This stock solution was then diluted to the necessary concentrations using pre-equilibrated embryo culture media. The optimal concentration of TRULI, a LATS1/2 inhibitor, used for the mouse was determined by a dose–response analysis (Supplementary Fig. 5) and previous work^3^. The optimal concentration of TRULI for guinea pigs was determined to be in the range of 5–10 µM, considering the impact of the inhibitor on embryo viability and phenotypic outcomes, particularly in terms of active YAP, GATA3 and SOX2 expression at E5.5 (Extended Data Fig. 6a–d).

To investigate whether the timing of exposure to TRULI (16 cell versus 8 cell in the guinea pig and mouse, respectively) influenced the observed disparities in the effect of Hippo signalling inactivation on Sox2 in inner cells, we conducted an additional experiment. Recognizing that Sox2 expression in mice begins at the 16-cell compacted morula stage, we then treated mouse embryos from the 16-cell stage to the late blastocyst (Supplementary Fig. 6d,e). In the mouse, Lats inhibition led to a significant ectopic nuclear localization of aYAP and GATA3 in inner cells. Notably, the number of outer cells expressing aYAP and GATA3 at late blastocyst in treated embryos did not differ from controls, in contrast to the shorter exposure (eight cell to early blastocyst). This suggests that the embryo regulates the number of cells in the TE just before implantation. Upon Lats inhibition, the number of Sox2-expressing cells in inner cells was not completely abolished (5/7 embryos had no Sox2) by the late blastocyst stage (Supplementary Fig. 6d,e), implying that a residual number of cells expressing Sox2 can be found in the embryo if the treatment starts later.

As an evolutionarily conserved molecular cascade initiates the TE program in human, cow, rat and mouse embryos at the morula stage^3,21^, and aPKC is a component of the apical domain responsible for establishing cell polarity and functioning upstream of the Hippo signalling pathway to initiate this program^3,21^, we sought to determine whether aPKC is functionally conserved in guinea pig embryos. We conducted a side-by-side comparison with mouse embryos using the small-molecule inhibitor Gö6983 (Tocris)^60,73^. A dose–response experiment was performed, culturing embryos from the compacted morula stage to the blastocyst stage, with doses ranging from 2 to 5 µM for mouse and 2.5 to 10 µM for guinea pig embryos (Supplementary Fig. 7). Gö6983 was dissolved in DMSO to create a 50 mM stock solution, and diluted to the required concentrations in pre-equilibrated embryo culture media. Control embryos were cultured in media containing 0.1% DMSO, equivalent to the concentration used for inhibitor treatments.

Although guinea pig and mouse embryos treated with optimal doses of 3.5 µM and 3.75 µM Gö6983, respectively, arrested at cavitation (Supplementary Fig. 7), in contrast to DMSO-treated control embryos, which formed expanded blastocysts, expression of the outer-cell markers aYAP/aYap and GATA3/Gata3 was maintained in both species. This outcome differs from a previous study reporting the abolition of YAP1 and GATA3 expression in mouse and human embryos treated with CRT0276121^21^, a potent PKC inhibitor that is currently not commercially available. To further investigate these differences, an additional dose–response experiment was performed using an alternative PKC inhibitor, CRT0066854, on mouse embryos. Embryos were treated with 5 and 7.5 µM of the inhibitor and cultured from the four-cell stage to late blastocyst at E4. However, results using CRT0066854 also failed to entirely replicate the phenotypic effects observed with CRT0276121 (Supplementary Fig. 7e–h), suggesting that aPKC inhibitors target downstream components differently across species and necessitating further studies to clarify the role of aPKC in the TE program of guinea pig embryos.

For JAK inhibitor AZD1480, we cultured guinea pig embryos from E4.5 to E6.5 using a concentration of either 5 or 10 µM or DMSO as a control group (Extended Data Fig. 7a–c), with the higher dose based on literature^52^. Although the total cell number decreased at 10 µM (Extended Data Fig. 7c, *P* < 0.05; Fig. 4c, *P* < 0.01), the DNA integrity (Hoechst staining) and overall embryo morphology, including blastocyst formation, were unaffected. This suggests that the reduced total cell number is not due to toxicity, but rather a direct effect of JAK-STAT pathway inhibition on cell lineages (Fig. 4b,c). We established an optimal concentration of 10 µM for the JAK inhibitor (Fig. 4b,c).

We conducted a comparative analysis examining the inhibition of the MEK-ERK pathway, known for its conserved role in PE formation across various species, including mice, rabbits, bovines, pigs and humans. Our dose–response for guinea pig embryos included 1, 2.5, 5 and 10 µM PD0325901, with the highest dose based on previous studies conducted on bovine and porcine embryos^51,52^ and control (DMSO), treating from the 16-cell stage until blastocyst. With 2.5, 5 and 10 µM PD302, we observed a decrease rather than an abolition of SOX17+ cells (Extended Data Fig. 7d,e), as previously reported in porcine and human PE^48,52^. However, using these concentrations also resulted in a decrease in the total cell number and blastocyst survival, suggesting a toxicity effect. For a parallel experiment in the mouse, we cultured embryos from E2.5 to E4.5 using 1 µM PD325901 or DMSO control (Supplementary Fig. 6f,g), as previously described^49^.

Finally, for the PI3K inhibitor, we cultured guinea pig embryos from E4.5 to E6.5 and performed a dose–response experiment using 5, 7.5 and 10 µM concentrations of the PI3Kα/δ/β inhibitor LY294002. In parallel, we performed a dose–response analysis of PI3Kα/δ/β inhibitor LY294002 (5 and 10 µM) and control in mouse embryos, culturing embryos from E2.5 to E4.5 (Supplementary Fig. 8). As we did not observe any significant impact in guinea pig nor in mouse when inhibition was performed for 48 h, we further explored the effect of inhibiting PI3K only for 24 h using the 7.5 µM dose in guinea pig embryos (Fig. 4f,g), speculating that a longer culture would enable activation of compensatory signalling pathways. We added this timepoint of sampling for guinea pigs as we noticed that for porcine embryos, the PI3K inhibitor has an effect when embryos are cultured for a short time (morula to mid blastocyst)^52^.

All embryos used to assess the function of signalling pathways were analysed by immunofluorescence to determine the number of cells expressing lineage-specific markers. Unless otherwise stated, the maximum projection of images is presented for each marker, and a final merge image is included to show co-expression of those markers analysed simultaneously. For TRULI and PKC functional analysis (Extended Data Fig. 6, Fig. 3 and Supplementary Fig. 7, respectively, for guinea pigs and Supplementary Figs. 5–7 for mouse), the scatter plots show the total number of cells positive for each marker (SOX2, aYAP and GATA3). We also discriminated by localization in the TRULI experiments (inner versus outer cells). For the MEK-ERK and PI3K-Akt functional analysis, immunofluorescence was performed using antibodies against SOX2 (EPI), SOX17 (PE) and CDX2 (TE), and the scatter plots represent the total number of SOX2+, SOX17+ and CDX2+ cells (Fig. 4, Extended Data Fig. 7 and Supplementary Fig. 8 for guinea pigs and Supplementary Figs. 6 and 7 for mouse). Finally, for JAK-STAT functional analysis, guinea pig embryos were first co-stained with SOX2 (EPI), SOX17 (PE) and NR2F2 (mural TE) and then recovered to stain with CDX2 (mural and polar TE). Scatter plots represent the total number of cells of each marker (SOX2, SOX17 and CDX2 in Fig. 4 and Extended Data Fig. 7; SOX2 and NR2F2 in Fig. 7 for guinea pigs). Statistics and graphs were generated using GraphPad software version 9.2.0. Schematics of the treatment protocols for each small molecule were generated using BioRender.

### Immunofluorescence

Immunofluorescence was performed on guinea pig embryos as we previously described^68^. Briefly, embryos were fixed in 4% PFA in phosphate buffered saline (PBS) at room temperature (r.t.) for 15 min, permeabilized in PBS 0.25% Triton X-100 at r.t. for 10 min, and then blocked in PBS with 3% bovine serum albumin (BSA) for 2 h at r.t. Samples were incubated with primary antibody overnight (O/N) at 4 °C in blocking solution and with secondary antibodies for 2 h at r.t. in blocking solution. Washes were performed after primary and secondary antibody incubations (4 × 5 min in PBS). Hoechst (33342, Invitrogen) was used for nuclear staining. All incubations and washes were carried out in a clean well using Nunc 72-well mini trays (CA62409-296, VWR) with 17 µl of each solution. Embryos were mounted in PBS and placed between two coverslips (1.5 thickness) using SecureSeal spacers (Grace Bio-Labs). The antibodies used are listed in Supplementary Table 10. Of note, in the guinea pig, we were unable to examine the expression of aPKC and adhesion molecules as we were unable to find suitable antibodies for aPKC, Pard6, E-cadherin and β-catenin (Supplementary Table 10).

Images were acquired using an Olympus FV1000MPE confocal microscope equipped with an XLUM Plan FL N ×20/1.00 water objective. For excitation, 405-nm (solid-state), 488-nm (argon laser), 543-nm (solid-state) and 635-nm (solid-state) lasers were used for 4′,6-diamidino-2-phenylindole (DAPI), Alexa Fluor (AF) 488, AF594 and AF647, respectively. For detection, photomultiplier tube (PMT) detectors were set as follows: a first SDM490 was positioned in front of the first PMT associated with a BA 430-470 for DAPI detection, then an SDM560 was positioned in front of the second PMT associated with a BA 535-565 for AF488 detection, a SDM640 was positioned in front of the third PMT associated with a BA 560-660 for AF594 detection, and finally a mirror was positioned in front of the last detector with a BA 655-755 for AF647 detection. All images were acquired sequentially (frame mode) as follows: AF488 and AF647 simultaneously first, AF594 second, and DAPI at the end of the sequence. Images were acquired in a 512 × 512 or 800 × 800 pixel format with zoom at 4 μs pixel^−1^ speed with a line Kalmann of 3. *Z*-stacks were acquired with a 1-µm step size. Images were acquired using the Olympus FluoView software (v4.2.3.6, Olympus). For each embryo, *z*-stacks were analysed using Fiji software version 1.53c, allowing virtual labelling based on DNA staining (Hoechst staining) for all individual cell nuclei and counting the total number of cells. Using this labelling to identify individual cells, each cell in every embryo was then categorized according to relevant phenotypic criteria, without knowledge of the embryo treatment (blind counting). Phenotypic categories included marker expression (for example, SOX2 or CDX2 positive or negative), protein localization (nuclear or cytoplasmic) and cell position, with cells in contact with the external environment classified as ‘outside’, and cells surrounded by other cells considered ‘inside’ cells, as in ref. ^23^. For all analyses (with the exception of H3K27me3 measures), the background was subtracted, and a Gaussian filter was applied. To count cells, we created masks for each channel and used the tool to analyse particles to count the number of cells per embryo as previously reported^51^. We visually inspected the masks to account for any error in counting generated by a wrong segmentation of cells. The scatter plots in Fig. 1 were created accounting for the number of cells per embryo that only express each lineage marker (SOX2: EPI, SOX17 or GATA6 for PE and CDX2 or GATA3 for TE) and the number of cells that co-expressed different combinations of those markers. For characterization of the TE sublineages (Fig. 5), we determined the relative contribution of the TE sublineages in the guinea pig by quantifying the number of NR2F2+/GATA3+ (mural) and GATA3+ only cells (polar) (Fig. 5e,f). In contrast to the human, where the polar sublineage constitutes the smaller proportion of TE cells adjacent to the ICM^19,30^, the number of cells of each sublineage did not significantly differ in the guinea pig TE (Fig. 5e,f). Statistics and graphs were created in GraphPad software version 9.2.0.

### Role of retinoic acid in guinea pig embryo apposition

We explored the role of retinoic acid during embryo implantation by culturing guinea pig and human embryos for 24 h, from E5.5 to E6.5 in guinea pigs or thawing E5 human embryos and culturing in modified N2B27 medium without retinoic acid (Supplementary Table 11 describes its composition) using ibidi µ-treated plates^74,75^. The experimental group was cultured with 20 µM ATRA (all-*trans* retinoic acid)^56^ and compared to a control group, which had no retinoic acid in the culture medium. RXRs and RARs are known to be receptive to 9-*cis* retinoic acid, whereas only RARs are receptive to ATRA^76^. As such, given the lack of RAR receptor expression and our interest in looking at the interaction between retinoic acid and NR2F2, we chose to stimulate with ATRA. The guinea pig embryos were cultured with and without zona pellucida, as guinea pig embryos are believed to hatch upon implantation and not prior to. For human embryos, all zona pellucida were removed. After 24 h in culture at 38.5 °C and 5% CO_2_ for guinea pigs and at 37 °C and 5% O_2_ and 5% CO_2_ for humans, the embryos were inspected under the microscope using a Zeiss AxioObserver Z1 Yokogawa CSU-X1 spinning disk confocal inverted microscope, and the attachment status recorded. In the presence of ATRA, the guinea pig consistently oriented and attached at their respective implantation pole (mural TE) (Extended Data Fig. 8b,c). In contrast, embryos cultured under control conditions either did not attach at the implantation pole (embryos without zona pellucida) or completely failed to attach (embryos with zona pellucida) (Extended Data Fig. 8c). Attached guinea pig embryos were fixed, and immunofluorescence analysis was performed in the ibidi plate as described above for other embryos. SOX2 (EPI), SOX17 (PE), NR2F2 (mural TE) and Hoechst were analysed to establish how the embryo attached in vitro. Similarly, when cultured with ATRA, all human embryos were attached at the implantation pole (Extended Data Fig. 8f,g). Unfortunately, human embryos were lost during the process of immunostaining.

### scRNA-seq library preparation

Female guinea pigs were flushed between E3.5 and E5.75 (*n* = 4 at 8C, *n* = 7 at 16C, *n* = 4 at 16–32C, *n* = 6 at EB, *n* = 16 at MB and *n* = 14 at LB), and the embryos were briefly placed in M2 drops under mineral oil for downstream processing. The zona pellucida was removed using Tyrode’s solution, and embryos were dissociated into single cells with TrypLE Express (12604-013, Gibco, Life Technologies) collected using fine glass capillaries, as we previously described^69^. Libraries were prepared using Smart-Seq2 as previously described^30,33,77^. The quantity and quality of the cDNA libraries were assessed using an Agilent 2100 BioAnalyzer (Agilent Technologies). Approximately 1 ng of cDNA per cell was transformed into a single-cell library using the Nextera XT DNA Library prep kit (Illumina FC-131-1096) and following the manufacturer’s instructions with slight modifications. In brief, 1 ng of cDNA (in a maximum of 5 µl of sample volume) underwent tagmentation with 2 µl of Tagment DNA buffer (TD) and 1 µl of Amplicon Tagment Mix (ATM) at 55 °C for 5 min. On completion of the program, the reaction was halted by adding 1 µl of 2% sodium dodecyl sulfate (SDS) and incubating for 5 min at r.t. Amplification was carried out using 3 µl of N-methyl-2-pyrrolidone (NMP) per sample and adding 1 µl of each dual-indexed (i7 and i5; Illumina) primer. Individual Nextera XT libraries were pooled and then purified using magnetic beads. Indexed libraries were combined for multiplexing (384 samples per lane). The sequencing was performed using the NovoSeq6000 S4 at 150 bp paired ends. Sample sizes were based on previous publications using scRNA-seq to determine lineage in preimplantation embryos^4,33^. The cell numbers were also assessed using the tool available at https://satijalab.org/howmanycells/, assuming seven cell types with a minimum fraction of 0.1 and at least 30 cells per cell type.

### RNA fluorescence in situ hybridization

In mouse embryonic stem cells (mESCs) and mouse preimplantation embryos, lncRNA *XIST* is a master regulator of XCI and acts *in cis* to silence the active X chromosome. *XIST* recruits the polycomb group complex PRC2 to the X chromosome, that subsequently catalyses the repressive epigenetic mark, and trimethylates histone 3 on lysine 27 (H3K27me3), which in turn leads to further silencing of the X chromosome^78^. In mouse preimplantation embryos and mESCs, a close relationship has been reported between *XIST* and the status of the X chromosome^31,79^. In contrast, an uncoupling between *XIST* activity and X-chromosome status occurs in human blastocysts and naïve human pluripotent stem cells (hPSCs)^30,80–82^, which we have previously reported to be associated with bi-allelic dampening. In human embryos, the transition to random XaXi is believed to have initiated around E8 but not fully resolved by E12^83^. Given these notable differences between the mouse and human, we wanted to examine the status of the X chromosome in the guinea pig preimplantation embryo. RNA fluorescence in situ hybridization (FISH) for the long noncoding RNA *Xist*/*XIST* was performed in mouse and human embryos, respectively, following our previously described protocol^84^. In brief, E3.5–3.75 mouse and E7 human embryos were fixed, placed on a silanized glass coverslip, and air-dried for ~2 min. Subsequently, embryos were permeabilized with pre-chilled (−20 °C) methanol (Sigma) for 10 min at −20 °C. After air-drying with methanol for 30 min at r.t., the embryos underwent heatshock and were hybridized for 2 h at 38.5 °C in a humidity chamber with a *XIST* Quasar 570 (125 nM; SMF-2038-1; BioSearch Technologies) in hybridization buffer. This buffer comprised RNase-free water, 2× saline sodium citrate (SSC), 10% wt/vol dextran sulfate (Sigma), 10% formamide (Thermo Fisher Scientific), 2 mg ml^−1^ *Escherichia coli* tRNA (Sigma), 2 mM ribonucleoside vanadyl complex (New England Biolabs) and 2 mg ml^−1^ BSA (Jackson ImmunoResearch). Following hybridization, samples were washed with 20% formamide in 2× SSC. Hoescht 33342 (1 µg ml^−1^; Thermo Fisher Scientific) was added to the wash buffer during the final wash. Samples were washed again, mounted with Prolong Diamond antifade (Thermo Fisher Scientific) and air-dried in the dark at r.t. for 24 h before imaging.

The resolution of the reads obtained from the X chromosome and Y chromosome as well as the exact sequences for *XIST* and *XACT* noncoding RNA, can provide further insights into the disconnect between H3K27me3, *XIST* and X-chromosome dosage compensation, which at this time is not feasible due to the incomplete guinea pig genome assembly.

### Imaging for FISH

Images were acquired using a Zeiss AxioObserver Z1 Yokogawa CSU-X1 spinning disk confocal inverted microscope. The microscope was equipped with a motorized stage, piezo objectives and an Evolve electron-multiplying charge-coupled device (512 × 512, 16 bits, 16-µm pixel size) monochrome camera (Photometrics). Image acquisition utilized an alphaPlan Apo ×100/1.46 oil DICIII (UV) M27 objective (Zeiss), resulting in a final pixel size of 133 nm. For DAPI excitation, a 405-nm (solid-state) laser was employed, coupled with a double-bandpass filter in emission (480/22 + LP530). Similarly, AF568 excitation utilized a 561-nm (solid-state) laser coupled with a double-bandpass filter in emission (460/30 + 590/30). *Z*-stacks were executed to capture multiple nuclei, with a 220-nm step size.

### H3K27me3 immunostaining

H3K27me3 is an epigenetic mark typically associated with silencing and is found to accumulate on the inactive X chromosome in mouse embryos^85^, and to localize to both active X chromosomes (XaXa) in naïve stem cells that exhibited dual X-chromosome activity^82,86^. Furthermore, in both the rabbit and cynomolgus monkey, an accumulation of H3K27me3 on the two *XIST*-expressing active X chromosomes has been shown^80,87^. To confirm the use of H3K27me3 as a proxy, we performed the immunofluorescence analysis as described above. Following image acquisition, we arbitrarily analysed three or four embryos per species. First, a number was randomly assigned to each of the cells within the embryo, creating a region of interest (ROI, delimiting each cell) set per embryo. To count the number of H3K27me3 foci per cell in each embryo, we then arbitrarily selected a subsample of 25–35 cells by drawing lots, and manually counted the number of foci per cell. We verified that, in the majority of cells, H3K27me3 expression mirrored that observed by *XIST* RNA FISH in the mouse and human (Extended Data Fig. 2a,b), providing evidence of H3K27me3 localization in human female blastocysts^88^, which also supports a disconnect or lag between H3K27me3 deposition in the human embryo, similar to what has been shown in naïve stem cells and in line with an incomplete XCI in the preimplantation embryo^30,79,86^. Male blastocysts, in contrast, lacked distinct foci staining of H3K27me3, but diffuse nuclear staining was observed (Extended Data Fig. 2c) for mouse, human and guinea pig embryos. We measured the nuclear H3K27me3 signal (Fiji 2.16.0 software) in three independent ROIs for the foci (females) versus the nuclear section with diffuse signal (autosomes) for each of the previously selected cells in female and male embryos (Extended Data Fig. 2e–g). Of note, when measuring the H3K27me3 signal intensity we did not remove the background.

### Sex determination of guinea pig embryos

After imaging H3K27me3, the guinea pig embryos were retrieved from the mounted slides, washed in PBS, and transferred with 2.5 µl of PBS to a tube containing 2.5 µl of extraction buffer (PicoPLEX WGA kit, final volume 5 µl). Whole DNA extraction and amplification from single guinea pig embryos were conducted using the PicoPLEX WGA kit following the manufacturer’s recommendations. After amplification, samples were quantified using Qubit, and the sex of the embryo was determined through end-point polymerase chain reaction (PCR) for guinea pig DYS and SRY genes as previously outlined^89^. DYS-F (GTGTTAATGGTGACAGCATCAGC) and DYS-R (GTGCTGTTGGATCTGAAGTGGAGG) were used for detecting the X chromosome, and (CCGAGACCAGAGACGCAAGATAGC) and (CACTGGTATCCCAGCAGCTTGC) were used as SRY-F and SRY-R, respectively, for detecting the Y chromosome. PCR reactions (25 µl) were carried out using DreamTaq Hot Start Green PCR mix solution (Thermo Fisher Scientific), with 0.4 µM of each dystrophin primer, 0.8 µM of each Sry primer, and up to 20 ng of genomic DNA. The PCR cycling steps were as follows: 95 °C for 3 min, followed by 36 cycles at 95 °C for 30 s, 58 °C for DYS and 62 °C for SRY for 30 s, and 72 °C for 30 s, with a final cycle at 72 °C for 5 min. PCR amplicons were analysed on 2% agarose gels and stained with SYBR Safe DNA gel stain (S33102, Invitrogen). The expected amplicon sizes were 212 base pairs (bp) and 88 bp for dystrophin and Sry gene amplification, respectively (Supplementary Fig. 11a,b). Ultraviolet gel images were obtained using the GelXDoc from BioRad with automatic exposure.

### Statistics and reproducibility

No statistical methods were used to predetermine sample sizes; instead, sample sizes used were similar to those reported in previous publications^3,21,30^. For functional analyses, the embryos were arbitrarily assigned to the control and treatments. The investigators were aware of group allocation during the experiments; however, cell counting and intensity measurements were performed in a blinded manner for each embryo using the embryo ID (composed of the year, mother ID and embryo number). Embryos were reassigned to their respective groups for graphical depiction. All statistical analyses (excluding those related to scRNA-seq analyses) were executed using GraphPad Prism 9.2.0. Detailed information regarding the number of cells or embryos analysed (*n*), the specific statistical tests employed and the corresponding *P* values are provided in each figure or its respective legend. Analysis of data acquired from counting cell numbers (presence/absence of a marker) were analysed using a nonparametric test (Mann–Whitney for a two-group comparison or Kruskal–Wallis for more than two groups), because the statistical variable defined as ‘number of cells per embryo’ does not follow a normal distribution^51^. The data generated by quantifying the intensity were analysed using a Kruskal–Wallis test because these data do not meet the assumption of homoscedasticity (*P* < 0.0001 in a Bartlett test). The data presented are typically expressed as mean ± s.e.m. unless otherwise stated, and each experiment was performed at least three times.

### Improving mapping for guinea pig scRNA-seq data

For the guinea pig scRNA-seq data, adapters and low-quality reads were removed using Trimgalore (https://github.com/FelixKrueger/TrimGalore) with default parameters. Subsequently, high-quality reads were aligned to the guinea pig Ensembl reference genome (Cavpor.3.0.v105, *Cavia porcellus*, obtained from the Ensembl website)^90^ using the HISAT2 aligner (v2.2.0)^91^ with default settings. Only uniquely mapped reads were retained for gene expression quantification. Raw read counts were tentatively quantified using featureCounts (v1.6.3)^92^ based on Ensembl reference annotation. However, taking this approach, we initially observed low gene expression for well-conserved lineage marker genes such as *SOX17* and *CDX2*, and no gene was annotated as *SOX2*, which we knew was inaccurate given the immunoflurescence stainings. When we then switched to RefSeq annotation (downloaded from USCS, CarPor3), we found no expression for *BMP2* and *SOX17*. Visualizing read coverage across the genome, we discovered that a large majority of sequencing reads were aligning to nearby regions and, as such, incorrectly not aligned to the gene location itself based on the incomplete Ensembl and RefSeq annotations (Supplementary Fig. 1). We therefore decided to update the guinea pig gene annotation using the following steps. Ensembl transcripts belonging to ‘lincRNA’, ‘processed_pseudogene’, ‘protein_coding’ and ‘pseudogenes’ were selected for the genome-guided assembly. The mapping bam files were used as input for Stringtie (v1.3.3b)^93^ for each individual sample. After the initial assembly, the transcripts were merged using ‘stringtie --merge’ and compared with the guided annotation using ‘gffcompare -r’^94^. Transcripts that overlapped with multiple reference genes were excluded, as well as transcripts with a length less than 200 bp, and ‘class codes’ identified by gffcompare equal to ‘e’, ‘s’ and ‘x’ were removed in downstream analysis. Transcripts with class codes ‘j’, ‘k’, ‘=’, ‘m’, ‘n’, ‘o’, ‘p’ and ‘y’ were assigned to corresponding reference genes. Novel transcripts (class codes belong to ‘u’ or ‘i’) with no clear reference annotation were blasted^95^ against human and mouse transcripts of protein coding and lncRNA with the parameters ‘-max_target_seqs 1 -outfmt 6 -num_threads 25 -evalue 1e-20 -strand plus -max_hsps 1’ to find the most similar human or mouse reference genes. If there were no orthologous genes of matched reference genes in the guinea pig Ensembl annotation, they were added. Other novel transcripts with known orthologous genes in the guinea pig Ensembl annotation were reassigned to known annotated genes based on whether the novel transcripts fell within the nearby loci (±3 kb) of the current annotation. Following this, the transcript and gene relationships were updated in the gene annotation results from gffcompare^94^. Gene annotations (such as miRNA, misc_RNA, Mt_rRNA, Mt_tRNA, ribozyme, rRNA, scaRNA, scoRNA, snRNA and sRNA) that were not included in the genome-guided assembly were included based on their Ensembl annotations in the final reference genome annotation for gene expression quantification using featureCounts with the parameters ‘-p -C -D 5000’. Using this genome-guided approach, we were then able to successfully identify the previously missing *SOX2* gene, which was absent in the Ensembl annotation. We also accurately identified the 3′ boundary of *NANOG* (ENSCPOG00000008888^96^), *CDX2* and *BMP2*, which were previously misannotated. Additionally, a new isoform (MSTRG.64950.1) of *SOX17*, which was not annotated in Ensembl and RefSeq, was discovered (Supplementary Fig. 1). Together, these improvements made a noticeable impact on our downstream analysis.

### Quality control, normalization and dimensional reduction

Following optimization of the transcriptome mapping, low-quality cells were removed based on three criteria, resulting in 541 cells remaining out of the 661 initially sequenced cells. First, each cell was required to express more than 3,000 genes. Second, the expression proportion of mitochondrial genes had to be less than 5%. Third, the expression proportion of genes related to stress granules^97^ needed to be less than 8.5% (Extended Data Fig. 3b). Following the filtering process, single-cell RNA-seq expression data were analysed using the standard Seurat (v4.2.0)^98^ pipeline. Specifically, genes expressed in at least three cells were log-normalized for downstream analysis, and the 2,000 most variable genes were identified using the ‘vst’ method with the ‘FindVariableFeatures’ function. After assessing the variance explained by technical factors (Supplementary Fig. 2a) with scater (v1.18.3)^99^, the variance attributed to the ‘number of expressed genes’ was regressed out using the ‘ScaleData’ function. The top 15 principal components (PCs) were then calculated using the ‘RunPCA’ function and utilized for UMAP dimensional reduction with 'RunUMAP' function. Clusters were identified using the ‘FindClusters’ function. Cluster stability was analysed with the R package clustree (v0.4.3)^100^ on a range of resolution values (0.4–1.4) (Supplementary Fig. 2c), with 0.6 yielding the most stable set of clusters. Cell identities were inferred based on previously published lineage markers and cell stages^19^. Marker genes were identified using the ‘FindAllMarkers’ function with default parameters, setting the cutoff of a Bonferroni-adjusted *P* value calculated by the Wilcoxon test at less than 0.05. Cells belonging to the 8-cell, 16-cell and precavitation cells from the 16–32-cell groups were combined as ‘prelineage’, and ICM and EPI cells were grouped together for marker gene expression analysis. Dimensional reduction for TE sublineages was carried out using only the TE cells and top 1,500 variable genes and top 20 PCs. Pseudo-bulk expression was calculated using the ‘AverageExpression’ function from the Seurat package.

### Trajectory interference and pseudotime analysis

For the initial analysis to determine the timing of the ICM/EPI and TE segregation, single-cell trajectory analysis was conducted using the R Monocle2 package (v2.14.0) with the DDRTree method and default parameters^101^ using prelineage, ICM/EPI and TE cells. Specifically, raw counts were used as input for Monocle2 normalization. We selected the 450 top DEGs pre-identified by the ‘FindAllMarkers’ function among clusters to construct the single-cell trajectory, and calculated pseudotime using ‘reduceDimension’ and ‘orderCells’ functions from Monocle2. To determine the timing of the second lineage specification (EPI and PE), we performed analysis on the ICM trajectory by only selecting the ICM/EPI, PE and precavitation cells. The ‘DynamicHeatmap’ function with the parameters ‘min_expcells=5, r.sq=0, dev.expl=0’ from the R package SCP (v0.5.6, https://github.com/zhanghao-njmu/SCP) was utilized to identify transcription factor genes significantly associated with pseudotime, as calculated by Monocle2, for the EPI and TE trajectories. The pseudotime trajectories for human preimplantation embryos were taken from ref. ^25^. Using the Seurat pipeline, we generated two-dimensional UMAP based on the top 1,500 variable genes and top 20 PCs. The data were then converted into a Monocle3 (v1.0.0)^102^ object using the ‘as.cell_data_set’ function from the R SeuratWrappers package (v0.3.0) (https://github.com/satijalab/seurat-wrappers). These cells were further categorized into seven subclusters using the ‘cluster_cells’ function with the ‘louvain’ method. Single-cell trajectory and pseudotime were further inferred using the ‘learn_graph’ and ‘order_cells’ functions from Monocle3, with the precavitation cells having the lowest UMAP_2 values designated as the root cell. Among the seven identified clusters (Extended Data Fig. 5a), careful examination of the distribution of stages and inferred pseudotime revealed that the majority of cells in clusters 3, 4, 5 and 7 belonged to the MB and LB stages (Extended Data Fig. 5b,c). Based on immunostaining (Fig. 1b), we knew that ICM cells do not persist into the MB stages (cluster 7), where we already observed a defined EPI and PE. Therefore, we identified cluster 2 to represent the ICM, as 52.5% of the cells belonged to the 16–32 cell and 47.5% of the cells belonged to EB but located before the bifurcation point of the EPI and PE branches (Extended Data Fig. 5a,c). To validate this, we utilized known ICM-specific markers recently identified in the human embryo, including *SPIC*^38^. We attempted to validate SPIC protein expression in the guinea pig; however, the antibody displayed unspecific binding. We have listed it in Supplementary Table 10, which can serve as an antibody resource for future studies using the guinea pig. In the mouse and human, the protein expression of SPIC has not been performed; however, *SPIC* mRNA is highly expressed in the prelineage and ICM and then drastically declines in the EPI based on our reanalysis of published datasets (Extended Data Fig. 5e,f)^30,39,40^. In the guinea pig, we observed high expression of *SPIC* in clusters 6, 1, 2 and 7, with a sharp drop in expression in clusters 3, 4 and 5. This decrease in expression coincided with the bifurcation point during which the specification of EPI and PE occurred, confirming the identification of the ICM as cluster 2 (Extended Data Fig. 5d).

### Differential expression and GSEA

Differential gene expression analysis was conducted for EPI versus PE, EPI versus TE, and mural versus polar using the Wilcoxon test implemented in the FindMarkers function from the Seurat package by assuming the data met the assumptions of this test, though this was not formally tested. Genes with a Bonferroni-adjusted *P* value of less than 0.05, a log_2_(fold change) greater than 0.25, and expression in more than 10% of the cells were considered differentially expressed. Functional enrichment analysis of DEGs and GSEA of genes with log_2_(fold change) greater than 0.1 was performed using the ‘enricher’ and ‘GSEA’ function from the clusterProfiler package (v3.18.1)^103^.

### Reprocessing scRNA-seq primate and mouse datasets

Human preimplantation embryo transcriptomes were compiled from two single-cell profiling studies^30,58^. These data were reprocessed as reported in ref. ^25^. Updated annotations from ref. ^19^ were used for the Petropoulos et al. dataset^30^. Embryos with lineage segregation at E5 were designated as ‘early blastocysts’, and those at E6 and E7 were designated as ‘middle blastocysts’ and ‘late blastocysts’, respectively. Preimplantation marmoset embryo scRNA-seq transcriptomes were selected from studies by Boroviak et al.^34^ and Bergmann and colleagues^104^. Expression matrices from both projects were extracted from https://github.com/Boroviak-Lab/SpatialModelling. The EPI and PE cells in ‘late blastocysts’ from ref. ^34^, were further identified through clustering and expression of EPI and PE known markers as reported in their paper. TE cells from ref. ^104^ were designated as ‘middle–late blastocyst TE’. Preimplantation cynomolgus monkey embryo scRNA-seq transcriptomes were taken from ref. ^35^. The data from ref. ^35^ were reprocessed and remapped using STAR (v2.5.1b)^105^ with the reference genome ‘Macaca_fascicularis_5.0.96’ from Ensembl. Gene expression was quantified using RSEM (v1.3.0)^106^. Previously published annotations downloaded from their publication were used. Embryos belonging to E6 and E7 were designated as ‘early blastocysts’, and those at E8 and E9 were designated as ‘middle or late blastocysts’. Preimplantation mouse embryo datasets were selected from three earlier studies by Deng et al.^39^, Posfai et al.^33^ and Nowotschin and colleagues^40^. Data from ref. ^39^ and ref. ^33^ were reprocessed and remapped using STAR and RSEM as well. Expression matrices from ref. ^40^ were extracted from the original publication. During annotation, for the Posfai et al. dataset^33^, 88 cells were excluded because they were annotated as ‘ICM’ or ‘TE’ but clustered with the ‘CO’ cluster, to avoid ambiguous annotations. EPI, PE and TE annotations from Deng et al.^39^, as well as EPI and PE annotations from Posfai et al.^33^, were reannotated by integrating them with the E3.5 and E4.5 datasets from ref. ^40^ using CCA with 1,000 anchor genes and the top 15 PCs. Because the E3.5 and E4.5 datasets from ref. ^40^ required integration, to avoid stage misassignment between mouse, human or guinea pig, only datasets from ref. ^39^ and ref. ^33^ were used for cross-species integration. Lineage marker comparisons were performed between 8-cell and 16-cell stages in ref. ^39^ and E3.5 and E4.5 cells from ref. ^40^. Cells from the E3.5 were labelled as ‘early blastocysts’, and those at E4.5 were labelled as ‘middle or late blastocysts’.

### Pairwise integration of human, mouse and guinea pig data

To align the human and guinea pig genes, we first converted their gene names to Ensembl gene IDs. We then aligned their Ensembl gene IDs based on the Ensembl orthologue information. Only genes with the orthologue type ‘ortholog_one2one’ were used for integration. After aligning gene features, preimplantation data from human^30,58^ and guinea pig embryos were integrated together using the CCA approach implemented in the R package Seurat. Anchors between the two species were identified using the ‘FindIntegrationAnchors’ function with the top 750 features selected by the ‘SelectIntegrationFeatures’ function. Subsequently, the data were further integrated using the ‘IntegrateData’ function with the following parameters: ‘k.anchor = 5; k.score = 30; k.weight = 50; k.filter = 100;’. After integration, PCA was performed on the integrated data, followed by embedding into UMAP space based on the top 20 PCA dimensions. To align the developmental stages and lineages, MNNs between the guinea pig and human preimplantation embryos were identified using the ‘findMutualNN’ function from the R package batchelor (v1.6.2)^107^ on the scaled data with a setting of ‘k = 20’. Pearson correlation between human and guinea pig cells identified as MNNs was calculated on scaled data from the integrated object. The same process and parameters were applied to the integration of preimplantation data from mouse embryos^33,39^ and guinea pig embryos, as well as for the integration of human^30,58^ and mouse^33,39^ data.

### Comparison of human, mouse and guinea pig lineage markers

Lineage markers for human, mouse and guinea pig were identified using the same ‘FindAllMarker’ function. The union gene set for human, mouse and guinea pig lineage markers was used to assess the differences among these species. Given that the expression of genes in embryonic cells is not always on/off among the lineages, we restricted our analysis to consider a gene within a specific lineage if it was the highest expressed in that respective lineage when compared to the others. Shared lineage markers were identified for genes with the same gene names expressed in 10% of the cells and displaying the highest expression in the same corresponding lineages across species.

### Reporting summary

Further information on research design is available in the Nature Portfolio Reporting Summary linked to this Article.

## Online content

Any methods, additional references, Nature Portfolio reporting summaries, source data, extended data, supplementary information, acknowledgements, peer review information; details of author contributions and competing interests; and statements of data and code availability are available at 10.1038/s41556-025-01642-9.

## Supplementary information

Supplementary Information Supplementary Figs. 1–11 and source data for Supplementary Figs. 5–8 and 11.

Reporting Summary

Supplementary Table 1 Meta table for cells that have passed scRNA-seq quality control.

Supplementary Table 2 Lineage markers for guinea pig preimplantation. *P* values were calculated by a two-sided Wilcoxon test and adjusted using the ‘Bonferroni’ method.

Supplementary Table 3 Transcription factors related to the EPI and TE trajectories. *P* values were calculated by a two-sided test and adjusted using the ‘Holm’ method.

Supplementary Table 4 Differentially expressed genes and GSEA results from EPI versus TE and EPI versus PE in guinea pig, human and mouse. *P* values of DEGs were calculated by a two-sided Wilcoxon test and adjusted using the ‘Bonferroni’ method. *P* values of GSEA were calculated by a one-sided ‘Kolmogorov–Smirnov’ test.

Supplementary Table 5 Differentially expressed genes and enriched pathways between mural and polar TE cells in guinea pig, human and mouse. *P* values were calculated by a two-sided Wilcoxon test and adjusted using the ‘Bonferroni’ method.

Supplementary Table 6 Table showing identified lineage markers, their average expression, and species conservation in humans, mice and guinea pigs.

Supplementary Table 7 Summary of preimplantation developmental features in human and small animal models.

Supplementary Table 8 In vitro culture conditions tested for guinea pig embryos.

Supplementary Table 9 Guinea pig modified N2B27 embryo culture medium composition (without insulin) used in functional analysis of the PI3K-AKT signalling pathway.

Supplementary Table 10 Primary antibodies used in guinea pig embryos.

Supplementary Table 11 Guinea pig modified N2B27 embryo culture medium composition (without retinoic acid) used in in vitro implantation on ibidi plate.

Source Data for Supplementary Fig. 1 Source data for Supplementary figures.

## Source data

Source Data Figs. 1–7 and Source Data Extended Data Figs. 1–9 Statistical source data

## Data Availability

Sequencing data that support the findings of this study have been deposited in the Gene Expression Omnibus (GEO) under accession code GSE253670. Previously published datasets utilized in this study include the human embryonic datasets (Yanagida et al. (GSE171820)^58^ and Petropoulos et al. (E-MTAB-3929)^30^), two embryonic datasets from *Callithrix jacchus* (marmoset) (Bergmann et al. (E-MTAB-9367)^104^ and Boroviak et al. (E-MTAB-7078)^34^) and one embryonic dataset from *Macaca fascicularis* (crab-eating macaque) (Nakamura et al. (GSE74767)^35^). Four mouse embryo datasets were selected from Deng et al. (GSE45719)^39^, Nowotschin et al. (GSE123046)^40^, Posfai et al. (GSE84892)^33^ and Liu et al. (GSE133200)^55^. Data supporting the findings of this study are available from the corresponding author on reasonable request. Source data are provided with this paper.

## References

[1] Biondic, S., Canizo, J., Vandal, K., Zhao, C. & Petropoulos, S. Cross-species comparison of mouse and human preimplantation development with an emphasis on lineage specification. *J. Reprod. Fertil.* **165**, R103–R116 (2023). 10.1530/REP-22-0144 36700623

[2] Gerri, C., Menchero, S., Mahadevaiah, S. K., Turner, J. M. A. & Niakan, K. K. Human embryogenesis: a comparative perspective. *Annu. Rev. Cell Dev. Biol.* **36**, 411–440 (2020). 33021826 10.1146/annurev-cellbio-022020-024900

[3] Gerri, C. et al. A conserved role of the Hippo signalling pathway in initiation of the first lineage specification event across mammals. *Development* **150**, dev201112 (2023). 36971487 10.1242/dev.201112 PMC10263151

[4] Bouchereau, W. et al. Major transcriptomic, epigenetic and metabolic changes underlie the pluripotency continuum in rabbit preimplantation embryos. *Development* **149**, dev200538 (2022). 35993311 10.1242/dev.200538

[5] Wagner, J. E. in *The Biology of the Guinea Pig* (eds Wagner, J. E. & Manning, P. J.) Ch. 1, 1–4 (Academic Press, 1976).

[6] Suzuki, O. et al. Optimization of superovulation induction by human menopausal gonadotropin in guinea pigs based on follicular waves and FSH-receptor homologies. *Mol. Reprod. Dev.* **64**, 219–225 (2003). 12506355 10.1002/mrd.10242

[7] Morrison, J. L. et al. Guinea pig models for translation of the developmental origins of health and disease hypothesis into the clinic. *J. Physiol.* **596**, 5535–5569 (2018). 29633280 10.1113/JP274948 PMC6265540

[8] Dorsch, M. M., Glage, S. & Hedrich, H. J. Collection and cryopreservation of preimplantation embryos of *Cavia porcellus*. *Lab. Anim.* **42**, 489–494 (2008). 18782822 10.1258/la.2007.007011

[9] Hribal, R., Guenther, A., Rübensam, K. & Jewgenow, K. Blastocyst recovery and multifactorial gene expression analysis in the wild guinea pig (*Cavia aperea*). *Theriogenology* **86**, 1299–1307 (2016). 27264741 10.1016/j.theriogenology.2016.04.071

[10] Suzuki, O. et al. Development of preimplantation guinea-pig embryos in serum-free media. *Reprod. Fertil. Dev.* **5**, 425–432 (1993). 8153392 10.1071/rd9930425

[11] Lee, K. Y. & DeMayo, F. J. Animal models of implantation. *Reproduction* **128**, 679–695 (2004). 15579585 10.1530/rep.1.00340

[12] Pfeffer, P. L. Alternative mammalian strategies leading towards gastrulation: losing polar trophoblast (Rauber’s layer) or gaining an epiblast cavity. *Philos. Trans. R. Soc. Lond. B Biol. Sci.* **377**, 20210254 (2022). 36252216 10.1098/rstb.2021.0254 PMC9574635

[13] Kaufmann, P., Black, S. & Huppertz, B. Endovascular trophoblast invasion: implications for the pathogenesis of intrauterine growth retardation and preeclampsia. *Biol. Reprod.* **69**, 1–7 (2003). 12620937 10.1095/biolreprod.102.014977

[14] Carter, A. M. et al. Comparative placentation and animal models: patterns of trophoblast invasion—a workshop report. *Placenta* **27**, S30–S33 (2006). 16529811 10.1016/j.placenta.2006.01.008

[15] Suckow, M. A., Stevens, K. A. & Wilson, R. P. *The Laboratory Rabbit*, *Guinea Pig*, *Hamster and Other Rodents* (Academic Press, 2012).

[16] Kapoor, A. & Matthews, S. G. Short periods of prenatal stress affect growth, behaviour and hypothalamo–pituitary–adrenal axis activity in male guinea pig offspring. *J. Physiol.* **566**, 967–977 (2005). 15932885 10.1113/jphysiol.2005.090191 PMC1464791

[17] Deanesly, R. Implantation and early pregnancy in ovariectomized guinea-pigs. *J. Reprod. Fertil.* **1**, 242–248 (1960).

[18] Nikas, G., Ao, A., Winston, R. M. & Handyside, A. H. Compaction and surface polarity in the human embryo in vitro. *Biol. Reprod.* **55**, 32–37 (1996). 8793055 10.1095/biolreprod55.1.32

[19] Meistermann, D. et al. Integrated pseudotime analysis of human pre-implantation embryo single-cell transcriptomes reveals the dynamics of lineage specification. *Cell Stem Cell* **28**, 1625–1640.e6 (2021). 34004179 10.1016/j.stem.2021.04.027

[20] Niakan, K. K. & Eggan, K. Analysis of human embryos from zygote to blastocyst reveals distinct gene expression patterns relative to the mouse. *Dev. Biol.* **375**, 54–64 (2013). 23261930 10.1016/j.ydbio.2012.12.008

[21] Gerri, C. et al. Initiation of a conserved trophectoderm program in human, cow and mouse embryos. *Nature* **587**, 443–447 (2020). 32968278 10.1038/s41586-020-2759-x PMC7116563

[22] Wicklow, E. et al. HIPPO pathway members restrict SOX2 to the inner cell mass where it promotes ICM fates in the mouse blastocyst. *PLoS Genet.* **10**, e1004618 (2014). 25340657 10.1371/journal.pgen.1004618 PMC4207610

[23] Frum, T., Murphy, T. M. & Ralston, A. HIPPO signaling resolves embryonic cell fate conflicts during establishment of pluripotency in vivo. *eLife* **7**, e42298 (2018). 30526858 10.7554/eLife.42298 PMC6289571

[24] Cauffman, G., De Rycke, M., Sermon, K., Liebaers, I. & Van de Velde, H. Markers that define stemness in ESC are unable to identify the totipotent cells in human preimplantation embryos. *Hum. Reprod.* **24**, 63–70 (2009). 18824471 10.1093/humrep/den351

[25] Zhao, C. et al. A comprehensive human embryo reference tool using single-cell RNA-sequencing data. *Nat. Methods*10.1038/s41592-024-02493-2 (2024). 39543283 10.1038/s41592-024-02493-2 PMC11725501

[26] Strumpf, D. et al. Cdx2 is required for correct cell fate specification and differentiation of trophectoderm in the mouse blastocyst. *Development* **132**, 2093–2102 (2005). 15788452 10.1242/dev.01801

[27] Niwa, H. et al. Interaction between Oct3/4 and Cdx2 determines trophectoderm differentiation. *Cell* **123**, 917–929 (2005). 16325584 10.1016/j.cell.2005.08.040

[28] Nishioka, N. et al. The Hippo signaling pathway components Lats and Yap pattern Tead4 activity to distinguish mouse trophectoderm from inner cell mass. *Dev. Cell* **16**, 398–410 (2009). 19289085 10.1016/j.devcel.2009.02.003

[29] Goissis, M. D. & Cibelli, J. B. Functional characterization of CDX2 during bovine preimplantation development in vitro. *Mol. Reprod. Dev.* **81**, 962–970 (2014). 25251051 10.1002/mrd.22415

[30] Petropoulos, S. et al. Single-cell RNA-seq reveals lineage and X chromosome dynamics in human preimplantation embryos. *Cell* **165**, 1012–1026 (2016). 27062923 10.1016/j.cell.2016.03.023 PMC4868821

[31] Borensztein, M. et al. Xist-dependent imprinted X inactivation and the early developmental consequences of its failure. *Nat. Struct. Mol. Biol.* **24**, 226–233 (2017). 28134930 10.1038/nsmb.3365 PMC5337400

[32] Stirparo, G. G. et al. Integrated analysis of single-cell embryo data yields a unified transcriptome signature for the human pre-implantation epiblast. *Development* **145**, dev169672 (2018). 29361568 10.1242/dev.158501 PMC5818005

[33] Posfai, E. et al. Position- and Hippo signaling-dependent plasticity during lineage segregation in the early mouse embryo. *eLife* **6**, e22906 (2017). 28226240 10.7554/eLife.22906 PMC5370188

[34] Boroviak, T. et al. Single cell transcriptome analysis of human, marmoset and mouse embryos reveals common and divergent features of preimplantation development. *Development* **145**, dev167833 (2018). 30413530 10.1242/dev.167833 PMC6240320

[35] Nakamura, T. et al. A developmental coordinate of pluripotency among mice, monkeys and humans. *Nature* **537**, 57–62 (2016). 27556940 10.1038/nature19096

[36] Ma, H. et al. In vitro culture of cynomolgus monkey embryos beyond early gastrulation. *Science* **366**, eaax7890 (2019). 31672918 10.1126/science.aax7890

[37] Bernardo, A. S. et al. Mammalian embryo comparison identifies novel pluripotency genes associated with the naïve or primed state. *Biol. Open* **7**, 170182 (2018). 10.1242/bio.033282 PMC6124576 30026265

[38] Radley, A., Corujo-Simon, E., Nichols, J., Smith, A. & Dunn, S.-J. Entropy sorting of single-cell RNA sequencing data reveals the inner cell mass in the human pre-implantation embryo. *Stem Cell Rep.* **18**, 47–63 (2023). 10.1016/j.stemcr.2022.09.007 PMC9859930 36240776

[39] Deng, Q., Ramsköld, D., Reinius, B. & Sandberg, R. Single-cell RNA-seq reveals dynamic, random monoallelic gene expression in mammalian cells. *Science* **343**, 193–196 (2014). 24408435 10.1126/science.1245316

[40] Nowotschin, S. et al. The emergent landscape of the mouse gut endoderm at single-cell resolution. *Nature* **569**, 361–367 (2019). 30959515 10.1038/s41586-019-1127-1 PMC6724221

[41] Morgani, S. M. & Brickman, J. M. LIF supports primitive endoderm expansion during pre-implantation development. *Development* **142**, 3488–3499 (2015). 26395492 10.1242/dev.125021

[42] Wamaitha, S. E. et al. IGF1-mediated human embryonic stem cell self-renewal recapitulates the embryonic niche. *Nat. Commun.* **11**, 764 (2020). 32034154 10.1038/s41467-020-14629-x PMC7005693

[43] Zhao, C. et al. Single-cell multi-omics of human preimplantation embryos shows susceptibility to glucocorticoids. *Genome Res.* **32**, 1627–1641 (2022). 35948369 10.1101/gr.276665.122 PMC9528977

[44] Kastan, N. et al. Small-molecule inhibition of Lats kinases may promote Yap-dependent proliferation in postmitotic mammalian tissues. *Nat. Commun.* **12**, 3100 (2021). 34035288 10.1038/s41467-021-23395-3 PMC8149661

[45] Chazaud, C., Yamanaka, Y., Pawson, T. & Rossant, J. Early lineage segregation between epiblast and primitive endoderm in mouse blastocysts through the Grb2-MAPK pathway. *Dev. Cell* **10**, 615–624 (2006). 16678776 10.1016/j.devcel.2006.02.020

[46] Wooldridge, L. K. & Ealy, A. D. Interleukin-6 promotes primitive endoderm development in bovine blastocysts. *BMC Dev. Biol.* **21**, 3 (2021). 33430761 10.1186/s12861-020-00235-z PMC7802221

[47] Yamanaka, Y., Lanner, F. & Rossant, J. FGF signal-dependent segregation of primitive endoderm and epiblast in the mouse blastocyst. *Development* **137**, 715–724 (2010). 20147376 10.1242/dev.043471

[48] Roode, M. et al. Human hypoblast formation is not dependent on FGF signalling. *Dev. Biol.* **361**, 358–363 (2012). 22079695 10.1016/j.ydbio.2011.10.030 PMC3368271

[49] Nichols, J., Silva, J., Roode, M. & Smith, A. Suppression of Erk signalling promotes ground state pluripotency in the mouse embryo. *Development* **136**, 3215–3222 (2009). 19710168 10.1242/dev.038893 PMC2739140

[50] Piliszek, A., Madeja, Z. E. & Plusa, B. Suppression of ERK signalling abolishes primitive endoderm formation but does not promote pluripotency in rabbit embryo. *Development* **144**, 3719–3730 (2017). 28935706 10.1242/dev.156406 PMC5675450

[51] Canizo, J. R. et al. A dose-dependent response to MEK inhibition determines hypoblast fate in bovine embryos. *BMC Dev. Biol.* **19**, 13 (2019). 31272387 10.1186/s12861-019-0193-9 PMC6610975

[52] Ramos-Ibeas, P. et al. Pluripotency and X chromosome dynamics revealed in pig pre-gastrulating embryos by single cell analysis. *Nat. Commun.* **10**, 500 (2019). 30700715 10.1038/s41467-019-08387-8 PMC6353908

[53] Bessonnard, S., Vandormael-Pournin, S., Coqueran, S., Cohen-Tannoudji, M. & Artus, J. PDGF signaling in primitive endoderm cell survival is mediated by PI3K-mTOR through p53-independent mechanism: PDGF signaling in PrE survival. *Stem Cells* **37**, 888–898 (2019). 30913328 10.1002/stem.3008

[54] Iyer, D. P. et al. mTOR activity paces human blastocyst stage developmental progression. *Cell* **187**, 6566–6583.e22 (2024). 39332412 10.1016/j.cell.2024.08.048 PMC7617234

[55] Liu, D. et al. Primary specification of blastocyst trophectoderm by scRNA-seq: new insights into embryo implantation. *Sci. Adv.* **8**, eabj3725 (2022). 35947672 10.1126/sciadv.abj3725 PMC9365277

[56] Hubert, M. A., Sherritt, S. L., Bachurski, C. J. & Handwerger, S. Involvement of transcription factor NR2F2 in human trophoblast differentiation. *PLoS One* **5**, e9417 (2010). 20195529 10.1371/journal.pone.0009417 PMC2828470

[57] Stuart, T. et al. Comprehensive integration of single-cell data. *Cell* **177**, 1888–1902.e21 (2019). 31178118 10.1016/j.cell.2019.05.031 PMC6687398

[58] Yanagida, A. et al. Naive stem cell blastocyst model captures human embryo lineage segregation. *Cell Stem Cell* **28**, 1016–1022.e4 (2021). 33957081 10.1016/j.stem.2021.04.031 PMC8189436

[59] Blakeley, P. et al. Defining the three cell lineages of the human blastocyst by single-cell RNA-seq. *Development* **142**, 3151–3165 (2015). 26293300 10.1242/dev.123547 PMC4582176

[60] Guo, G. et al. Human naive epiblast cells possess unrestricted lineage potential. *Cell Stem Cell* **28**, 1040–1056.e6 (2021). 33831366 10.1016/j.stem.2021.02.025 PMC8189439

[61] Simon, C. S. et al. Suppression of ERK signalling promotes pluripotent epiblast in the human blastocyst. Preprint at https://www.biorxiv.org/content/10.1101/2024.02.01.578414v1.full.pdf (2024). 10.1038/s41467-025-61830-x PMC12304225 40721412

[62] Dattani, A. et al. Naive pluripotent stem cell-based models capture FGF-dependent human hypoblast lineage specification. *Cell Stem Cell* **31**, 1058–1071.e5 (2024). 38823388 10.1016/j.stem.2024.05.003

[63] Saxena, N. K. et al. Concomitant activation of the JAK/STAT, PI3K/AKT and ERK signaling is involved in leptin-mediated promotion of invasion and migration of hepatocellular carcinoma cells. *Cancer Res.* **67**, 2497–2507 (2007). 17363567 10.1158/0008-5472.CAN-06-3075 PMC2925446

[64] Blandau, R. J. Observations on implantation of the guinea pig ovum. *Anat. Rec.* **103**, 19–47 (1949). 18108866 10.1002/ar.1091030103

[65] Ton, M.-L. N. et al. An atlas of rabbit development as a model for single-cell comparative genomics. *Nat. Cell Biol.* **25**, 1061–1072 (2023). 37322291 10.1038/s41556-023-01174-0 PMC7618511

[66] Ouyang, J. F., Kamaraj, U. S., Cao, E. Y. & Rackham, O. J. L. ShinyCell: simple and sharable visualization of single-cell gene expression data. *Bioinformatics* **37**, 3374–3376 (2021). 33774659 10.1093/bioinformatics/btab209

[67] Frum, T. & Ralston, A. Cell signaling and transcription factors regulating cell fate during formation of the mouse blastocyst. *Trends Genet.* **31**, 402–410 (2015). 25999217 10.1016/j.tig.2015.04.002 PMC4490046

[68] Canizo, J., Biondic, S., Lenghan, K. V. & Petropoulos, S. Guinea pig preimplantation embryos: generation, collection and immunofluorescence. *Methods Mol. Biol.*10.1007/7651_2023_488 (2023). 10.1007/7651_2023_488 37284942

[69] Vandal, K., Biondic, S., Canizo, J. & Petropoulos, S. Manual dissociation of mammalian preimplantation embryos for single-cell genomics. *Methods Mol. Biol.*10.1007/7651_2023_494 (2023). 10.1007/7651_2023_494 37418145

[70] Mohammed, H. et al. Single-cell landscape of transcriptional heterogeneity and cell fate decisions during mouse early gastrulation. *Cell Rep.* **20**, 1215–1228 (2017). 28768204 10.1016/j.celrep.2017.07.009 PMC5554778

[71] Ramos-Ibeas, P. et al. In vitro culture of ovine embryos up to early gastrulating stages. *Development* **149**, dev199743 (2022). 35319748 10.1242/dev.199743 PMC8977095

[72] Kagawa, H. et al. Human blastoids model blastocyst development and implantation. *Nature* **601**, 600–605 (2022). 34856602 10.1038/s41586-021-04267-8 PMC8791832

[73] Gafni, O. et al. Derivation of novel human ground state naive pluripotent stem cells. *Nature* **504**, 282–286 (2013). 24172903 10.1038/nature12745

[74] Shahbazi, M., Vuoristo, S., Jedrusik, A., Shahbazi, M. & Zernicka-Goetz, M. Culture of human embryos through implantation stages in vitro. *Protoc. Exch.*10.1038/protex.2016.017 (2016).

[75] Shahbazi, M. N. et al. Self-organization of the human embryo in the absence of maternal tissues. *Nat. Cell Biol.* **18**, 700–708 (2016). 27144686 10.1038/ncb3347 PMC5049689

[76] Kam, R. K. T., Deng, Y., Chen, Y. & Zhao, H. Retinoic acid synthesis and functions in early embryonic development. *Cell Biosci.* **2**, 11 (2012). 22439772 10.1186/2045-3701-2-11 PMC3325842

[77] Picelli, S. et al. Full-length RNA-seq from single cells using Smart-seq2. *Nat. Protoc.* **9**, 171–181 (2014). 24385147 10.1038/nprot.2014.006

[78] Plath, K. et al. Role of histone H3 lysine 27 methylation in X inactivation. *Science* **300**, 131–135 (2003). 12649488 10.1126/science.1084274

[79] Dossin, F. et al. SPEN integrates transcriptional and epigenetic control of X-inactivation. *Nature* **578**, 455–460 (2020). 32025035 10.1038/s41586-020-1974-9 PMC7035112

[80] Okamoto, I. et al. Eutherian mammals use diverse strategies to initiate X-chromosome inactivation during development. *Nature* **472**, 370–374 (2011). 21471966 10.1038/nature09872

[81] Onfray, C. et al. Unraveling hallmark suitability for staging pre- and post-implantation stem cell models. *Cell Rep.* **43**, 114232 (2024). 38761378 10.1016/j.celrep.2024.114232

[82] Sahakyan, A. et al. Human naive pluripotent stem cells model X chromosome dampening and X inactivation. *Cell Stem Cell* **20**, 87–101 (2017). 27989770 10.1016/j.stem.2016.10.006 PMC5218861

[83] Zhou, F. et al. Reconstituting the transcriptome and DNA methylome landscapes of human implantation. *Nature* **572**, 660–664 (2019). 31435013 10.1038/s41586-019-1500-0

[84] Canizo, J., Vandal, K., Biondic, S. & Petropoulos, S. Whole-mount RNA, single-molecule RNA (smRNA), and DNA Fluorescence In Situ Hybridization (FISH) in mammalian embryos. *Methods Mol. Biol.*10.1007/7651_2023_490 (2023). 10.1007/7651_2023_490 37261674

[85] Maclary, E. et al. PRC2 represses transcribed genes on the imprinted inactive X chromosome in mice. *Genome Biol.* **18**, 82 (2017). 28468635 10.1186/s13059-017-1211-5 PMC5415793

[86] Vallot, C. et al. XACT noncoding RNA competes with XIST in the control of X chromosome activity during human early development. *Cell Stem Cell* **20**, 102–111 (2017). 27989768 10.1016/j.stem.2016.10.014 PMC5222720

[87] Okamoto, I. et al. The X chromosome dosage compensation program during the development of cynomolgus monkeys. *Science* **374**, eabd8887 (2021). 34793202 10.1126/science.abd8887

[88] Teklenburg, G. et al. Cell lineage specific distribution of H3K27 trimethylation accumulation in an in vitro model for human implantation. *PLoS ONE* **7**, e32701 (2012). 22412909 10.1371/journal.pone.0032701 PMC3296731

[89] Depreux, F. F., Czech, L. & Whitlon, D. S. Sex genotyping of archival fixed and immunolabeled guinea pig cochleas. *Sci. Rep.* **8**, 5156 (2018). 29581456 10.1038/s41598-018-23491-3 PMC5980087

[90] Howe, K. L. et al. Ensembl 2021. *Nucleic Acids Res.* **49**, D884–D891 (2021). 33137190 10.1093/nar/gkaa942 PMC7778975

[91] Kim, D., Paggi, J. M., Park, C., Bennett, C. & Salzberg, S. L. Graph-based genome alignment and genotyping with HISAT2 and HISAT-genotype. *Nat. Biotechnol.* **37**, 907–915 (2019). 31375807 10.1038/s41587-019-0201-4 PMC7605509

[92] Liao, Y., Smyth, G. K. & Shi, W. featureCounts: an efficient general purpose program for assigning sequence reads to genomic features. *Bioinformatics* **30**, 923–930 (2014). 24227677 10.1093/bioinformatics/btt656

[93] Pertea, M. et al. StringTie enables improved reconstruction of a transcriptome from RNA-seq reads. *Nat. Biotechnol.* **33**, 290–295 (2015). 25690850 10.1038/nbt.3122 PMC4643835

[94] Pertea, G. & Pertea, M. GFF Utilities: GffRead and GffCompare. *F1000Res*. **9**, 10.12688/f1000research.23297.2 (2020). 10.12688/f1000research.23297.1 PMC7222033 32489650

[95] Camacho, C. et al. BLAST+: architecture and applications. *BMC Bioinformatics* **10**, 421 (2009). 20003500 10.1186/1471-2105-10-421 PMC2803857

[96] Fuellen, G. & Struckmann, S. Evolution of gene regulation of pluripotency—the case for wiki tracks at genome browsers. *Biol. Direct* **5**, 67 (2010). 21190561 10.1186/1745-6150-5-67 PMC3024949

[97] Wolozin, B. & Ivanov, P. Stress granules and neurodegeneration. *Nat. Rev. Neurosci.* **20**, 649–666 (2019). 31582840 10.1038/s41583-019-0222-5 PMC6986315

[98] Hao, Y. et al. Integrated analysis of multimodal single-cell data. *Cell* **184**, 3573–3587.e29 (2021). 34062119 10.1016/j.cell.2021.04.048 PMC8238499

[99] McCarthy, D. J., Campbell, K. R., Lun, A. T. L. & Wills, Q. F. Scater: pre-processing, quality control, normalization and visualization of single-cell RNA-seq data in R. *Bioinformatics* **33**, 1179–1186 (2017). 28088763 10.1093/bioinformatics/btw777 PMC5408845

[100] Zappia, L. & Oshlack, A. Clustering trees: a visualization for evaluating clusterings at multiple resolutions. *Gigascience* **7**, giy083 (2018). 30010766 10.1093/gigascience/giy083 PMC6057528

[101] Qiu, X. et al. Reversed graph embedding resolves complex single-cell trajectories. *Nat. Methods* **14**, 979–982 (2017). 28825705 10.1038/nmeth.4402 PMC5764547

[102] Cao, J. et al. The single-cell transcriptional landscape of mammalian organogenesis. *Nature* **566**, 496–502 (2019). 30787437 10.1038/s41586-019-0969-x PMC6434952

[103] Yu, G., Wang, L.-G., Han, Y. & He, Q.-Y. clusterProfiler: an R package for comparing biological themes among gene clusters. *OMICS* **16**, 284–287 (2012). 22455463 10.1089/omi.2011.0118 PMC3339379

[104] Bergmann, S. et al. Spatial profiling of early primate gastrulation in utero. *Nature* **609**, 136–143 (2022). 35709828 10.1038/s41586-022-04953-1 PMC7614364

[105] Dobin, A. et al. STAR: ultrafast universal RNA-seq aligner. *Bioinformatics* **29**, 15–21 (2013). 23104886 10.1093/bioinformatics/bts635 PMC3530905

[106] Li, B. & Dewey, C. N. RSEM: accurate transcript quantification from RNA-seq data with or without a reference genome. *BMC Bioinformatics* **12**, 323 (2011). 21816040 10.1186/1471-2105-12-323 PMC3163565

[107] Haghverdi, L., Lun, A. T. L., Morgan, M. D. & Marioni, J. C. Batch effects in single-cell RNA-sequencing data are corrected by matching mutual nearest neighbors. *Nat. Biotechnol.* **36**, 421–427 (2018). 29608177 10.1038/nbt.4091 PMC6152897

