# Supplementary material for: The guinea pig serves as an alternative model to study human preimplantation development
Source: Nat Cell Biol. 2025 Apr 4;27(4):696–710. doi: 10.1038/s41556-025-01642-9 (PMC11991919; doi:10.1038/s41556-025-01642-9)

nature cell biology

Supplementary information

https://doi.org/10.1038/s41556-025-01642-9

The guinea pig serves as an alternative model to study human preimplantation development

In the format provided by the authors and unedited

SUPPLEMENTARY INFORMATION

This file contains Supplementary Figs. 1-11

Supplementary Fig. 1: IGV snapshot displaying the read distribution from middle and late blastocyst EPI, PE, and TE cells for *SOX2, NANOG, SOX17, BMP2, CDX2,* and *GATA3.*

Supplementary Fig. 2: Variance and resolution selection in the guinea pig scRNA-seq dataset.

Supplementary Fig. 3: Pluripotency signatures in guinea pig and human preimplantation embryos.

Supplementary Fig. 4: Signalling pathways related to lineage segregation in guinea pig, human, and mouse

Supplementary Fig. 5: Dose-response of LATs inhibitor in mouse embryos.

Supplementary Fig. 6: Hippo and MEK-ERK signalling pathways in mouse embryos.

Supplementary Fig. 7: Dose-response of PKC inhibitor treatment in guinea pig and mouse embryos.

Supplementary Fig. 8: Dose-response of PI3K-AKT inhibitor in guinea pig and mouse embryos.

Supplementary Fig. 9: Expression of mural and polar DEGs in guinea pig and human mural and polar cells.

Supplementary Fig. 10: Heatmap depicting the pseudo-bulk expression of selected genes, categorized by stages and lineages, across human, marmoset, cynomolgus monkey, guinea pig, and mouse species.

Supplementary Fig. 11: Guinea Pig Blastocysts Sex Validation.

Source Data for Supplementary Figs. 5-8 and 11

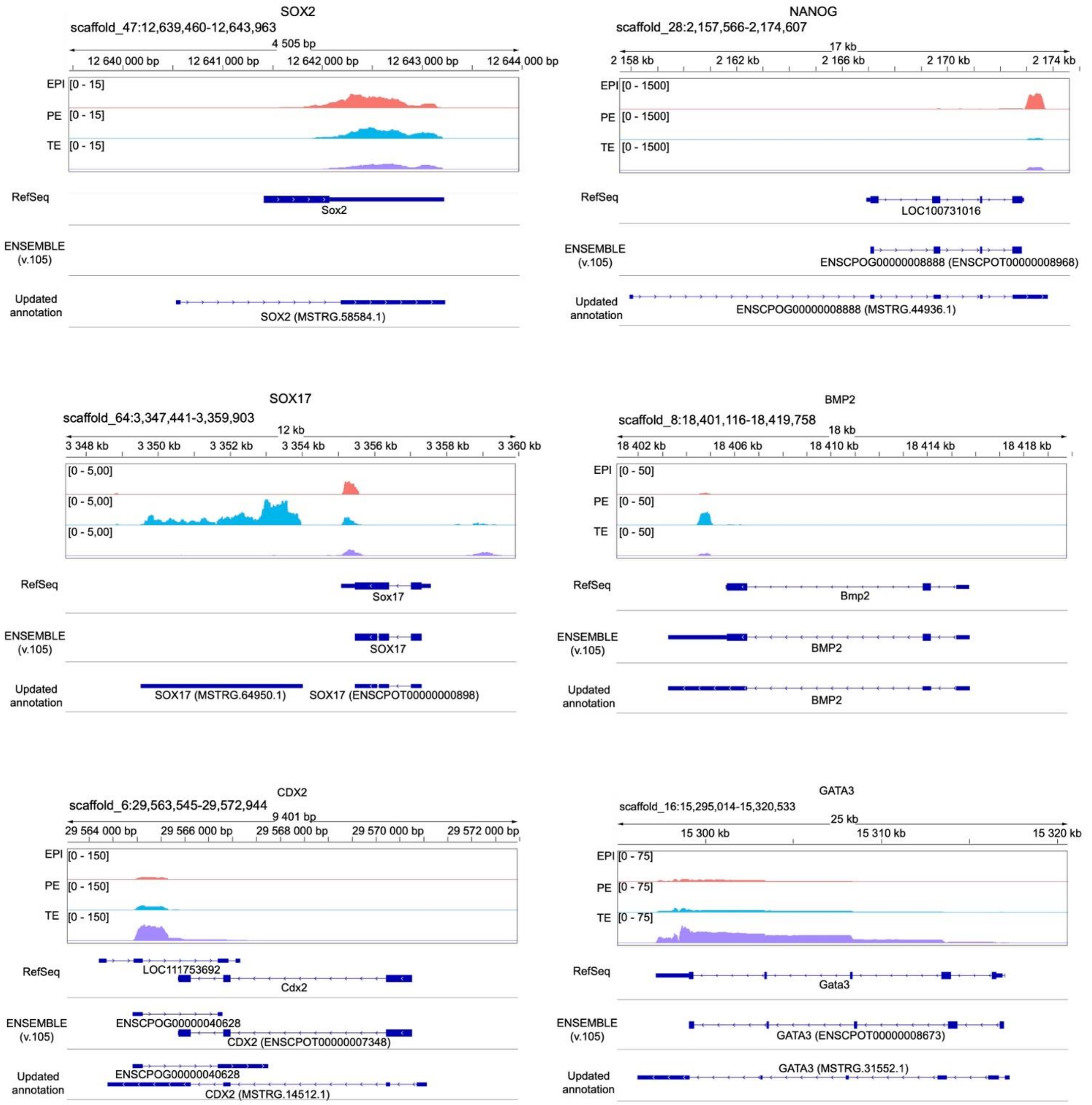

Supplementary Fig. 1 IGV snapshot displaying the read distribution from middle and late blastocyst EPI, PE, and TE cells for *SOX2*, *NANOG*, *SOX17*, *BMP2*, *CDX2*, and *GATA3*. Gene annotations from RefSeq, ENSEMBL, and updated annotations are listed.

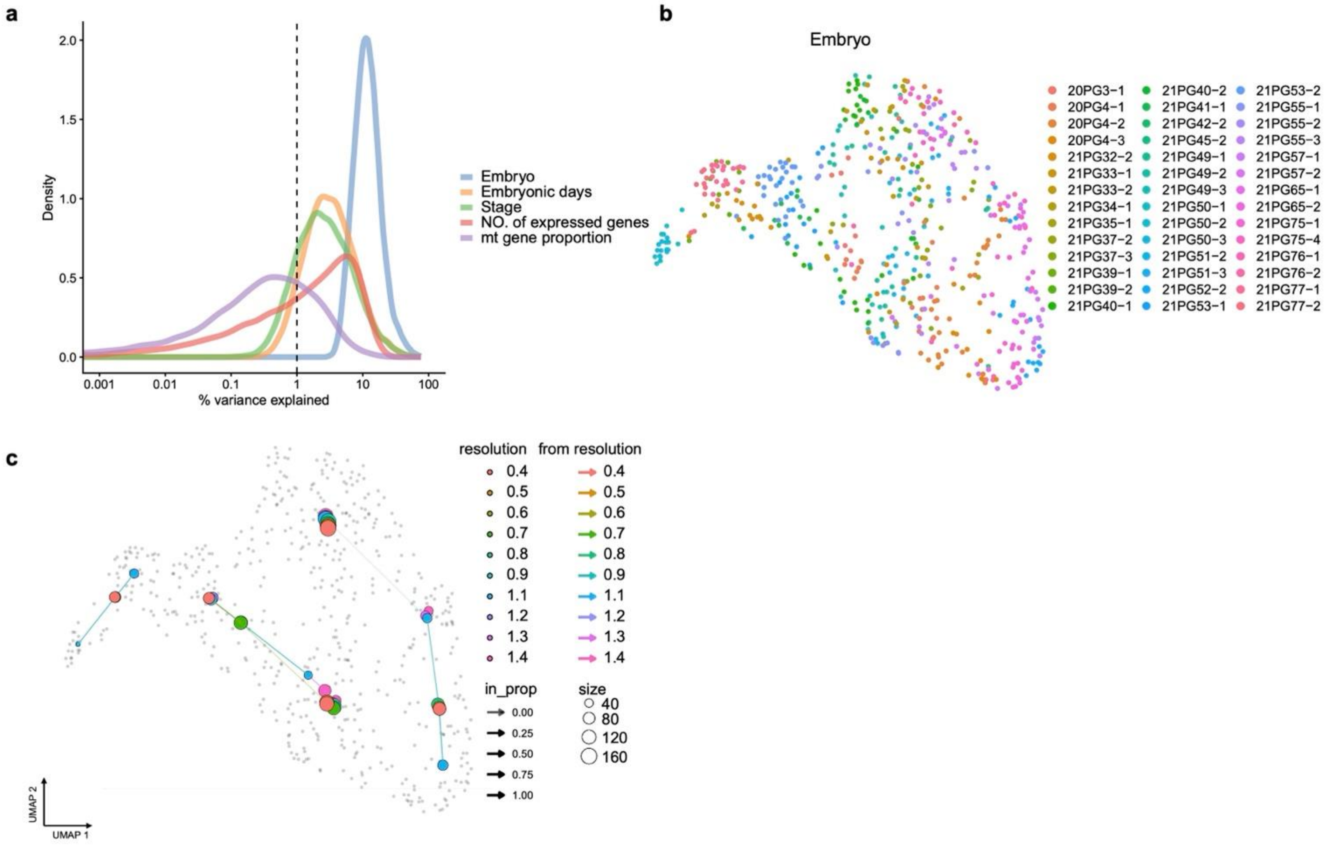

Supplementary Fig. 2 Variance and resolution selection in the guinea pig scRNA-seq dataset. a, Line plot showing the percentage of variance explained by factors "Embryo", “Embryonic days”, “Stages", “Number of expressed genes”, and "Mitochondrial gene proportion". **b**, Two-dimensional UMAP representation of 541 single-cell transcriptomes (from 42 embryos) from guinea pig preimplantation embryos, with colours indicating the embryo source. **c**, Cluster stability was analyzed with Clustree at various resolution values (0.4 to 1.4).

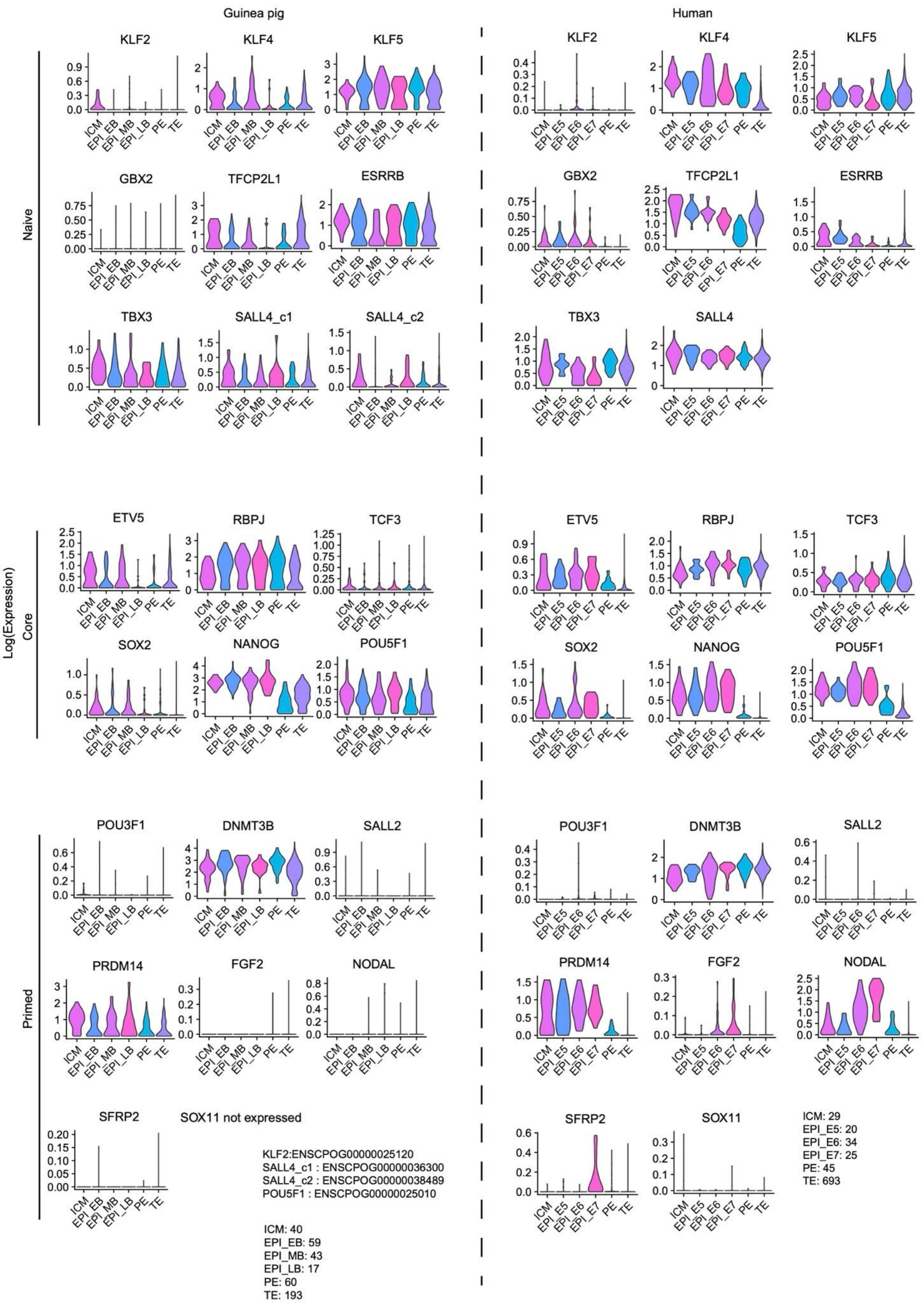
Guinea pig
Human
Naive
Core
Primed
Log(Expression)
KLF2
KLF4
KLF5
GBX2
TFCP2L1
ESRRB
TBX3
SALL4_c1
SALL4_c2
SALL4
ETV5
RBPJ
TCF3
SOX2
NANOG
POU5F1
POU3F1
DNMT3B
SALL2
PRDM14
FGF2
NODAL
SFRP2
SOX11
SOX11 not expressed
ICM
EPI_EB
EPI_MB
EPI_LB
EPI_E5
EPI_E6
EPI_E7
PE
TE
KLF2:ENSCPOG00000025120
SALL4_c1 : ENSCPOG00000036300
SALL4_c2 : ENSCPOG00000038489
POU5F1 : ENSCPOG00000025010
ICM: 40
EPI_EB: 59
EPI_MB: 43
EPI_LB: 17
PE: 60
TE: 193
ICM: 29
EPI_E5: 20
EPI_E6: 34
EPI_E7: 25
PE: 45
TE: 693

Supplementary Fig. 3 Pluripotency signatures in guinea pig and human preimplantation embryos. Violin plots showing the expression of selected pluripotency (naive, core and primed) genes in guinea pigs and humans, stratified by lineages. Published data for the human embryo was leveraged from Petropoulos et al., 2016. Cell numbers used to generate the violin plots are indicated for each lineage and species.

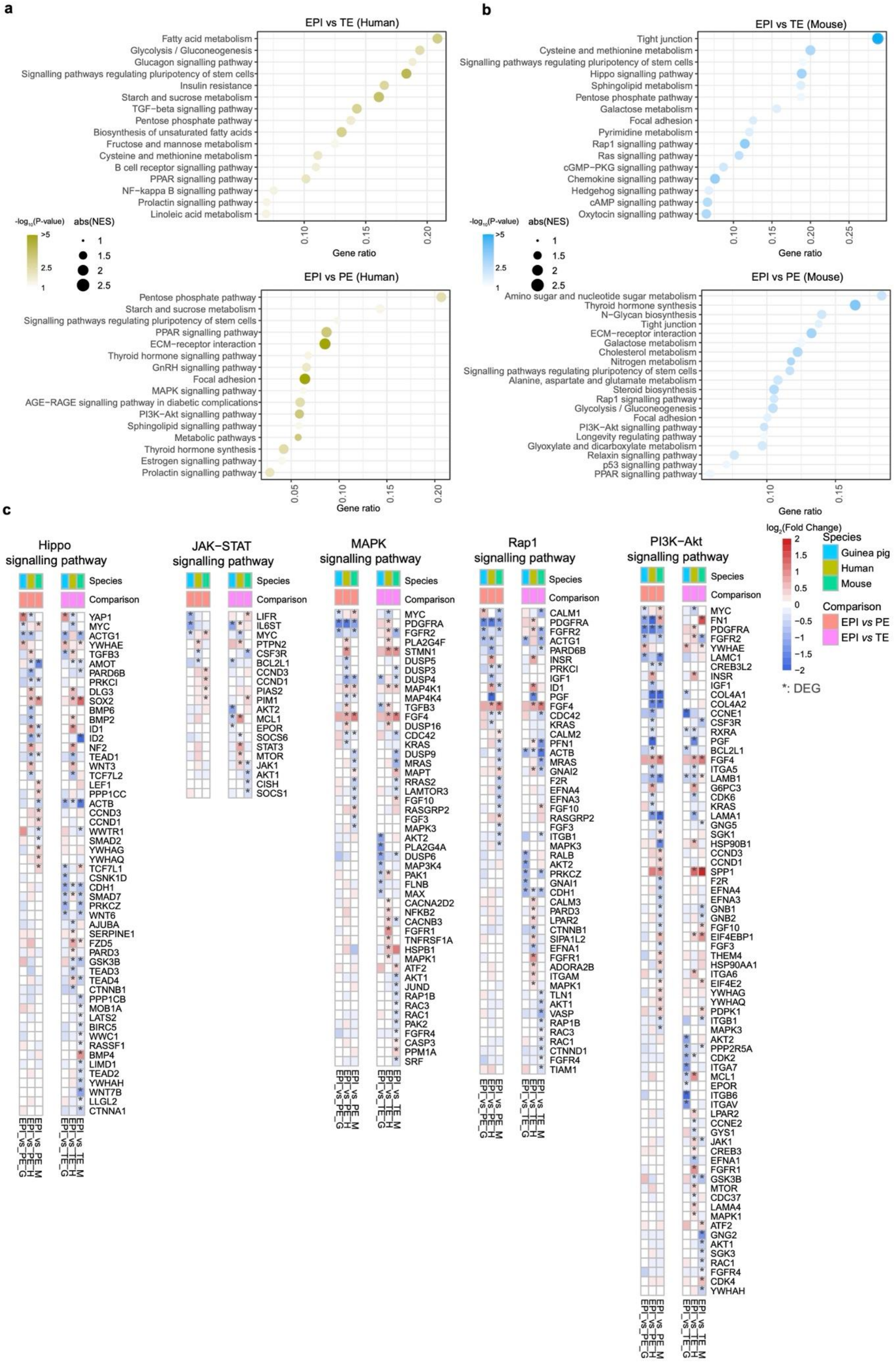
a
EPI vs TE (Human)
Fatty acid metabolism
Glycolysis / Gluconeogenesis
Glucagon signalling pathway
Signalling pathways regulating pluripotency of stem cells
Insulin resistance
Starch and sucrose metabolism
TGF−beta signalling pathway
Pentose phosphate pathway
Biosynthesis of unsaturated fatty acids
Fructose and mannose metabolism
Cysteine and methionine metabolism
B cell receptor signalling pathway
PPAR signalling pathway
NF−kappa B signalling pathway
Prolactin signalling pathway
Linoleic acid metabolism
0.10 0.15 0.20
Gene ratio
-log10(P-value)
>5
2.5
1
abs(NES)
1
1.5
2
2.5
EPI vs PE (Human)
Pentose phosphate pathway
Starch and sucrose metabolism
Signalling pathways regulating pluripotency of stem cells
PPAR signalling pathway
ECM−receptor interaction
Thyroid hormone signalling pathway
GnRH signalling pathway
Focal adhesion
MAPK signalling pathway
AGE−RAGE signalling pathway in diabetic complications
PI3K−Akt signalling pathway
Sphingolipid signalling pathway
Metabolic pathways
Thyroid hormone synthesis
Estrogen signalling pathway
Prolactin signalling pathway
0.05 0.10 0.15 0.20
Gene ratio
b
EPI vs TE (Mouse)
Tight junction
Cysteine and methionine metabolism
Signalling pathways regulating pluripotency of stem cells
Hippo signalling pathway
Sphingolipid metabolism
Pentose phosphate pathway
Galactose metabolism
Focal adhesion
Pyrimidine metabolism
Rap1 signalling pathway
Ras signalling pathway
cGMP−PKG signalling pathway
Chemokine signalling pathway
Hedgehog signalling pathway
cAMP signalling pathway
Oxytocin signalling pathway
0.10 0.15 0.20 0.25
Gene ratio
-log10(P-value)
>5
2.5
1
abs(NES)
1
1.5
2
2.5
EPI vs PE (Mouse)
Amino sugar and nucleotide sugar metabolism
Thyroid hormone synthesis
N−Glycan biosynthesis
Tight junction
ECM−receptor interaction
Galactose metabolism
Cholesterol metabolism
Nitrogen metabolism
Signalling pathways regulating pluripotency of stem cells
Alanine, aspartate and glutamate metabolism
Steroid biosynthesis
Rap1 signalling pathway
Glycolysis / Gluconeogenesis
Focal adhesion
PI3K−Akt signalling pathway
Longevity regulating pathway
Glyoxylate and dicarboxylate metabolism
Relaxin signalling pathway
p53 signalling pathway
PPAR signalling pathway
0.10 0.15
Gene ratio
c
Hippo signalling pathway
Species
Comparison
YAP1 MYC ACTG1 YWHAE TGFB3 AMOT PARD6B PRKCI DLG3 SOX2 BMP6 BMP2 ID1 ID2 NF2 TEAD1 WNT3 TCF7L2 LEF1 PPP1CC ACTB CCND3 CCND1 WWTR1 SMAD2 YWHAG YWHAQ TCF7L1 CSNK1D CDH1 SMAD7 PRKCZ WNT6 AJUBA SERPINE1 FZD5 PARD3 GSK3B TEAD3 TEAD4 CTNNB1 PPP1CB MOB1A LATS2 BIRC5 WWC1 RASSF1 BMP4 LIMD1 TEAD2 YWHAH WNT7B LLGL2 CTNNA1
EPI_vs_PE_M EPI_vs_PE_H EPI_vs_PE_G EPI_vs_TE_M EPI_vs_TE_H EPI_vs_TE_G
JAK−STAT signalling pathway
Species
Comparison
LIFR IL6ST MYC PTPN2 CSF3R BCL2L1 CCND3 CCND1 PIAS2 PIM1 AKT2 MCL1 EPOR SOCS6 STAT3 MTOR JAK1 AKT1 CISH SOCS1
MAPK signalling pathway
Species
Comparison
MYC PDGFRA FGFR2 PLA2G4F STMN1 DUSP5 DUSP3 DUSP4 MAP4K1 MAP4K4 TGFB3 FGF4 DUSP16 CDC42 KRAS DUSP9 MRAS MAPT RRAS2 LAMTOR3 FGF10 RASGRP2 FGF3 MAPK3 AKT2 PLA2G4A DUSP6 MAP3K4 PAK1 FLNB MAX CACNA2D2 NFKB2 CACNB3 FGFR1 TNFRSF1A HSPB1 MAPK1 ATF2 AKT1 JUND RAP1B RAC3 RAC1 PAK2 FGFR4 CASP3 PPM1A SRF
EPI_vs_PE_M EPI_vs_PE_H EPI_vs_PE_G EPI_vs_TE_M EPI_vs_TE_H EPI_vs_TE_G
Rap1 signalling pathway
Species
Comparison
CALM1 PDGFRA FGFR2 ACTG1 PARD6B INSR PRKCI IGF1 ID1 PGF FGF4 CDC42 KRAS CALM2 PFN1 ACTB MRAS GNAI2 F2R EFNA4 EFNA3 FGF10 RASGRP2 FGF3 ITGB1 MAPK3 RALB AKT2 PRKCZ GNAI1 CDH1 CALM3 PARD3 LPAR2 CTNNB1 SIPA1L2 EFNA1 FGFR1 ADORA2B ITGAM MAPK1 TLN1 AKT1 VASP RAP1B RAC3 RAC1 CTNND1 FGFR4 TIAM1
EPI_vs_PE_M EPI_vs_PE_H EPI_vs_PE_G EPI_vs_TE_M EPI_vs_TE_H EPI_vs_TE_G
PI3K−Akt signalling pathway
Species
Comparison
MYC FN1 PDGFRA FGFR2 YWHAE LAMC1 CREB3L2 INSR IGF1 COL4A1 COL4A2 CCNE1 CSF3R RXRA PGF BCL2L1 FGF4 ITGA5 LAMB1 G6PC3 CDK6 KRAS LAMA1 GNG5 SGK1 HSP90B1 CCND3 CCND1 SPP1 F2R EFNA4 EFNA3 GNB1 GNB2 FGF10 EIF4EBP1 FGF3 THEM4 HSP90AA1 ITGA6 EIF4E2 YWHAG YWHAQ PDPK1 ITGB1 MAPK3 AKT2 PPP2R5A CDK2 ITGA7 MCL1 EPOR ITGB6 ITGAV LPAR2 CCNE2 GYS1 JAK1 CREB3 EFNA1 FGFR1 GSK3B MTOR CDC37 LAMA4 MAPK1 ATF2 GNG2 AKT1 SGK3 RAC1 FGFR4 CDK4 YWHAH
EPI_vs_PE_M EPI_vs_PE_H EPI_vs_PE_G EPI_vs_TE_M EPI_vs_TE_H EPI_vs_TE_G
log2(Fold Change)
2 1.5 1 0.5 0 −0.5 −1 −1.5 −2
Species
Guinea pig
Human
Mouse
Comparison
EPI vs PE
EPI vs TE
*: DEG

Supplementary Fig. 4 Signalling pathways related to lineage segregation in guinea pig, human, and mouse. a and **b**, Dot plots of KEGG terms showing the results from Gene Set Enrichment Analysis (GSEA) for the EPI-TE comparison and EPI-PE comparison in human (a) and mouse (b), with P values (one-sided, permutation test) less than 0.05 indicated. The dot size indicates the absolute values of normalized enrichment score (NES), colour represents the P value, and the x-axis shows the ratio of DEGs corresponding to each KEGG term. **c**, Gene regulation associated with the Hippo signalling pathway, JAK-STAT signalling pathway, MAPK signalling pathway, Rap1 signalling pathway, and PI3K-AKT signalling pathway in guinea pigs, humans, and mice. The log2(fold change) of genes from the EPI-TE and EPI-PE comparisons is represented by colour, with DEGs identified by Bonferroni adjusted P values (two-sided, Wilcoxon test) less than 0.05 and log2(fold change) more than 0.25 marked by an asterisk (*). Species and lineage comparisons are indicated in the heatmap.

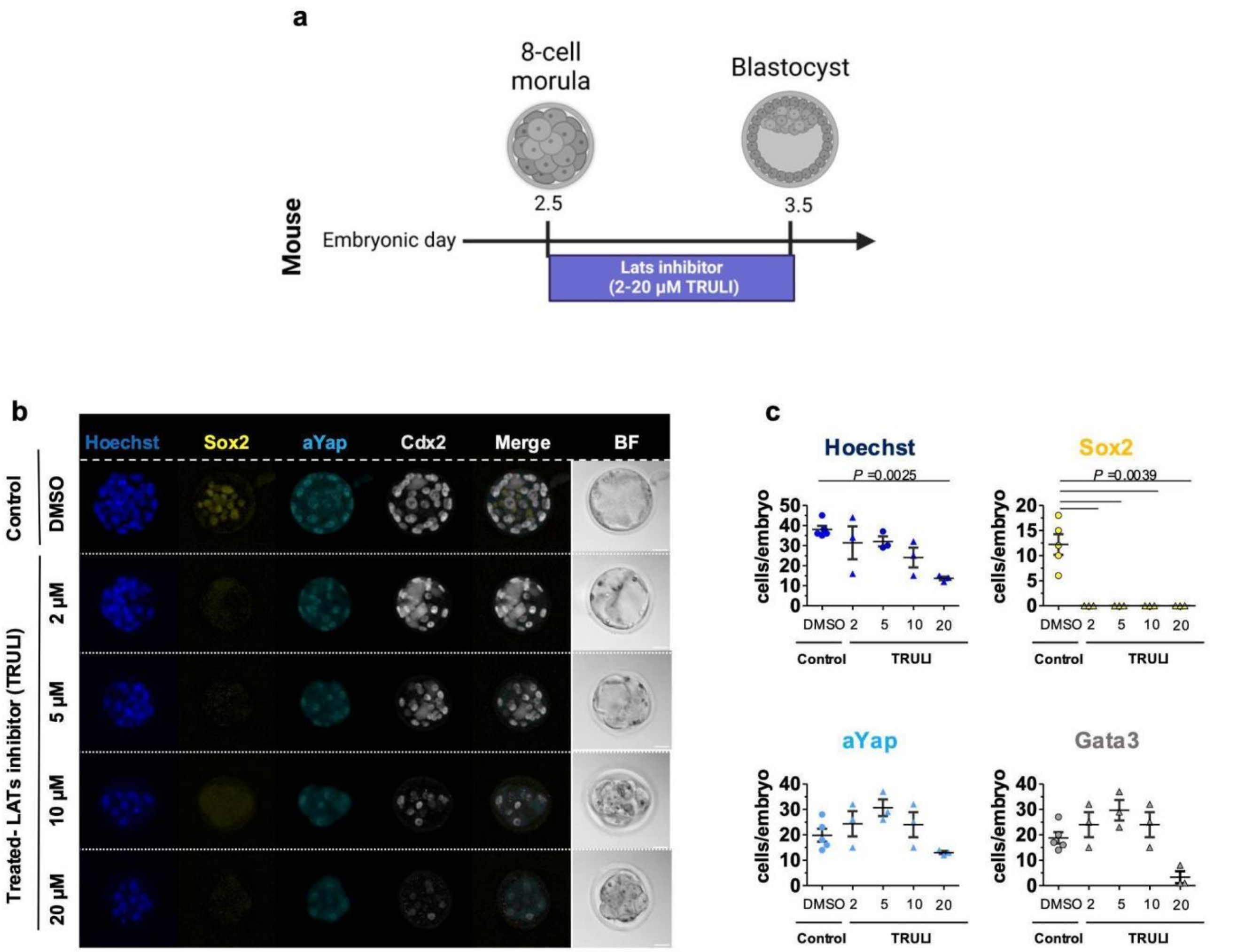

Supplementary Fig. 5 Dose-response of LATs inhibitor in mouse embryos. a, Schematic of TRULI treatments in mouse embryos. **b**, Representative immunofluorescence images of Sox2 (yellow), Cdx2 (gray), aYAP (cyan) and Hoechst nuclear staining (blue) in control (DMSO) and LATs inhibitor-treated mouse embryos with different concentrations of TRULI inhibitor (2-20 µM) from 8 cell E2.25 to blastocyst E3.5 (n = 5 for control and n = 3 for each TRULI dose). **c**, Scatter plot showing the number of cells per embryo for the indicated markers, P values stated in each figure (Kruskal-Wallis test). Schematic created in BioRender.com. Scale bars: 20 µm.

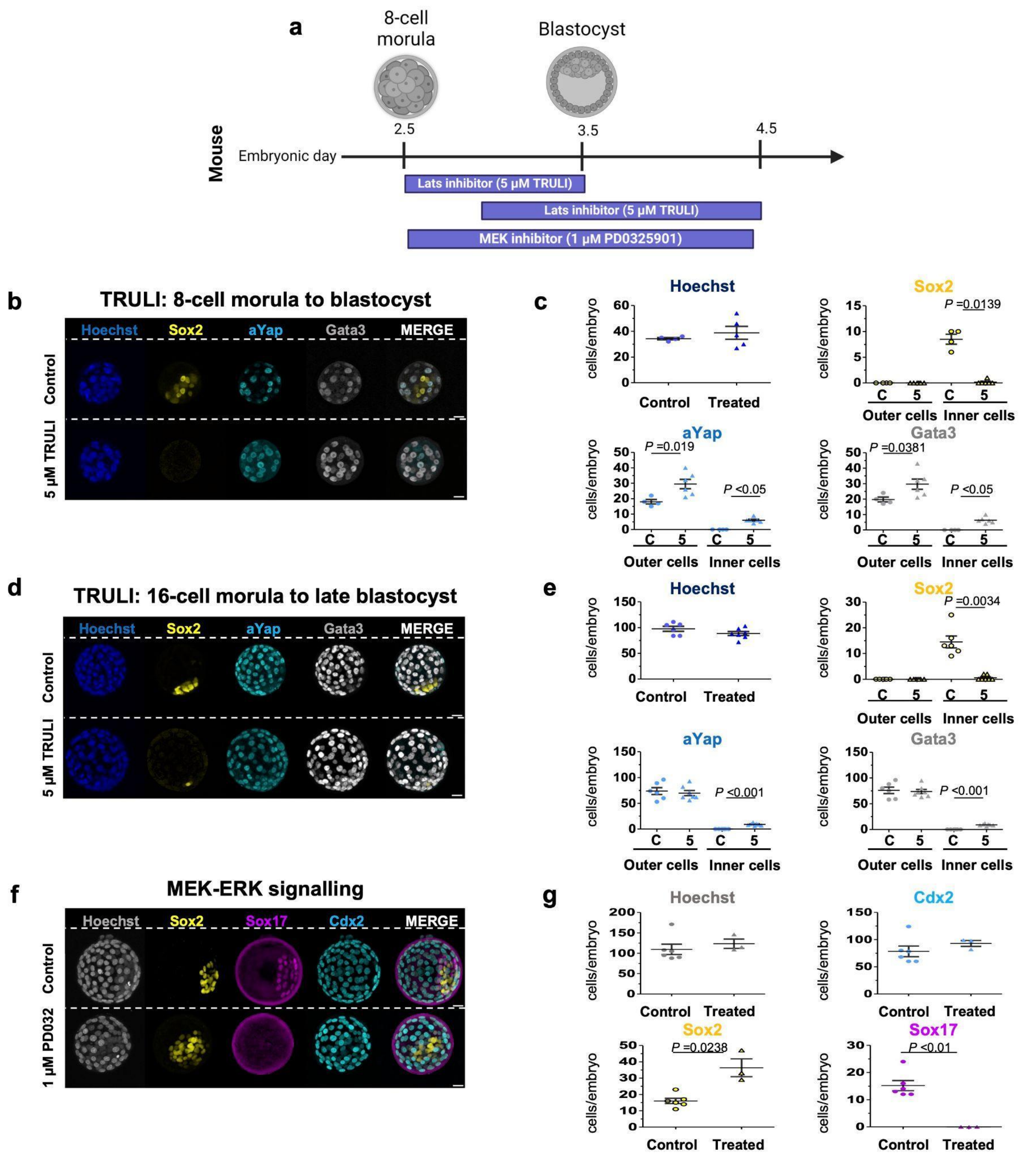

Supplementary Fig. 6 Hippo and MEK-ERK signalling pathways in mouse embryos. a, Schematic of the treatment protocol for individual small molecules used with mouse embryos. **b**, Representative immunofluorescence image of Sox2 (yellow), aYAP (cyan), Gata3 (gray) and Hoechst nuclear staining (blue) in control (DMSO) and LATS inhibitor (5 µM of TRULI) treated mouse embryos from 8-cell compacted morula to mid-blastocyst. **c**, Scatter plot showing the total number of cells per embryo (Hoechst stained) and the number of cells per embryo for the indicated markers in control (n = 4) and TRULI (n = 5) treated embryos. P values stated in each figure (two-tailed Mann-Whitney test). **d**, Representative immunofluorescence

image of Sox2 (yellow), aYAP (cyan), Gata3 (gray) and Hoechst nuclear staining (blue) in control (DMSO) and LATS inhibited (5 µM of TRULI) treated mouse embryos from 16-cell compacted morula to late blastocyst. **e**, Scatter plot showing the total number of cells per embryo (Hoechst stained) and the number of cells per embryo for the indicated markers in control (n = 6) and TRULI (n = 7) treated embryos, P values stated in each figure. **f**, Representative immunofluorescence image of Sox2 (yellow), Sox17 (magenta), Cdx2 (cyan) and Hoechst nuclear staining (gray) in control (DMSO) and MEK-ERK inhibitor (1 µM of PD0325901) mouse embryos. g, Scatter plot showing the total number of cells per embryo (Hoechst stained) and the number of cells per embryo for the indicated markers in control (n = 6) and PD0325901 (n = 3) treated embryos. P values stated in each figure (two-tailed Mann–Whitney test for a two-group comparison). Schematic created in BioRender.com. Scatter plots with mean ± s.e.m. Scale bars: 20 µm.

a

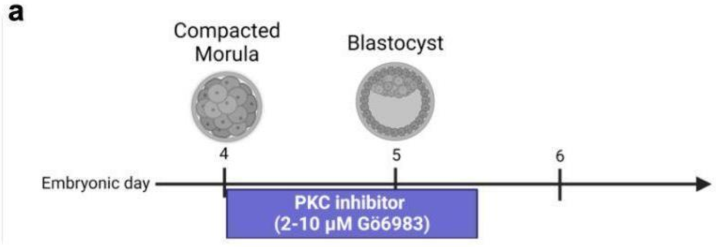

b

Medium	N	Number (%) of arrested embryos	Number (%) of morula	Number (%) of blastocyst
DMSO	12	1 (8)	2 (17)	9 (75)
2 µM Gö6983	3	0 (0)	1 (33)	2 (67)
3.5 µM Gö6983	6	0 (0)	6 (100)	0 (0)
5 µM Gö6983	3	0 (0)	3 (100)	0 (0)
10 µM Gö6983	4	3 (75)	0 (0)	1 (25)

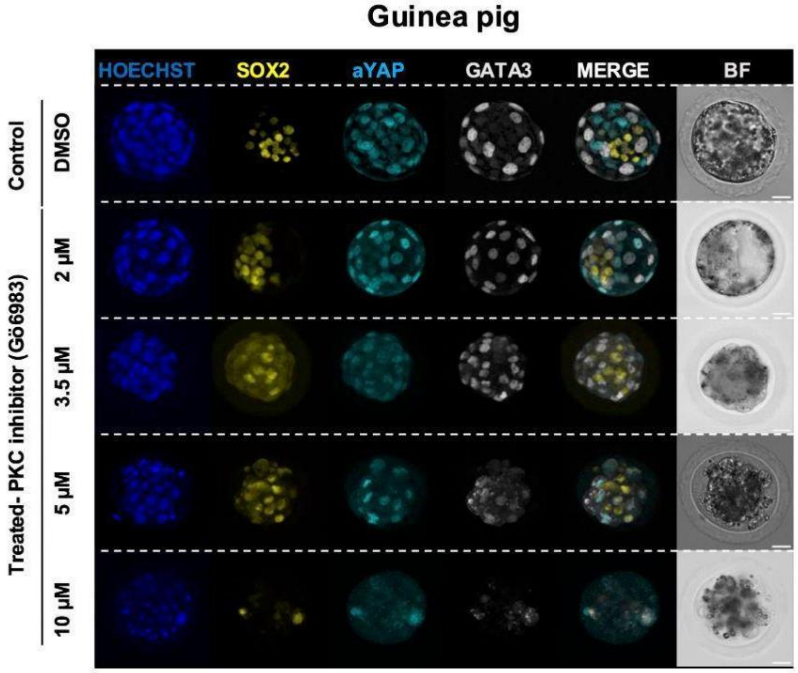

d

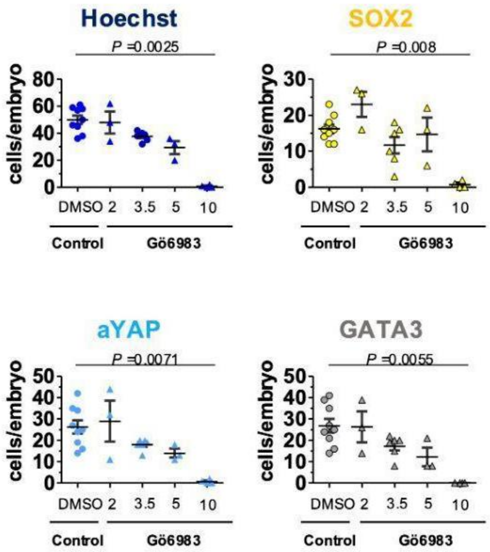

e

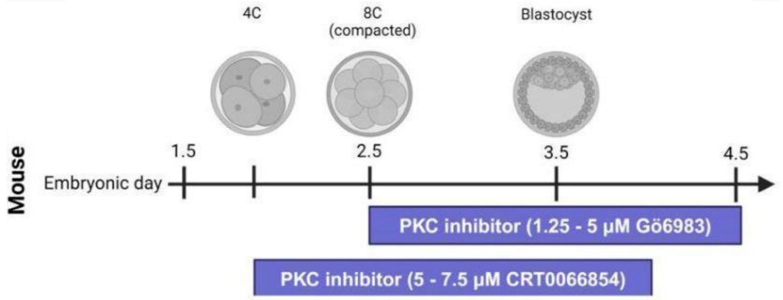

f

Medium	N	Number (%) of arrested embryos	Number (%) of morula	Number (%) of blastocyst
DMSO	8	0 (0)	2 (25)	6 (75)
1.25 µM Gö6983	5	0 (0)	2 (37)	3 (63)
2.5 µM Gö6983	7	0 (0)	2 (29)	5 (71)
3.75 µM Gö6983	5	0 (0)	5 (100)	0 (0)
5 µM Gö6983	5	5 (100)	0 (0)	0 (0)
DMSO	3	0 (0)	0 (0)	3 (100)
5 µM CRT0066854	3	0 (0)	1 (33)	2 (67)
7.5 µM CRT0066854	4	1 (25)	3 (75)	0 (0)

g

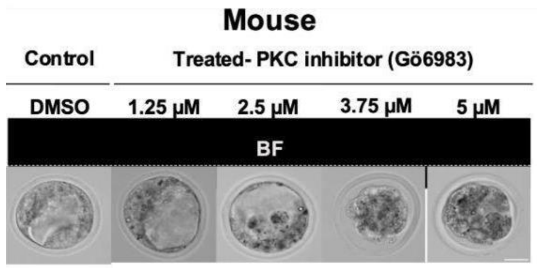

h

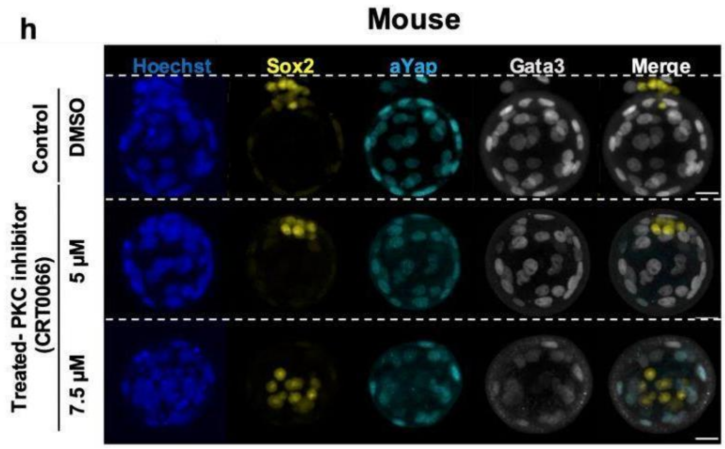

Supplementary Fig. 7 Dose-response of PKC inhibitor treatment in guinea pig and mouse embryos. a, Schematic of dose-response of PKC inhibitor (Gö6983) in guinea pigs. **b**, The percentage of guinea pig embryos either developing to form a blastocyst or arrested morula in control and Gö6983 treated embryos. **c**, Representative immunofluorescence image of SOX2 (yellow), aYAP (cyan), GATA3 (gray) and Hoechst nuclear staining (blue) in control (DMSO) and PKC inhibitor (Gö6983) treated guinea pig embryos from 16-cell compacted morula to mid-blastocyst. **d**, Scatter plot showing the total number of cells per embryo (Hoechst stained) and the number of cells per embryo for the indicated markers in control (n = 12) and Gö6983 treated embryos at 2 µM (n = 3), 3.5 µM (n = 6), 5 µM (n = 3) and 10 µM (n = 4). **e,** Schematic of dose-response of PKC inhibitors (Gö6983 and CRT0066854) in mice. **f**, The percentage of guinea pig embryos that developed to blastocyst or arrested at the morula stage in control and PKC inhibitors-treated embryos. **g,** Bright-field representative images of mouse embryos at E3.5 for control (n = 8) and Gö6983 treated at 1.25 µM (n = 5), 2.5 µM (n = 7), 3.75 µM (n = 5) and 5 µM (n = 5) µM. **h,** Representative immunofluorescence image of Sox2 (yellow), aYAP (cyan), Gata3 (gray) and Hoechst nuclear staining (blue) in control (n = 3) and CRT066854 treated mouse embryos at 5 µM (n = 3) and 7.5 µM (n = 4). P values stated in each figure (Kruskal-Wallis test with Dunn's post-test). Scatter plots with mean ± s.e.m. Schematic created in BioRender.com. Scale bars: 20 µm.

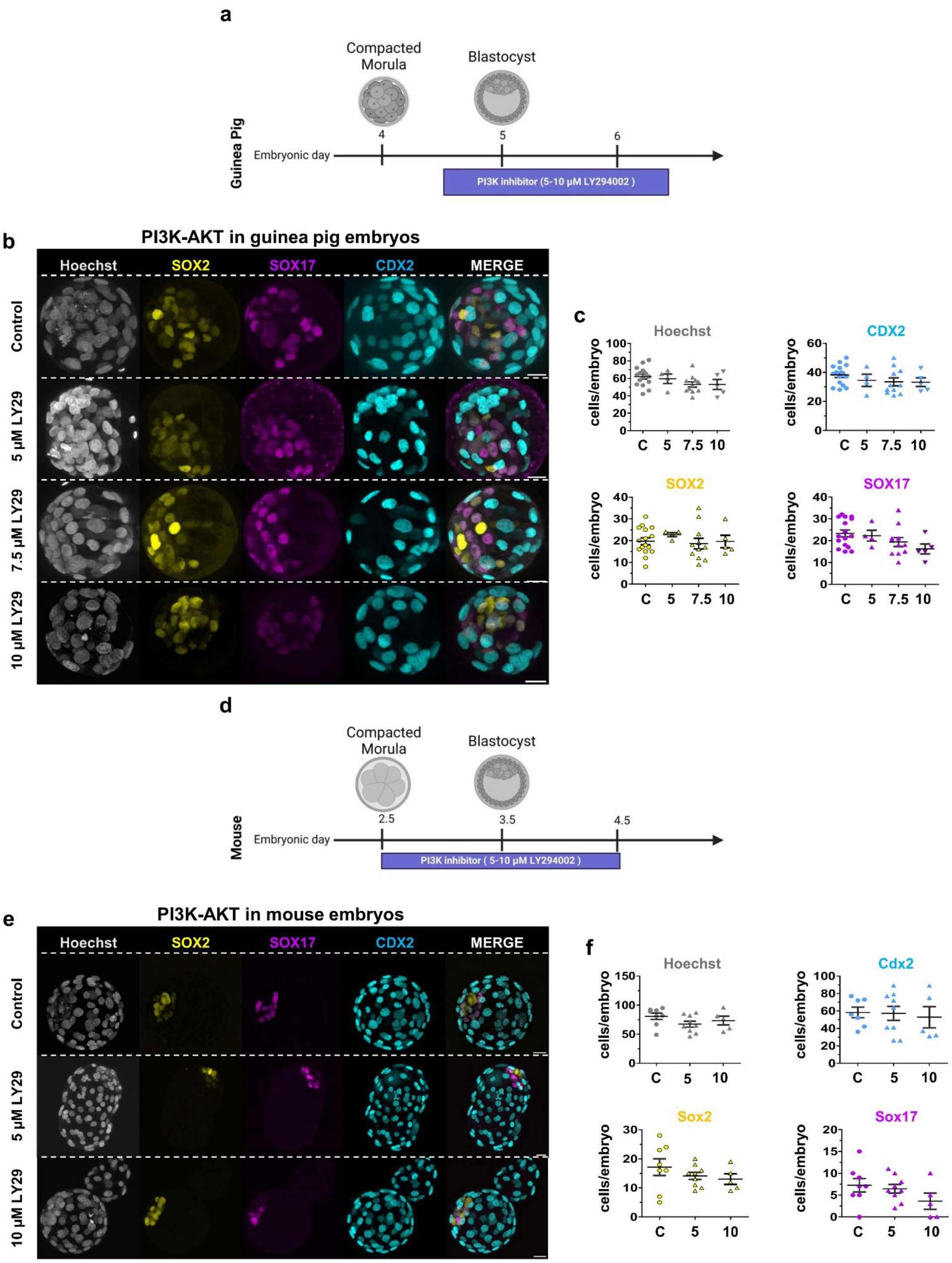

Supplementary Fig. 8 Dose-response of PI3K-AKT inhibitor in guinea pig and mouse embryos. **a**, Schematic of PI3K inhibitor (LY294002) treatment in guinea pig embryos. **b**, Representative immunofluorescence image of SOX2 (yellow), SOX17 (magenta), CDX2 (cyan) and Hoechst nuclear staining (gray) in control (DMSO) and LY29 treated guinea pig embryos for 48 h. **c**, Scatter plot showing the total number of cells per embryo (Hoechst stained) and the number of cells per embryo for the indicated markers in control embryos (n = 16) and LY29 treated embryos at 5 µM (n = 4), 7.5 µM (n = 11) and 10 µM (n = 5). **d**, Schematic of dose-response of PI3K inhibitor (LY294002) in mice. **e,** Representative immunofluorescence image of Sox2 (yellow), Sox17 (magenta), Cdx2 (cyan) and Hoechst nuclear staining (gray) in control (DMSO) and LY29 treated mouse embryos. **f**, Scatter plot showing the total number of cells per embryo (Hoechst stained)

and the number of cells per embryo for the indicated markers in control (n = 8) and LY29 treated embryos at 5 µM (n = 9) and 10 µM (n = 5). No significant differences (Kruskal-Wallis test). Schematic created in BioRender.com. Scatter plots with mean ± s.e.m. Scale bars: 20 µm.

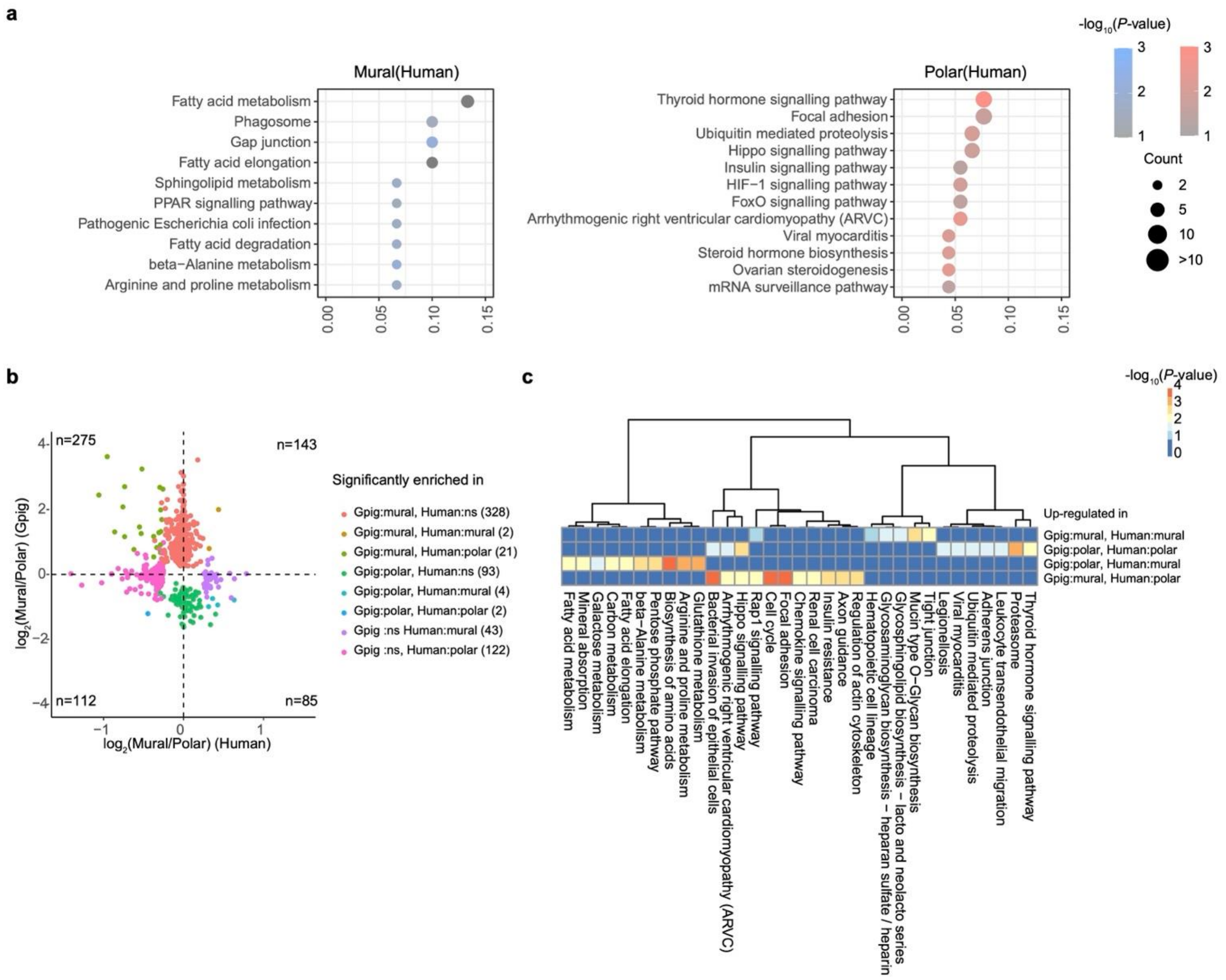

Supplementary Fig. 9 Expression of mural and polar DEGs in guinea pig and human mural and polar cells. **a**, Dot plot illustrating the enriched KEGG pathways for DEGs between mural and polar TE cells from Human (P < 0.05, one-sided, permutation test). Colour and size indicate the significance and number of differentially expressed genes in each pathway. **b**, Log2 fold change between mural and polar cells in human and guinea pig (x-axis and y-axis, respectively). Colours indicate the differential expression status in the guinea pig and human. The number of genes is indicated behind the colour legend. "n" in each corner represents the total number of genes in each quadrant. "ns" denotes "not significant." **c**, Heatmap illustrating the significance of enriched KEGG pathways for genes associated with differential expression status in guinea pig and human mural and polar cells.

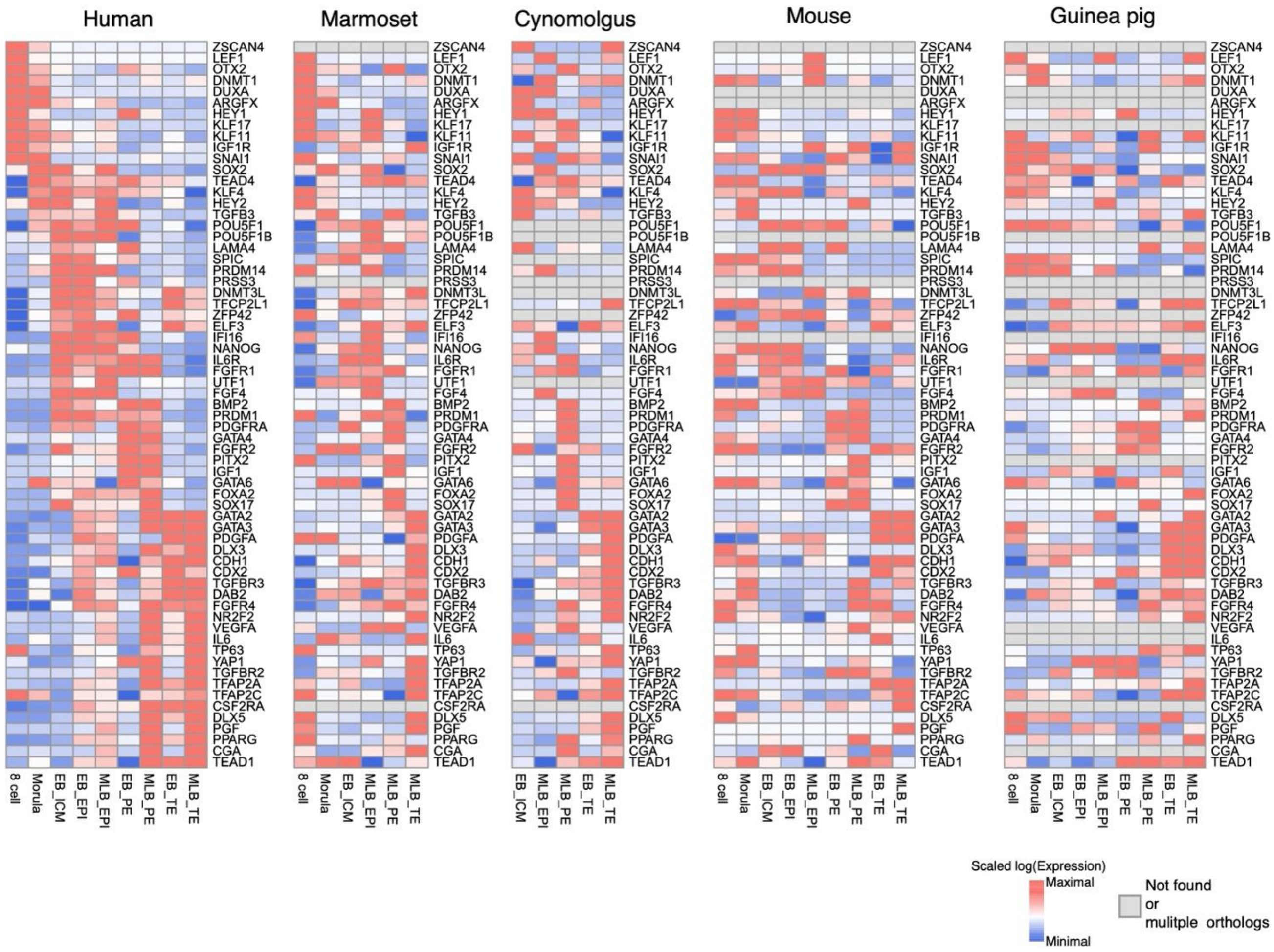

Supplementary Fig. 10: Heatmap depicting the pseudo-bulk expression of selected genes, categorized by stages and lineages, across human, marmoset, cynomolgus monkey, guinea pig, and mouse species. Gene selection was adapted from Meistermann et al., 2021 gene module analysis. Genes that could not be detected or with multiple orthologs in the corresponding species are coloured as gray. Preimplantation embryo data was leveraged from Petropoulos et al., 2016 (human), Nakamura et al., 2016 (cynomolgus monkey), Boroviak et al., 2018 (marmoset), Bergmann et al., 2022 (marmoset), Deng et al., 2014 (mouse), Nowotschin et al.,2019 (mouse), guinea pig data generated in this study.

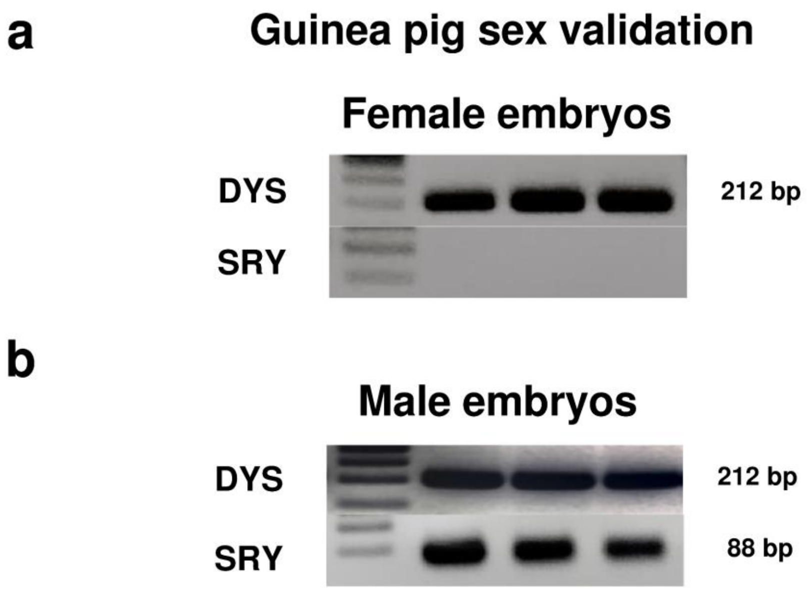

Supplementary Fig. 11: Guinea Pig Blastocysts Sex Validation. a and **b** Representative image of agarose gel following PCR of DYS and SRY genes for sex determination of three female (E1, E2 and E3) and three male (E4, E5 and E6) embryos used for H3Kme27 analysis. Amplicon of DYS is 212 bp and of SRY 88 bp.

Source Data:

Supplementary Figure 5c

Mouse TRULI dose-response
(Number of cells per embryo)

**Hoechst**				
**DMSO**	**2 µM TRULI**	**5 µM TRULI**	**10 µM TRULI**	**20 µM TRULI**
45	44	29	15	15
35	16	37	32	12
36	34	30	25	14
36				
38				

**Sox2**				
**DMSO**	**2 µM TRULI**	**5 µM TRULI**	**10 µM TRULI**	**20 µM TRULI**
18	0	0	0	0
10	0	0	0	0
6	0	0	0	0
12				
15				

**aYap**				
**DMSO**	**2 µM TRULI**	**5 µM TRULI**	**10 µM TRULI**	**20 µM TRULI**
28	32	26	15	14
23	15	37	32	12
18	26	29	25	13
14				
16				

**Gata3**				
**DMSO**	**2 µM TRULI**	**5 µM TRULI**	**10 µM TRULI**	**20 µM TRULI**
27	32	23	15	1
17	15	37	32	8
20	25	29	25	1
14				
16				

Supplementary Figure 6c

Mouse
Hippo signalling inhibition with optimal dose at two different time-points
From 8C to Blastocyst E3.5
(Number of cells per embryo)

Hoechst	
DMSO	5 µM TRULI
35	37
36	54
32	30
34	27
	46

Gata3			
Outer		Inner	
DMSO	5 µM TRULI	DMSO	5 µM TRULI
17	43	0	10
18	32	0	5
20	23	0	4
24	21	0	6
	33		7
	26		6

Sox2			
Outer		Inner	
DMSO	5 µM TRULI	DMSO	5 µM TRULI
0	0	10	0
0	0	6	0
0	0	8	0
0	0	10	0
	0		1
	0		0

aYap			
Outer		Inner	
DMSO	5 µM TRULI	DMSO	5 µM TRULI
17	23	0	4
15	32	0	5
18	40	0	9
22	21	0	6
	35		6
	26		6

Supplementary Figure 6e

From 16C to Blastocyst E4.5

(Number of cells per embryo)

**Hoechst**	
**DMSO**	**5 µM TRULI**
105	72
96	88
84	103
84	86
106	98
111	82
	90

**Gata3**			
**Outer**		**Inner**	
**DMSO**	**5 µM TRULI**	**DMSO**	**5 µM TRULI**
75	63	0	6
80	80	0	8
59	95	0	8
58	72	0	10
88	68	0	12
96	65	0	10
	73		9

**Sox2**			
**Outer**		**Inner**	
**DMSO**	**5 µM TRULI**	**DMSO**	**5 µM TRULI**
0	0	16	2
0	0	13	0
0	0	11	2
0	0	25	0
0	0	9	0
0	0	13	0
	0		0

**aYap**			
**Outer**		**Inner**	
**DMSO**	**5 µM TRULI**	**DMSO**	**5 µM TRULI**
74	62	0	8
71	93	0	7
60	82	0	6
53	62	0	10
89	56	0	13
96	63	0	10
	71		9

Supplementary Figure 6g

MAPK signalling inhibition (PD0325901) with optimal dose

(Number of cells per embryo)

**Hoechst**	
**DMSO**	**1 µM PD032**
171	146
105	109
88	115
90	
108	
95	

**Cdx2**	
**DMSO**	**1 µM PD032**
124	99
78	98
60	82
60	
80	
68	

**Sox2**	
**DMSO**	**1 µM PD032**
23	47
11	29
16	33
17	
14	
15	

**Sox17**	
**DMSO**	**1 µM PD032**
24	0
16	0
12	0
13	
14	
12	

Supplementary Figure 7b

Medium	N	Number (%) of arrested embryos	Number (%) of morula	Number (%) of blastocyst
**DMSO**	12	1 (8)	2 (17)	9 (75)
**2 µM Gö6983**	3	0 (0)	1 (33)	2 (67)
**3.5 µM Gö6983**	6	0 (0)	6 (100)	0 (0)
**5 µM Gö6983**	3	0 (0)	3 (100)	0 (0)
**10 µM Gö6983**	4	3 (75)	0 (0)	1 (25)

Supplementary Figure 7d

Guinea pig Dose-response of PKC inhibitor (Gö6983)

(Number of cells per embryo)

**Hoechst**				
**DMSO**	**2 µM**	**3.5 µM**	**5 µM**	**10 µM**
58	62	35	20	2
57	48	39	32	0
59	34	40	36	1
50		38		0
45		42		
36		32		
46				
38				
61				

**SOX2**				
**DMSO**	**2 µM**	**3.5 µM**	**5 µM**	**10 µM**
16	26	3	6	2
12	27	8	16	0
16	16	15	22	1
18		16		0
23		18		
12		10		
14				
15				
20				

**aYAP**				
**DMSO**	**2 µM**	**3.5 µM**	**5 µM**	**10 µM**
25	32	18	13	2
42	44	20	18	1
35	11	13	11	0
27		19		0
19		18		
14		20		
24				
16				
34				

**GATA3**				
**DMSO**	**2 µM**	**3.5 µM**	**5 µM**	**10 µM**
25	26	22	8	0
39	39	20	21	0
35	14	8	8	0
27		19		0
21		20		
14		15		
24				
16				
41				

Supplementary Figure 7e

Mouse PKC inhibitor Dose-response

Medium	N	Number (%) of arrested embryos	Number (%) of morula	Number (%) of blastocyst
**DMSO**	8	0 (0)	2 (25)	6 (75)
**1.25 µM Gö6983**	5	0 (0)	2 (37)	3 (63)
**2.5 µM Gö6983**	7	0 (0)	2 (29)	5 (71)
**3.75 µM Gö6983**	5	0 (0)	5 (100)	0 (0)
**5 µM Gö6983**	5	5 (100)	0 (0)	0 (0)

**DMSO**	3	0 (0)	0 (0)	3 (100)
**5 µM CRT0066**	3	0 (0)	1 (33)	2 (67)
**7.5 µM CRT0066**	4	1 (25)	3 (75)	0 (0)

Supplementary Figure 8c

Guinea Pig PI3K Dose-response
Inhibition (LY294002) for 48h
(Number of cells per embryo)

Hoechst			
**DMSO**	**5 µM LY29**	**7.5 µM LY29**	**10 µM LY29**
81	69	75	68
78	44	61	44
72	65	45	38
42	60	46	64
66		48	51
64		38	
65		46	
58		57	
62		56	
61		58	
69		52	
67			
53			
56			
46			
52			

CDX2			
**DMSO**	**5 µM LY29**	**7.5 µM LY29**	**10 µM LY29**
40	37	50	32
43	24	45	28
50	44	31	27
29	33	28	36
39		28	43
31		25	
39		41	
33		26	
28		39	
40		24	
37		31	
47			
29			
45			
37			
44			

SOX2			
**DMSO**	**5 µM LY29**	**7.5 µM LY29**	**10 µM LY29**
31	24	35	30
27	20	31	16
18	24	12	17
22	23	19	14
26		11	21
26		14	
8		9	
15		18	
20		16	
25		23	
17		17	
12			
17			
21			
16			
15			

SOX17			
**DMSO**	**5 µM LY29**	**7.5 µM LY29**	**10 µM LY29**
31	29	34	24
32	17	28	15
20	21	14	15
21	22	21	10
24		15	17
31		14	
22		10	
15		18	
17		18	
30		21	
31		20	
28			
18			
22			
16			
15			

Supplementary Figure 8f

Mouse PI3K Dose-response Inhibition (LY294002) for 48h

(Number of cells per embryo)

Hoechst		
**DMSO**	**5 µM LY29**	**10 µM LY29**
89	70	76
82	87	60
91	85	54
92	51	81
49	56	96
67	65	
81	63	
95	46	
	82	

Cdx2		
**DMSO**	**5 µM LY29**	**10 µM LY29**
72	64	37
73	79	32
52	72	31
56	89	75
42	26	89
36	41	
77	41	
	26	
	77	

Sox2		
**DMSO**	**5 µM LY29**	**10 µM LY29**
28	15	12
16	9	19
24	13	9
23	10	10
5	18	15
16	15	
18	20	
7	16	
	11	

Sox17		
**DMSO**	**5 µM LY29**	**10 µM LY29**
15	7	3
10	8	0
5	10	0
7	11	10
0	5	5
5	3	
9	2	
7	6	
	6	

Supplementary Figure 11

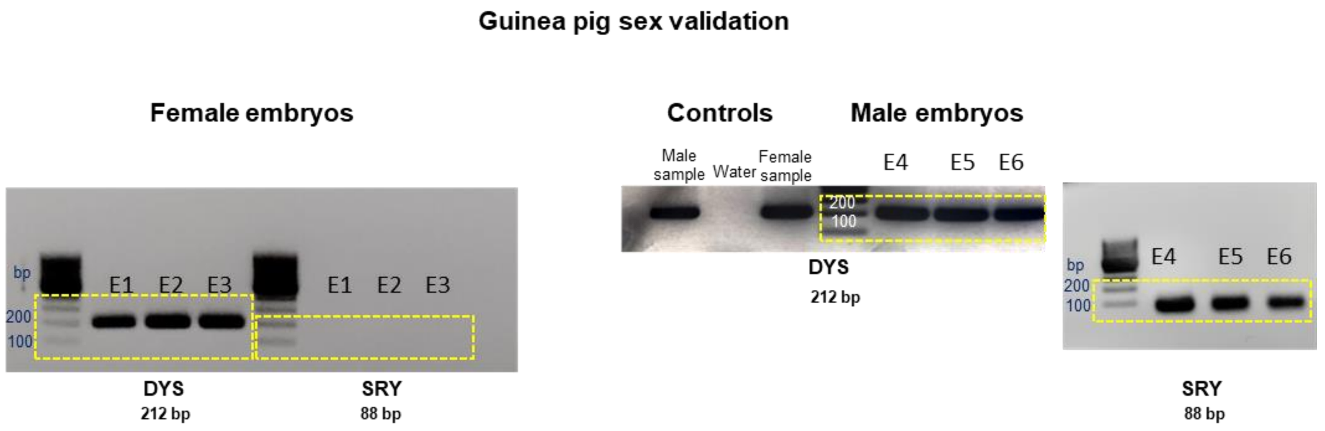

Supplement: Supplementary file 1 — Supplementary Figs. 1–11 and source data for Supplementary Figs. 5–8 and 11. [file 41556_2025_1642_MOESM1_ESM.pdf]
