# Supplementary material for: The guinea pig serves as an alternative model to study human preimplantation development
Source: Nat Cell Biol. 2025 Apr 4;27(4):696–710. doi: 10.1038/s41556-025-01642-9 (PMC11991919; doi:10.1038/s41556-025-01642-9)
Supplement: Supplementary file 2 — Reporting Summary [file 41556_2025_1642_MOESM2_ESM.pdf]

nature portfolio

Corresponding author(s): Sophie Petropoulos

Last updated by author(s): Feb 12, 2025

Reporting Summary

Nature Portfolio wishes to improve the reproducibility of the work that we publish. This form provides structure for consistency and transparency in reporting. For further information on Nature Portfolio policies, see our Editorial Policies and the Editorial Policy Checklist.

Statistics

For all statistical analyses, confirm that the following items are present in the figure legend, table legend, main text, or Methods section.

n/a	Confirmed	
☐	☒	The exact sample size (*n*) for each experimental group/condition, given as a discrete number and unit of measurement
☐	☒	A statement on whether measurements were taken from distinct samples or whether the same sample was measured repeatedly
☐	☒	The statistical test(s) used AND whether they are one- or two-sided<br>*Only common tests should be described solely by name; describe more complex techniques in the Methods section.*
☐	☒	A description of all covariates tested
☐	☒	A description of any assumptions or corrections, such as tests of normality and adjustment for multiple comparisons
☐	☒	A full description of the statistical parameters including central tendency (e.g. means) or other basic estimates (e.g. regression coefficient) AND variation (e.g. standard deviation) or associated estimates of uncertainty (e.g. confidence intervals)
☐	☒	For null hypothesis testing, the test statistic (e.g. *F*, *t*, *r*) with confidence intervals, effect sizes, degrees of freedom and *P* value noted<br>*Give P values as exact values whenever suitable.*
☒	☐	For Bayesian analysis, information on the choice of priors and Markov chain Monte Carlo settings
☐	☒	For hierarchical and complex designs, identification of the appropriate level for tests and full reporting of outcomes
☐	☒	Estimates of effect sizes (e.g. Cohen's *d*, Pearson's *r*), indicating how they were calculated

Our web collection on statistics for biologists contains articles on many of the points above.

Software and code

Policy information about availability of computer code

Data collection	no software except "wget" command was used for data collection
Data analysis	Published software included:<br>HISAT2 aligner (v 2.2.0), featureCounts (v.1.6.3), Stringtie (v1.3.3b), gffcompare (v.0.12.6), R package Seurat (v.4.2.0), R package clustree (v.0.4.3), R package clusterProfiler (v3.18.1), R package Monocle2 (v.2.14.0), Monocle3 (v1.0.0), R package SeuratWrappers (v.0.3.0) , R package batchelor (v.1.6.2). R package SCP (v0.5.6). GraphPad Prism (v9.2.0).

For manuscripts utilizing custom algorithms or software that are central to the research but not yet described in published literature, software must be made available to editors and reviewers. We strongly encourage code deposition in a community repository (e.g. GitHub). See the Nature Portfolio guidelines for submitting code & software for further information.

Data

Policy information about availability of data

All manuscripts must include a data availability statement. This statement should provide the following information, where applicable:

- Accession codes, unique identifiers, or web links for publicly available datasets
- A description of any restrictions on data availability
- For clinical datasets or third party data, please ensure that the statement adheres to our policy

All guinea pig raw and processed sequencing data generated in this study have been submitted to GEO under accession numbers GSE253670 (https://www.ncbi.nlm.nih.gov/geo/query/acc.cgi?acc=GSE253670).

Other published datasets include:
1) two human embryonic datasets, Yanagida et al. 2021(GSE171820, https://www.ncbi.nlm.nih.gov/geo/query/acc.cgi?acc=GSE171820); Petropoulos et al. 2016(E-MTAB-3929, https://www.ebi.ac.uk/arrayexpress/experiments/E-MTAB-3929). 2) we included two embryonic datasets from Callithrix jacchus (marmoset) (Bergmann et al. 2022(E-MTAB-9367, https://www.ebi.ac.uk/biostudies/arrayexpress/studies/E-MTAB-9367); Boroviak et al. 2018(E-MTAB-7078, https://www.ebi.ac.uk/arrayexpress/experiments/E-MTAB-7078)). 3) one embryonic datasets from Macaca fascicularis (Crab-eating macaque)(Nakamura et al. 2016(GSE74767, https://www.ncbi.nlm.nih.gov/geo/query/acc.cgi?acc=GSE74767). 4) three mouse embryonic datasets Deng et al., 2014(GSE45719, https://www.ncbi.nlm.nih.gov/geo/query/acc.cgi?acc=GSE45719), Posfai et al., 2017(GSE84892, https://www.ncbi.nlm.nih.gov/geo/query/acc.cgi?acc=GSE84892), and Nowotschin et al., 2019(GSE123046, https://www.ncbi.nlm.nih.gov/geo/query/acc.cgi?acc=GSE123046).

Human research participants

Policy information about studies involving human research participants and Sex and Gender in Research.

Reporting on sex and gender	Embryo sex was considered for the X-inactivation experiments.
Population characteristics	Embryos were de-identified. Human embryos samples were donated blinded (we do not have any information available about the donors, then no covariate-relevant population characteristics is available).
Recruitment	Patients of Clinique OVO which had previously indicated an interest in donating surplus embryos to research were contacted by phone to obtain informed written consent. Consent was obtained from both parents of all couples that donated spare embryos following IVF treatment. Embryos received were de-identified. No financial compensation was offered for donations.
Ethics oversight	Human embryos were obtained from the Clinique OVO with ethical approval from the regional ethics boards at the CRCHUM and Université de Montréal (CERSES-20-107-R and 20.126). Embryos were not cultured beyond E7. Experiments were conducted in compliance with guidelines from the Ministère de la Santé et des Services sociaux, the International Society for Stem Cell Research, and the regional ethics boards.

Note that full information on the approval of the study protocol must also be provided in the manuscript.

Field-specific reporting

Please select the one below that is the best fit for your research. If you are not sure, read the appropriate sections before making your selection.

☒ Life sciences ☐ Behavioural & social sciences ☐ Ecological, evolutionary & environmental sciences

For a reference copy of the document with all sections, see nature.com/documents/nr-reporting-summary-flat.pdf

Life sciences study design

All studies must disclose on these points even when the disclosure is negative.

Sample size	For RNAseq part: Data distribution was assumed to be normal, but not formally tested. Sample size was similar to those reported in previous publications using rabbit(PMID:35993311) or mouse embryos(PMID: 28226240). For functional analysis: no statistical methods were used to pre-determine sample sizes, but our sample size are similar to those reported in similar studies such as: Gerri et al 2020 for human, mouse and cow embryos (PMID: 32968278) and Gerri et al 2023 for rat, human and mouse embryos (https://doi.org/10.1242/dev.201112)
Data exclusions	For RNAseq: Low-quality libraries and cells that were excluded are described in Methods. In the functional analysis, specific stages were analyzed. For example, from E5 onwards, we analyzed blastocysts but excluded delayed morulas—except in the case of the PKC inhibitor treatment, which delays development; in that case, no exclusions were made.
Replication	Each experiment was performed independently at least three times
Randomization	Embryos collected per dam were arbitrarily allocated to experiments to avoid any litter effects.

Blinding	The investigators were aware of group allocation during the experiments; however, cell counting and intensity measurements were performed in a blinded manner for each embryo using the embryo ID (composed of the year, mother ID, and embryo number). Embryos were reassigned to their respective groups when creating the graphs.

Reporting for specific materials, systems and methods

We require information from authors about some types of materials, experimental systems and methods used in many studies. Here, indicate whether each material, system or method listed is relevant to your study. If you are not sure if a list item applies to your research, read the appropriate section before selecting a response.

Materials & experimental systems

n/a	Involved in the study
☐	☒ Antibodies
☒	☐ Eukaryotic cell lines
☒	☐ Palaeontology and archaeology
☐	☒ Animals and other organisms
☒	☐ Clinical data
☒	☐ Dual use research of concern

Methods

n/a	Involved in the study
☒	☐ ChIP-seq
☒	☐ Flow cytometry
☒	☐ MRI-based neuroimaging

Antibodies

Antibodies used

List of antibodies used available below:
SOX2: 14981182 or 740013T Rat (monoclonal, Btjce clone) Thermofisher Scientific 1/50 for guinea pigs, for human and mouse 1/200
GATA6 AF1700-SP Goat (polyclonal) R&D System 1/100
SOX17 AF1924-SP Goat (polyclonal) R&D system 1/200
Active YAP ab205270 Rabbit (monoclonal, EPR19812 clone number) Abcam 1/200
GATA3 Mouse (monoclonal, CM405A) Biocare Medical 1/250
CDX2 Mouse (monoclonal, MU392A-5UC) Biogenex 1/250
H3K27me3 3184297 Rabbit (polyclonal) Active Motif also sells by Thermofisher Scientific 1/500
NR2F2 ab211776 Rabbit (monoclonal, EPR18442) Abcam 1/100
RXRa sc-515929 Mouse (monoclonal, H-10) Santa Cruz 1/100
Note: Supplementary Table 11 also has an extended list of functional and non functional antibodies tested in our laboratory with guinea pigs embryos.

Validation

Antibodies were selected from previous work (https://doi.org/10.1186/s12861-019-0193-9; https://doi.org/10.1038/s41467-019-08387-8, doi: 10.1016/j.stem.2021.04.027, DOI: 10.1038/355359a0, https://doi.org/10.1038/s41586-020-2759-x). The antibody was tested in mouse and guinea pig embryos and visualized for correct positional location within the cell and embryo.

Animals and other research organisms

Policy information about studies involving animals; ARRIVE guidelines recommended for reporting animal research, and Sex and Gender in Research

Laboratory animals	Hartley guinea pigs (Charles River Labs), C57BL/6 mice (Charles River labs)
Wild animals	Study did not involve wild animals.
Reporting on sex	Sex was considered for the X-Chromosome inactivation portion of the study.
Field-collected samples	Study did not involve field-collected samples.
Ethics oversight	All procedures involving animals were approved by the Comité Institutionnel de Protection des Animaux (CIPA), IP19022SPci and IP21005SPSs for guinea pig and mouse.

Note that full information on the approval of the study protocol must also be provided in the manuscript.
